# Development and external validation of prognostic models for COVID-19 to support risk stratification in secondary care

**DOI:** 10.1136/bmjopen-2021-049506

**Published:** 2022-01-17

**Authors:** Nicola J Adderley, Thomas Taverner, Malcolm James Price, Christopher Sainsbury, David Greenwood, Joht Singh Chandan, Yemisi Takwoingi, Rashan Haniffa, Isaac Hosier, Carly Welch, Dhruv Parekh, Suzy Gallier, Krishna Gokhale, Alastair K Denniston, Elizabeth Sapey, Krishnarajah Nirantharakumar

**Affiliations:** 1 Institute of Applied Health Research, University of Birmingham, Birmingham, UK; 2 NIHR Birmingham Biomedical Research Centre, University Hospitals Birmingham NHS Foundation Trust, Birmingham, UK; 3 Department of Diabetes, Gartnavel General Hospital, Glasgow, UK; 4 Mahidol Oxford Tropical Medicine Research Unit, University of Oxford, Oxford, UK; 5 Centre for Anaesthesia Critical Care & Pain Medicine, University College London Hospitals NHS Foundation Trust, London, UK; 6 Institute of Inflammation and Ageing, University of Birmingham, Birmingham, UK; 7 University Hospitals Birmingham NHS Foundation Trust, Birmingham, UK; 8 National Institute for Health Research Biomedical Research Centre, Moorfields Eye Hospital NHS Foundation Trust, London, UK; 9 Health Data Research UK, London, UK

**Keywords:** public health, public health, COVID-19

## Abstract

**Objectives:**

Existing UK prognostic models for patients admitted to the hospital with COVID-19 are limited by reliance on comorbidities, which are under-recorded in secondary care, and lack of imaging data among the candidate predictors. Our aims were to develop and externally validate novel prognostic models for adverse outcomes (death and intensive therapy unit (ITU) admission) in UK secondary care and externally validate the existing 4C score.

**Design:**

Candidate predictors included demographic variables, symptoms, physiological measures, imaging and laboratory tests. Final models used logistic regression with stepwise selection.

**Setting:**

Model development was performed in data from University Hospitals Birmingham (UHB). External validation was performed in the CovidCollab dataset.

**Participants:**

Patients with COVID-19 admitted to UHB January–August 2020 were included.

**Main outcome measures:**

Death and ITU admission within 28 days of admission.

**Results:**

1040 patients with COVID-19 were included in the derivation cohort; 288 (28%) died and 183 (18%) were admitted to ITU within 28 days of admission. Area under the receiver operating characteristic curve (AUROC) for mortality was 0.791 (95% CI 0.761 to 0.822) in UHB and 0.767 (95% CI 0.754 to 0.780) in CovidCollab; AUROC for ITU admission was 0.906 (95% CI 0.883 to 0.929) in UHB and 0.811 (95% CI 0.795 to 0.828) in CovidCollab. Models showed good calibration. Addition of comorbidities to candidate predictors did not improve model performance. AUROC for the International Severe Acute Respiratory and Emerging Infection Consortium 4C score in the UHB dataset was 0.753 (95% CI 0.720 to 0.785).

**Conclusions:**

The novel prognostic models showed good discrimination and calibration in derivation and external validation datasets, and performed at least as well as the existing 4C score using only routinely collected patient information. The models can be integrated into electronic medical records systems to calculate each individual patient’s probability of death or ITU admission at the time of hospital admission. Implementation of the models and clinical utility should be evaluated.

**Strengths and limitations of this study**

- The University Hospitals Birmingham (UHB) development dataset represents one of the largest and most ethnically diverse patient cohorts within the UK.
- As part of the UHB COVID-19 response, all admitted patients underwent a wide range of investigations to support international research efforts examining prognostic markers allowing assessment of a wide range of possible predictors (demographic variables, symptoms, physiological measures, imaging and laboratory test results) with low levels of missing data.
- A limitation of the study was that the overall sample size was relatively small compared with that of the International Severe Acute Respiratory and Emerging Infection Consortium study and was limited to one UK geographical location.
- In the external validation cohort, we were unable to examine all of the predictors included in the original full UHB model due to only a reduced set of candidate predictors being available in CovidCollab.
- It was not possible to carry out stratified analysis by ethnicity as the UHB dataset contained too few patients in many of the strata, and no ethnicity data were available in the CovidCollab dataset.

## Background

The COVID-19 pandemic has placed exceptional strain on healthcare systems globally. Health systems, and especially critical care services, can be overwhelmed, given the number of patients and the duration and severity of their illness. A proportion of patients with COVID-19 can deteriorate rapidly. Clinicians need to differentiate between those with COVID-19 who are at high risk of the most severe symptoms (requiring intensive care treatment/ventilation) or death, and those who can be considered at low risk and potentially managed in the community. Early identification of patients at highest risk of severe outcomes may provide opportunity to prioritise, intervene and improve outcomes.

Objective prognostic tools for patients with COVID-19, based on patients’ initial characteristics, symptoms, biomarkers and imaging at the time of hospital admission, which can be used at or just after admission, and which can accurately discriminate between patients who will progress to more severe symptoms or death and those who will not, can be used by clinicians to triage and manage patients. This could potentially reduce time to appropriate interventions and improve patient outcomes.

A rapid systematic review has identified a number of prediction models developed for COVID-19, including prognostic models.^1^ However, while these existing studies provided useful information on candidate predictors for further exploration, the review found substantial limitations: many models were developed exclusively in a Chinese population; many were at high risk of bias, particularly in terms of inclusion of non-representative control participants, inappropriate exclusion criteria and small sample sizes, leading to high risk of overfitting; and external validation was limited.^1^ Other studies have evaluated existing early warning scores such as the National Early Warning Score, but with conflicting findings regarding their utility in predicting COVID-19 outcomes.^2 3^

More recent models have since been developed,^4 5^ some of which overcome a number of these limitations, including the International Severe Acute Respiratory and Emerging Infection Consortium (ISARIC) model and corresponding (simplified) 4C score, which was developed in a UK secondary care population representing 260 hospitals in England, Scotland and Wales (the ISARIC dataset).^5^ While the 4C score showed reasonable discrimination for mortality, there are some limitations, including a reliance on clinicians counting specific comorbidities, which may not be recorded at admission and which are known to be under-recorded in secondary care,^6^ and an absence of imaging data among the candidate predictors.

### Aims and rationale

To date, there have been few prognostic models for patients admitted to the hospital with COVID-19 developed in a UK dataset. Furthermore, evaluation of the extent to which the inclusion of comorbidities, imaging and additional biomarkers improves model performance is required. It also remains to be determined whether updating the clinical parameters with evolving biomarkers improves prediction of the clinical course of patients as the disease evolves.

The overarching aim of this study was to develop prognostic models for patients admitted to the hospital with COVID-19 using routinely collected data at the point of admission, which can be used in a secondary care setting to support clinical decision-making. Specific objectives were (1) to develop novel prognostic models for calculating predicted probability of adverse outcomes (death and intensive therapy unit (ITU) admission) at an individual patient level in a UK secondary care setting; (2) to externally validate these models in an international dataset (including data from UK hospitals); (3) to externally validate the existing UK ISARIC 4C score^5^; and (4) to compare performance of the newly developed models with the UK ISARIC 4C score. In addition, we developed daily models using time series data from the first 8 days from admission to explore changes in predictors over time.

## Methods

### Data source

Data from University Hospitals Birmingham (UHB) NHS Foundation Trust were sourced via the PIONEER Health Data Research Hub for acute care and were used for model development and for external validation of the ISARIC 4C score. Data from patients with COVID-19 admitted to Queen Elizabeth Hospital, Birmingham (part of UHB), between 1 January 2020 and 16 August 2020 were included. Data included symptoms recorded at admission, comorbidities (from International Classification of Diseases, 10th revision (ICD-10) discharge codes), vital signs (eg, blood pressure and oxygen saturation), laboratory results (biochemistry, haematology, microbiology and pathology), imaging and outcomes (ITU admission and death).

External validation of the newly developed models was performed in the CovidCollab dataset. CovidCollab is an international project using routinely collected healthcare data to develop a better understanding of how best to treat and care for adults with COVID-19.^7 8^ The dataset includes symptoms, comorbidities, vital signs, laboratory results, imaging findings and outcomes.

### Study population

Patients of all ages diagnosed with COVID-19 and hospitalised were included. Diagnosis was defined as a positive test result for SARS-CoV-2 from one or more reverse transcription PCR or transcription-mediated amplification tests. In the CovidCollab dataset, COVID-19 diagnosis was by either PCR or antibody test. Anonymised data for all patients with COVID-19 admitted to UHB during the study period were included. For CovidCollab, data collection was dependent on the specific processes within individual participating hospitals and the capacity of the data collector.^7^

### Study design

The study utilised retrospective cohort analyses; the index date (start of follow-up) was the hospital admission date. The study period was from 1 January 2020 to 12 September 2020 (the last admission date was 16 August to ensure a minimum of 28 days of follow-up).

### Outcomes

The primary outcome was death within 28 days of admission (in-hospital or post-discharge). The secondary outcome was ITU admission within 28 days of admission.

### Study follow-up

Participants were followed up from index (admission) date until the earliest of outcome date or study end (latest available data, 12 September 2020). Participants were censored 28 days after the index date. Participants admitted after 16 August 2020 (less than 28 days prior to the study end date) were excluded.

### Candidate predictor variables

Candidate predictors were selected a priori following a review of existing literature, discussion with clinical experts (specialists in acute care, critical care and geriatric medicine), and based on availability of variables routinely collected in secondary care/UHB. These included demographic variables, symptoms, comorbidities, physiological measures, imaging findings and laboratory test results. Comorbidities are not reliably and completely collected at admission, with the most complete hospital record of comorbidities usually being the discharge ICD-10 codes; therefore, the development and performance of models with and without comorbidity predictors were compared in order to explore the potential for developing models which would require no additional data collection (other than routinely collected data) at the point of admission.

### Model development

Models were trained using UHB data (patients admitted up to and including 16 August 2020). We used a multistage model building process that assessed the impact of a range of feature representation and modelling choices to select important candidate predictors. All analyses were performed in R.

Three sets of models were fitted which incorporated continuous variables in three different ways, to explore the impact of treating these variables as continuous or categorical, and also to explore the impact of different methods of handling missing data:

- As continuous numeric values, with missing values imputed (‘continuous’).
- As categorical values derived from the imputed continuous values (‘categorical-imputed’).
- In secondary analysis, as categorical values, using clinically meaningful categories and reference ranges, with missing indicators as a separate category (‘categorical’).

For the three ways of handling numerical features and missing variables mentioned previously, we fitted outcomes of death within 28 days and ITU admission (within 28 days) to candidate predictors using a range of models, which allowed both linear relationships and complex interactions between variables:

- Logistic regression with (1) all baseline parameters (demographic variables, symptoms, vital signs/physiological measures and laboratory test results); (2) demographic variables only; and (3) all baseline parameters with the addition of recorded comorbidities (recorded up to the point of discharge).
- Logistic regression with stepwise Akaike information criterion (AIC) minimisation, both forward and backward.^9^
- Least absolute shrinkage and selection operator (LASSO, l1 penalised) logistic regression using all baseline parameters.
- Gradient boosted model (GBM) using all baseline parameters with default hyperparameter values of 150 trees, maximum interaction depth of 3, minimum of 10 observations in nodes and shrinkage of 0.1.^10^

Further information on handling of continuous variables is presented in online supplemental appendix 1.

10.1136/bmjopen-2021-049506.supp1 Supplementary data

For each of these four variable selection models, in order to reduce overfitting and selection bias, we internally validated using fivefold cross-validation (80/20 train/test split) to derive the candidate variable list. To avoid sensitivity to imputation, this cross-validation was repeated for each of the five multiple imputations.

Due to the relatively small number of outcome events (<300), we did not attempt to systematically look for interactions between multiple variables.

#### Model performance

Model performance (discrimination) was assessed by calculating the area under the receiver operating characteristic curve (AUROC or C-statistic).^11^ Calibration was assessed by plotting the observed probability of the outcome against predicted probability and by calculating the calibration slope and intercept. We also calculated sensitivity, specificity, positive predictive value (PPV) and negative predictive value (NPV) for the final models. For each feature set and each model, the final results for cross-validated (optimism-adjusted) AUROC and all other metrics (including calibration plots) were combined from all the multiple imputations of the dataset using Rubin’s rules for the mean and CI (derived from the SD).^12^

#### Missing data

Information on candidate predictors was collected at the point of admission; however, where information on physiological or laboratory measures was not available on the day of admission, measures recorded up to 72 hours after admission were used. Candidate predictors for which >40% of patients had missing data were excluded from the analysis. Further missing continuous variables (vital signs and laboratory tests) and symptoms were imputed using multiple imputation using chained equations (using the R ‘mice’ multiple imputation package). We performed five imputations and a maximum of 50 iterations.^13^ Continuous variables were imputed with predictive mean matching, and categorical variables with logistic regression (logreg) or polytomous regression (polyreg). Input variables for the multiple imputation included all available candidate predictor variables in the dataset; outcomes were not included in the imputation variables.

We also explored use of a missing category for missing test results. Absence of a record of a comorbidity was taken to indicate absence of the condition.

#### External validation

To investigate the transferability of models, we performed external validation of logistic regression models derived from the UHB dataset in the CovidCollab dataset for predicting outcomes of 28-day mortality and ITU admission.

Not all candidate predictors were common to both datasets; therefore, new logistic regression models for death within 28 days and for ITU admission were refitted on the UHB data using only those variables also present in the CovidCollab data. We then performed an external validation of these UHB models in the CovidCollab dataset and ascertained the AUROC in both the UHB and CovidCollab datasets. Based on model performance observed in the initial model derivation and in the interest of clinical utility, we used only categorical rather than continuous numerical variables, with imputed missing values (imputed prior to categorisation). To verify that predictors behaved similarly, we compared logistic coefficients from UHB to the same models fitted on the CovidCollab dataset. To account for sensitivity to missing values, we performed training and testing five times on fivefold multiple imputed datasets for both UHB and CovidCollab.

### External validation of ISARIC 4C score

A logistic regression using the 4C score was performed in the UHB dataset (following the same modelling methods used in the original ISARIC study). Model performance was assessed by calculating the AUROC and plotting calibration curves.

### Sensitivity analyses

Most patient records had some missing variables; we therefore performed a complete case analysis where we refitted the best forward stepwise selection model derived using the full set of UHB variables to complete case data, then data with ≤1, 2, 5 and 10 missing values, imputing missing values in the same way as previously mentioned, and examined AUROCs and logistic coefficients for stability.

In addition, we performed sensitivity analyses (1) within male and female strata by assessing performance (AUROC) of the final models in male and female patients separately; and (2) within age strata by assessing model performance in patients aged ≤60 and>60 years separately.

### Time series analysis

The UHB regression models used baseline measurement data collected on admission; where not available at admission, we accepted values up to 72 hours after admission. To investigate fine-grained temporal effects of data acquisition, we produced a series of separate logistic regression models using data collected at different time windows from within 24 hours of admission up to within 7 days of admission, in 1-day increments, for the mortality outcome. Each dataset included only those patients eligible at the end of the window (not dead or discharged). This created eight different sets of predictors, including baseline variables of age, gender, symptoms and the time-sensitive variables of the latest physiological and laboratory measurements available.

For missing data, data were carried forward from the first observation (last observation carried forward (LOCF)) and fivefold multiple imputation was performed for missing data after LOCF was done, within each separate time-window dataset. Each model was trained and tested in fivefold cross validation, within each imputation, and AUROCs averaged using Rubin’s rule. We compared the AUROCs for each of the eight models for predicting 28-day mortality from the time of admission and compared the logistic coefficients for the models. For additional insight into possible effects of changing measurements, we produced an additional logistic model for 28-day mortality to time-sensitive data collected within 4 days of admission, augmented with predictors indicating an increase or decrease in the category of each time-sensitive predictor relative to the reference category from 0 to 4 days, for example, whether temperature had crossed from below to above 37.8°C in that period.

### Patient and public involvement

We engaged with members of the PIONEER patient and public involvement group during development of the study protocol. We will further engage with this group, as well as other local and national patient and public involvement groups, in order to discuss dissemination of the findings and the best way to communicate these to patients and the public. We also consulted with several secondary care clinicians before and during the study to ensure that the tools developed meet the needs of clinicians. We have engaged with local NHS trusts to ensure that the algorithms developed are implemented/tested in a hospital setting.

## Results

### Derivation cohort characteristics

A total of 1040 participants with COVID-19 admitted to UHB were included in the derivation cohort. A total of 288 (28%) died within 28 days of admission and 183 (18%) were admitted to ITU. Baseline characteristics are presented in table 1 (stratified by mortality outcome) and online supplemental table 1 (stratified by ITU admission). The mean (SD) age of participants was 68.2 (17.7) years; 57% (589) were male; and almost 90% had at least one comorbidity.

**Table 1:** Baseline characteristics of participants admitted with COVID-19 in the derivation (UHB) and validation (CovidCollab) datasets

	Development cohort (UHB)			External validation cohort (CovidCollab)		
	Total	Alive	Died	Total	Alive	Died
(A) Demographic characteristics, comorbidities and symptoms						
N	1040	752	288	6099	4431	1668
Age category (years), n (%)						
<30	35 (3.4)	35 (4.7)	0 (0.0)	125 (2.0)	122 (2.8)	3 (0.2)
30–39	42 (4.0)	39 (5.2)	3 (1.0)	270 (4.4)	257 (5.8)	13 (0.8)
40–49	91 (8.8)	87 (11.6)	4 (1.4)	497 (8.1)	459 (10.4)	38 (2.3)
50–59	146 (14.0)	123 (16.4)	23 (8.0)	793 (13.0)	707 (16.0)	86 (5.2)
60–69	181 (17.4)	143 (19.0)	38 (13.2)	944 (15.5)	736 (16.6)	208 (12.5)
70–79	220 (21.2)	147 (19.5)	73 (25.3)	1325 (21.7)	891 (20.1)	434 (26.0)
80–89	214 (20.6)	124 (16.5)	90 (31.2)	1571 (25.8)	936 (21.1)	635 (38.1)
≥90	111 (10.7)	54 (7.2)	57 (19.8)	574 (9.4)	323 (7.3)	251 (15.0)
Gender (male), n (%)	589 (56.6)	423 (56.2)	166 (57.6)	3361 (55.1)	2342 (52.9)	1019 (61.1)
Ethnicity, n (%)				Not available		
White	590 (56.7)	406 (54.0)	184 (63.9)			
South Asian	127 (12.2)	95 (12.6)	32 (11.1)			
Black	68 (6.5)	46 (6.1)	22 (7.6)			
Other	255 (24.5)	205 (27.3)	50 (17.4)			
Comorbidities, n (%)						
Dementia	373 (35.9)	249 (33.1)	124 (43.1)	934 (15.3)	539 (12.2)	395 (23.7)
Cancer	135 (13.0)	90 (12.0)	45 (15.6)	649 (10.6)	409 (9.2)	240 (14.4)
Asthma	165 (15.9)	135 (18.0)	30 (10.4)	1579 (25.9)*	1125 (25.4)*	454 (27.2)*
Chronic obstructive pulmonary disease	283 (27.2)	211 (28.1)	72 (25.0)			
Sleep apnoea	49 (4.7)	36 (4.8)	13 (4.5)			
Cardiovascular disease	567 (54.5)	366 (48.7)	201 (69.8)	3033 (49.7)	1977 (44.6)	1056 (63.3)
Hypertension	661 (63.6)	449 (59.7)	212 (73.6)	Not available		
Diabetes without complications	258 (24.8)	181 (24.1)	77 (26.7)	1794 (29.4)	1229 (27.7)	565 (33.9)
Diabetes with complications	112 (10.8)	76 (10.1)	36 (12.5)	Not available		
Peptic ulcer	29 (2.8)	25 (3.3)	4 (1.4)	Not available		
Liver disease	71 (6.8)	53 (7.0)	18 (6.2)	Not available		
Rheumatic/inflammatory disease	51 (4.9)	36 (4.8)	15 (5.2)	Not available		
Thyroid disorder	107 (10.3)	75 (10.0)	32 (11.1)	Not available		
ISARIC comorbidity score				Not applicable		
0	111 (10.7)	94 (12.5)	17 (5.9)			
1	234 (22.5)	175 (23.3)	59 (20.5)			
≥2	695 (66.8)	483 (64.2)	212 (73.6)			
Symptoms, n (%)						
Breathlessness	559 (59.2)	392 (56.6)	167 (66.3)	Not available		
Chest pain	39 (4.1)	33 (4.8)	6 (2.4)	Not available		
Cough	538 (57.0)	398 (57.5)	140 (55.6)	4259 (69.8)	3110 (70.2)	1149 (68.9)
Fever	465 (49.3)	339 (49.0)	126 (50.0)	3212 (52.7)	2394 (54.0)	818 (49.0)
Headache	42 (4.4)	37 (5.3)	5 (2.0)	Not available		
Malaise	186 (19.7)	147 (21.2)	39 (15.5)	Not available		
New-onset diarrhoea or vomiting	56 (5.9)	49 (7.1)	7 (2.8)	Not available		
Sputum	84 (8.9)	53 (7.7)	31 (12.3)	Not available		
Delirium	80 (8.5)	41 (5.9)	39 (15.5)	1160 (20.1)	699 (16.7)	461 (28.8)
Outcomes						
Died within 28 days of admission	288 (27.7)	0 (0.0)	288 (100.0)	1668 (27.3)	0 (0.0)	1668 (100.0)
ITU admission within 28 days	183 (17.6)	132 (17.6)	51 (17.7)	722 (11.8)	477 (10.8)	245 (14.7)
(B) Physiological measures and scores						
BMI category, kg/m^2^						
Underweight (<18.5)	27 (2.6)	16 (2.1)	11 (3.8)	155 (2.5)	112 (2.5)	43 (2.6)
Normal weight (18.5–24.9)	273 (26.2)	179 (23.8)	94 (32.6)	1284 (21.1)	940 (21.2)	344 (20.6)
Overweight (25–29.9)	341 (32.8)	252 (33.5)	89 (30.9)	1247 (20.4)	999 (22.5)	248 (14.9)
Obese (≥30)	366 (35.2)	280 (37.2)	86 (29.9)	1180 (19.3)	940 (21.2)	240 (14.4)
Missing	33 (3.2)	25 (3.3)	8 (2.8)	2233 (36.6)	1440 (32.5)	793 (47.5)
Systolic blood pressure (mm Hg), n (%)						
<140	688 (66.2)	503 (66.9)	185 (64.2)	4051 (66.4)	2936 (66.3)	1115 (66.8)
≥140	340 (32.7)	249 (33.1)	91 (31.6)	1926 (31.6)	1414 (31.9)	512 (30.7)
Missing	12 (1.2)	0 (0.0)	12 (4.2)	122 (2.0)	81 (1.8)	41 (2.5)
Diastolic blood pressure (mm Hg), n (%)						
<90	870 (83.7)	636 (84.6)	234 (81.2)	5075 (83.2)	3654 (82.5)	1421 (85.2)
≥90	158 (15.2)	116 (15.4)	42 (14.6)	910 (14.9)	701 (15.8)	209 (12.5)
Missing	12 (1.2)	0 (0.0)	12 (4.2)	114 (1.9)	76 (1.7)	38 (2.3)
Temperature (degrees Celsius), n (%)						
<37.8	851 (81.8)	619 (82.3)	232 (80.6)	4164 (68.3)	3025 (68.3)	1139 (68.3)
≥37.8	187 (18.0)	133 (17.7)	54 (18.8)	1805 (29.6)	1316 (29.7)	489 (29.3)
Missing	2 (0.2)	0 (0.0)	2 (0.7)	130 (2.1)	90 (2.0)	40 (2.4)
Heart rate category (beats/min), n (%)						
<80	288 (27.7)	211 (28.1)	77 (26.7)	1654 (27.1)	1190 (26.9)	464 (27.8)
80–99	441 (42.4)	326 (43.4)	115 (39.9)	2400 (39.4)	1794 (40.5)	606 (36.3)
≥100	309 (29.7)	215 (28.6)	94 (32.6)	1935 (31.7)	1370 (30.9)	565 (33.9)
Missing	2 (0.2)	0 (0.0)	2 (0.7)	110 (1.8)	77 (1.7)	33 (2.0)
Respirations (breaths/min), n (%)						
<20	450 (43.3)	363 (48.3)	87 (30.2)	2249 (36.9)	1769 (39.9)	480 (28.8)
≥20	573 (55.1)	389 (51.7)	184 (63.9)	3659 (60.0)	2522 (56.9)	1137 (68.2)
Missing	17 (1.6)	0 (0.0)	17 (5.9)	191 (3.1)	140 (3.2)	51 (3.1)
Oxygen saturation (%), n (%)						
<80	9 (0.9)	4 (0.5)	5 (1.7)	158 (2.6)	71 (1.6)	87 (5.2)
80–88	47 (4.5)	17 (2.3)	30 (10.4)	443 (7.3)	230 (5.2)	213 (12.8)
89–93	173 (16.6)	110 (14.6)	63 (21.9)	1108 (18.2)	775 (17.5)	333 (20.0)
≥94	809 (77.8)	621 (82.6)	188 (65.3)	4281 (70.2)	3284 (74.1)	997 (59.8)
Missing	2 (0.2)	0 (0.0)	2 (0.7)	109 (1.8)	71 (1.6)	38 (2.3)
Partial pressure of CO_2_ (kPa), n (%)						
<4.67	184 (17.7)	125 (16.6)	59 (20.5)	947 (15.5)	627 (14.2)	320 (19.2)
4.67–6.3	380 (36.5)	278 (37.0)	102 (35.4)	650 (10.7)	467 (10.5)	183 (11.0)
≥6.4	176 (16.9)	124 (16.5)	52 (18.1)	214 (3.5)	128 (2.9)	86 (5.2)
Missing	300 (28.8)	225 (29.9)	75 (26.0)	4288 (70.3)	3209 (72.4)	1079 (64.7)
Portable oxygen concentrator fraction of inspired oxygen (%), n (%)						
≤0.28	629 (60.5)	458 (60.9)	171 (59.4)	3518 (57.7)	2730 (61.6)	788 (47.2)
0.28–0.49	56 (5.4)	35 (4.7)	21 (7.3)	1132 (18.6)	794 (17.9)	338 (20.3)
≥0.5	80 (7.7)	51 (6.8)	29 (10.1)	1003 (16.4)	541 (12.2)	462 (27.7)
Missing	275 (26.4)	208 (27.7)	67 (23.3)	446 (7.3)	366 (8.3)	80 (4.8)
Chest X-ray						
Clear/unchanged	210 (20.2)	161 (21.4)	49 (17.0)	1604 (26.3)	1225 (27.6)	379 (22.7)
Local consolidation	235 (22.6)	169 (22.5)	66 (22.9)	3226 (52.9)	2313 (52.2)	913 (54.7)
Ground-glass opacity/bilateral infiltrates	393 (37.8)	273 (36.3)	120 (41.7)	637 (10.4)	402 (9.1)	235 (14.1)
Other/no firm diagnosis	99 (9.5)	65 (8.6)	34 (11.8)	–	–	–
None performed/missing	103 (9.9)	84 (11.2)	19 (6.6)	632 (10.4)	491 (11.1)	141 (8.5)
Frailty score, n (%)						
1–3	376 (36.2)	321 (42.7)	55 (19.1)	1451 (23.8)	1326 (29.9)	125 (7.5)
4–6	277 (26.6)	179 (23.8)	98 (34.0)	2079 (34.1)	1539 (34.7)	540 (32.4)
7–9	119 (11.4)	55 (7.3)	64 (22.2)	1911 (31.3)	1146 (25.9)	765 (45.9)
Missing	268 (25.8)	197 (26.2)	71 (24.7)	658 (10.8)	420 (9.5)	238 (14.3)
Glasgow Coma Scale score, n (%)						
<15	274 (26.3)	178 (23.7)	96 (33.3)	1314 (21.5)	744 (16.8)	570 (34.2)
15	222 (21.3)	186 (24.7)	36 (12.5)	4250 (69.7)	3366 (76.0)	884 (53.0)
Missing	544 (52.3)	388 (51.6)	156 (54.2)	535 (8.8)	321 (7.2)	214 (12.8)
(C) Laboratory test results						
eGFR (mL/min), n (%)						
<30 (stage 4 or above)	200 (19.2)	119 (15.8)	81 (28.1)	693 (11.4)	364 (8.2)	329 (19.7)
30–59 (stage 3)	216 (20.8)	141 (18.8)	75 (26.0)	1362 (22.3)	846 (19.1)	516 (30.9)
60–89 (stage 2)	317 (30.5)	246 (32.7)	71 (24.7)	1825 (29.9)	1393 (31.4)	432 (25.9)
>90 (normal or high)	259 (24.9)	215 (28.6)	44 (15.3)	1818 (29.8)	1531 (34.6)	287 (17.2)
Missing	48 (4.6)	31 (4.1)	17 (5.9)	401 (6.6)	297 (6.7)	104 (6.2)
pH, n (%)						
<7.30	64 (6.2)	39 (5.2)	25 (8.7)	416 (6.8)	234 (5.3)	182 (10.9)
7.30–7.34	88 (8.5)	64 (8.5)	24 (8.3)	530 (8.7)	351 (7.9)	179 (10.7)
7.35–7.44	429 (41.2)	313 (41.6)	116 (40.3)	2300 (37.7)	1643 (37.1)	657 (39.4)
≥7.45	152 (14.6)	107 (14.2)	45 (15.6)	960 (15.7)	725 (16.4)	235 (14.1)
Missing	307 (29.5)	229 (30.5)	78 (27.1)	1893 (31.0)	1478 (33.4)	415 (24.9)
Base excess (mmol/L), n (%)						
<−2	202 (19.4)	125 (16.6)	77 (26.7)	981 (16.1)	560 (12.6)	421 (25.2)
−2 to 2	349 (33.6)	262 (34.8)	87 (30.2)	1996 (32.7)	1491 (33.6)	505 (30.3)
>2	182 (17.5)	136 (18.1)	46 (16.0)	1077 (17.7)	801 (18.1)	276 (16.5)
Missing	307 (29.5)	229 (30.5)	78 (27.1)	2045 (33.5)	1579 (35.6)	466 (27.9)
Anion gap (mmol/L), n (%)				Not available		
6–15	89 (8.6)	62 (8.2)	27 (9.4)			
≥16	579 (55.7)	409 (54.4)	170 (59.0)			
Missing	372 (35.8)	281 (37.4)	91 (31.6)			
White blood cell count (10^9^ /L), n (%)				Not available		
<3.9	79 (7.6)	69 (9.2)	10 (3.5)			
3.9–10.8	696 (66.9)	518 (68.9)	178 (61.8)			
≥10.9	215 (20.7)	135 (18.0)	80 (27.8)			
Missing	50 (4.8)	30 (4.0)	20 (6.9)			
Platelets (10^9^ /L), n (%)				Not available		
<150	179 (17.2)	121 (16.1)	58 (20.1)			
150–399	728 (70.0)	538 (71.5)	190 (66.0)			
≥400	80 (7.7)	62 (8.2)	18 (6.2)			
Missing	53 (5.1)	31 (4.1)	22 (7.6)			
Lymphocytes (10^9^ /L), n (%)						
<1.5	801 (77.0)	572 (76.1)	229 (79.5)	4684 (76.8)	3349 (75.6)	1335 (80.0)
≥1.5	195 (18.8)	154 (20.5)	41 (14.2)	1183 (19.4)	929 (21.0)	254 (15.2)
Missing	44 (4.2)	26 (3.5)	18 (6.2)	232 (3.8)	153 (3.5)	79 (4.7)
Neutrophil:lymphocyte ratio, n (%)						
<2.21	94 (9.0)	82 (10.9)	12 (4.2)	600 (9.8)	509 (11.5)	91 (5.5)
2.21–4.82	282 (27.1)	223 (29.7)	59 (20.5)	1635 (26.8)	1341 (30.3)	294 (17.6)
>4.82	620 (59.6)	421 (56.0)	199 (69.1)	3387 (55.5)	2265 (51.1)	1122 (67.3)
Missing	44 (4.2)	26 (3.5)	18 (6.2)	477 (7.8)	316 (7.1)	161 (9.7)
Mean corpuscular volume (fL), n (%)				Not available		
<80	91 (8.8)	69 (9.2)	22 (7.6)			
80–95	782 (75.2)	578 (76.9)	204 (70.8)			
≥96	123 (11.8)	79 (10.5)	44 (15.3)			
Missing	44 (4.2)	26 (3.5)	18 (6.2)			
Red cell distribution width (%), n (%)				Not available		
<11.5	13 (1.2)	11 (1.5)	2 (0.7)			
11.5–15.4	742 (71.3)	555 (73.8)	187 (64.9)			
≥15.5	240 (23.1)	160 (21.3)	80 (27.8)			
Missing	45 (4.3)	26 (3.5)	19 (6.6)			
Monocytes (10^9^ /L), n (%)				Not available		
<0.2	71 (6.8)	51 (6.8)	20 (6.9)			
0.2–0.8	731 (70.3)	539 (71.7)	192 (66.7)			
>0.8	194 (18.7)	136 (18.1)	58 (20.1)			
Missing	44 (4.2)	26 (3.5)	18 (6.2)			
Eosinophils (10^9^ /L), n (%)				Not available		
≤0.4	979 (94.1)	710 (94.4)	269 (93.4)			
>0.4	17 (1.6)	16 (2.1)	1 (0.3)			
Missing	44 (4.2)	26 (3.5)	18 (6.2)			
Haemoglobin (g/L), n (%)						
<115	310 (29.8)	215 (28.6)	95 (33.0)	1454 (23.8)	975 (22.0)	479 (28.7)
115–153	582 (56.0)	434 (57.7)	148 (51.4)	3718 (61.0)	2783 (62.8)	935 (56.1)
≥154	98 (9.4)	73 (9.7)	25 (8.7)	557 (9.1)	413 (9.3)	144 (8.6)
Missing	50 (4.8)	30 (4.0)	20 (6.9)	370 (6.1)	260 (5.9)	110 (6.6)
Glucose (mmol/L), n (%)				Not available		
<7.8	585 (56.2)	441 (58.6)	144 (50.0)			
7.8–8.4	76 (7.3)	47 (6.2)	29 (10.1)			
≥8.5	295 (28.4)	201 (26.7)	94 (32.6)			
Missing	84 (8.1)	63 (8.4)	21 (7.3)			
Bicarbonate (mmol/L), n (%)						
<22	184 (17.7)	118 (15.7)	66 (22.9)	996 (16.3)	608 (13.7)	388 (23.3)
22–28	451 (43.4)	339 (45.1)	112 (38.9)	2704 (44.3)	1981 (44.7)	723 (43.3)
≥29	98 (9.4)	66 (8.8)	32 (11.1)	437 (7.2)	320 (7.2)	117 (7.0)
Missing	307 (29.5)	229 (30.5)	78 (27.1)	1962 (32.2)	1522 (34.3)	440 (26.4)
C reactive protein (mg/L), n (%)						
<10	84 (8.1)	76 (10.1)	8 (2.8)	690 (11.3)	616 (13.9)	74 (4.4)
10–99	406 (39.0)	321 (42.7)	85 (29.5)	2735 (44.8)	2101 (47.4)	634 (38.0)
≥100	483 (46.4)	314 (41.8)	169 (58.7)	2213 (36.3)	1381 (31.2)	832 (49.9)
Missing	67 (6.4)	41 (5.5)	26 (9.0)	461 (7.6)	333 (7.5)	128 (7.7)
Albumin (g/L), n (%)				Not available		
<25	189 (18.2)	123 (16.4)	66 (22.9)			
25–34	569 (54.7)	408 (54.3)	161 (55.9)			
≥35	214 (20.6)	176 (23.4)	38 (13.2)			
Missing	68 (6.5)	45 (6.0)	23 (8.0)			
Bilirubin (μmol/L), n (%)				Not available		
<21	822 (79.0)	604 (80.3)	218 (75.7)			
≥21	151 (14.5)	104 (13.8)	47 (16.3)			
Missing	67 (6.4)	44 (5.9)	23 (8.0)			
Alanine aminotransferase (U/L), n (%)						
<55	837 (80.5)	601 (79.9)	236 (81.9)	4126 (67.7)	2986 (67.4)	1140 (68.3)
≥55	134 (12.9)	106 (14.1)	28 (9.7)	777 (12.7)	559 (12.6)	218 (13.1)
Missing	69 (6.6)	45 (6.0)	24 (8.3)	1196 (19.6)	886 (20.0)	310 (18.6)
Alkaline phosphatase (U/L), n (%)				Not available		
<130	814 (78.3)	605 (80.5)	209 (72.6)			
≥130	159 (15.3)	103 (13.7)	56 (19.4)			
Missing	67 (6.4)	44 (5.9)	23 (8.0)			
Urea (mmol/L), n (%)						
<7.8	567 (54.5)	466 (62.0)	101 (35.1)	2585 (42.4)	2122 (47.9)	463 (27.8)
≥7.8	429 (41.2)	259 (34.4)	170 (59.0)	2409 (39.5)	1399 (31.6)	1010 (60.6)
Missing	44 (4.2)	27 (3.6)	17 (5.9)	1105 (18.1)	910 (20.5)	195 (11.7)
Potassium (mmol/L), n (%)				Not available		
2.5–5.2	868 (83.5)	644 (85.6)	224 (77.8)			
≥5.3	49 (4.7)	29 (3.9)	20 (6.9)			
Missing	123 (11.8)	79 (10.5)	44 (15.3)			
Sodium (mmol/L), n (%)				Not available		
<133	146 (14.0)	105 (14.0)	41 (14.2)			
133–144	746 (71.7)	567 (75.4)	179 (62.2)			
≥145	104 (10.0)	53 (7.0)	51 (17.7)			
Missing	44 (4.2)	27 (3.6)	17 (5.9)			
Corrected calcium (mmol/L), n (%)				Not available		
<2.2	142 (13.7)	99 (13.2)	43 (14.9)			
2.2–2.5	767 (73.8)	577 (76.7)	190 (66.0)			
≥2.6	38 (3.7)	17 (2.3)	21 (7.3)			
Missing	93 (8.9)	59 (7.8)	34 (11.8)			
Lactate (U/L), n (%)						
≤2.2	490 (47.1)	362 (48.1)	128 (44.4)	3091 (50.7)	2327 (52.5)	764 (45.8)
>2.2	192 (18.5)	121 (16.1)	71 (24.7)	996 (16.3)	558 (12.6)	438 (26.3)
Missing	358 (34.4)	269 (35.8)	89 (30.9)	2012 (33.0)	1546 (34.9)	466 (27.9)
Haematocrit (L/L), n (%)				Not available		
<0.5	963 (92.6)	707 (94.0)	256 (88.9)			
≥0.5	33 (3.2)	19 (2.5)	14 (4.9)			
Missing	44 (4.2)	26 (3.5)	18 (6.2)			

*In CovidCollab, asthma, chronic obstructive pulmonary disease and sleep apnoea were combined as ‘respiratory diseases’. Data on the individual conditions were not available; therefore, the n (%) given is for all respiratory diseases.

BMI, body mass index; eGFR, estimated glomerular filtration rate; ISARIC, International Severe Acute Respiratory and Emerging Infection Consortium; ITU, intensive therapy unit; UHB, University Hospitals Birmingham.

### Candidate predictors

After exclusion of seven candidate predictors with >40% missing data (D-dimer, ferritin, high-sensitivity troponin, fibrinogen, lactate dehydrogenase, vitamin D and haemoglobin A1c), 63 predictors were considered for inclusion in the models:

Demographic characteristics: age, gender and ethnicity.

Symptoms (binary, presence or absence of symptom at admission): breathlessness, chest pain, cough, fever, headache, malaise, new-onset diarrhoea or vomiting, sputum and delirium.

Physiological measures and vital signs: body mass index (BMI, kg/m^2^), systolic blood pressure (mm Hg), diastolic blood pressure (mm Hg), temperature (degrees Celsius), heart rate (beats/min), respiratory rate (breaths/min), oxygen saturation (%), partial pressure of CO_2_ (kPa) and portable oxygen concentrator fraction of inspired oxygen (FiO_2_, %).

Imaging: chest X-ray finding (categorised as clear/unchanged, local consolidation, ground-glass opacity/bilateral infiltrates, other/no firm diagnosis, none performed/missing).

Scores: frailty score (Rockwood Clinical Frailty Scale)^14^; Glasgow Coma Scale score^15 16^;

laboratory test results: estimated glomerular filtration rate (eGFR, ml/min), pH (%), base excess (mmol/L), anion gap (mmol/L), white blood cell (WBC) count (10^9^/L), platelets (10^9^/L), lymphocytes (10^9^/L), neutrophil:lymphocyte ratio, mean corpuscular volume (fL), red cell distribution width (%), monocytes (10^9^/L), eosinophils (10^9^/L), haemoglobin (g/L), glucose (mmol/L), bicarbonate (mmol/L), C reactive protein (mg/L), albumin (g/L), bilirubin (μmol/L), alanine aminotransferase (U/l), alkaline phosphatase (U/l), urea (mmol/l), potassium (mmol/l), sodium (mmol/l), corrected calcium (mmol/l), lactate (U/l) and haematocrit (l/l);

Comorbidities (binary, presence or absence of record in discharge ICD-10 codes): dementia, cancer, asthma, chronic obstructive pulmonary disease, sleep apnoea, cardiovascular disease, hypertension, diabetes without complications, diabetes with complications, peptic ulcer, liver disease, rheumatic/inflammatory disease, thyroid disorder.

### Mortality outcome (28 days): UHB model and predictive performance

Area under the ROC curve values for each of the logistic, LASSO and GBM models, treating continuous variables in one of three ways (as continuous variables with imputed missing values; as clinically meaningful categorical variables with imputed missing values; and as categorical variables with missing categories), are presented in online supplemental table 2.

The final model selected was a logistic regression using stepwise selection of variables with categorisation of continuous variables (with imputed missing values). The final 18 categorical predictors included in the model were: age, breathlessness, sputum, systolic blood pressure, temperature, respiratory rate, oxygen saturation, FiO_2_, alkaline phosphatase, C-reactive protein, corrected calcium, eosinophils, glucose, pH, urea, WBC count, platelets and frailty score.

**Figure 1:**
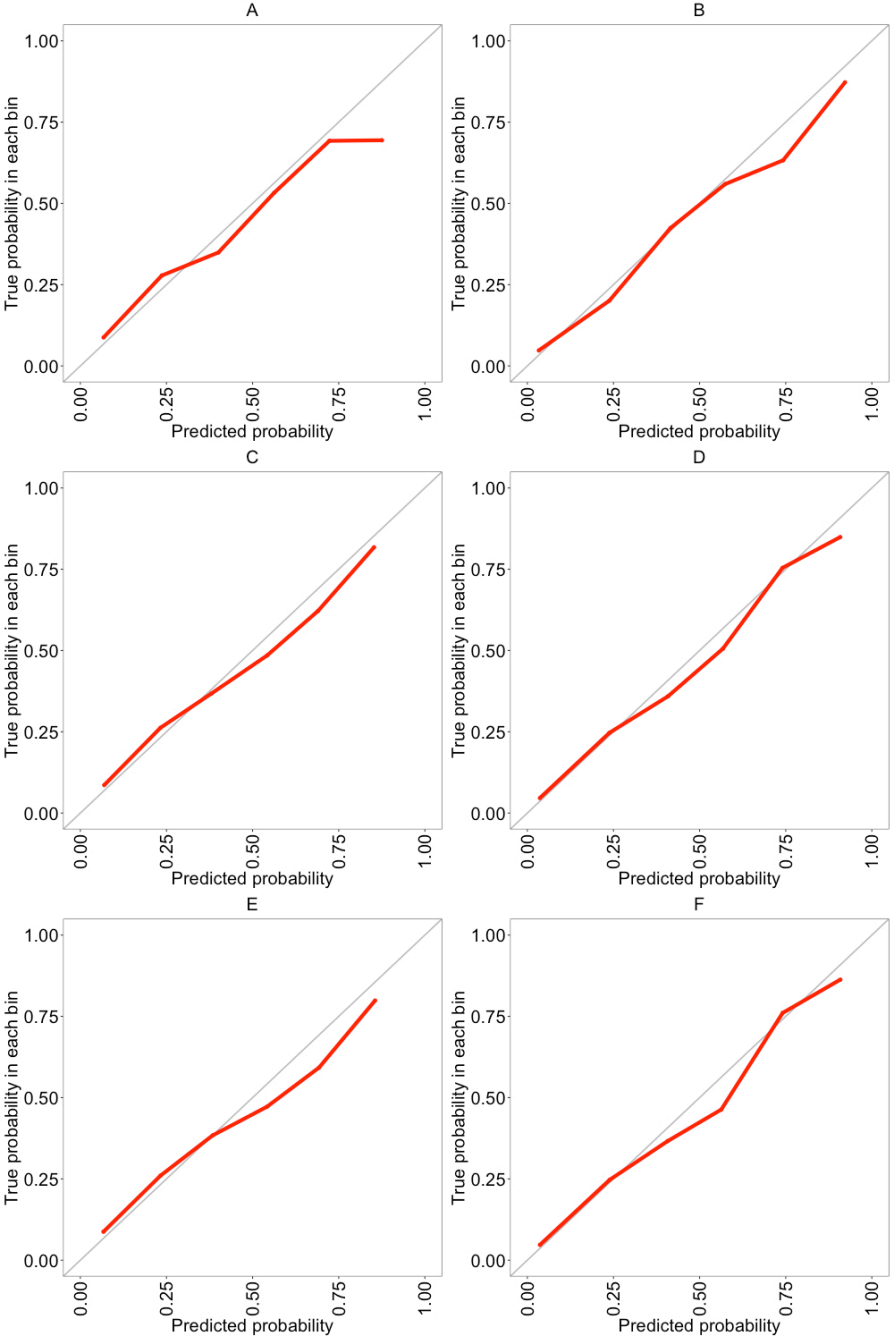
Calibration plots (observed probability (y-axis) against predicted probability (x-axis)): (A) UHB derivation dataset for mortality outcome, (B) UHB derivation dataset for ITU admission outcome, (C) UHB-R derivation/train dataset reduced model for mortality outcome, (D) UHB-R derivation/train dataset reduced model for ITU admission outcome, (E) CovidCollab external validation dataset reduced model for mortality outcome, (F) CovidCollab external validation dataset reduced model for ITU admission outcome. ITU, intensive therapy unit; UHB, University Hospitals Birmingham; UHB-R, University Hospitals Birmingham reduced.

AUROC for the UHB cross-validated model was 0.779 (95 % CI 0.744 to 0.813) (table 2). At a 20% predicted probability of mortality, sensitivity was 83% (95% CI 81% to 85%); specificity was 58% (95% CI 55% to 61%); positive predictive value was 43% (95% CI 41% to 46%); and negative predictive was 90% (95% CI 88% to 91%) (table 3A). Calibration was very good at low to medium predicted probabilities but was poorer at very high predicted probabilities; a calibration plot is shown in figure 1A; the calibration slope was 0.79 (95% CI 0.64 to 0.94) (table 2). Model coefficients (and model equation) are presented in online supplemental table 3.

**Table 2:** AUROCs, calibration slopes and calibration intercepts for models developed in UHB data (full (UHB) and reduced (UHB-R) datasets) and externally validated in CovidCollab data, and for external validation of the ISARIC 4C score

Dataset	Outcome	AUROC (95% CI)	Calibration (95% CI)	
			Slope	Intercept
Model development*				
UHB	Mortality	0.779 (0.744 to 0.813)	0.79 (0.64 to 0.94)	0.06 (−0.02 to 0.14)
UHB	ITU admission	0.893 (0.864 to 0.922)	0.91 (0.80 to 1.01)	0.01 (−0.05 to 0.07)
UHB-R†	Mortality	0.791 (0.761 to 0.822)	0.89 (0.81 to 0.97)	0.03 (−0.01 to 0.07)
UHB-R†	ITU admission	0.906 (0.883 to 0.929)	0.94 (0.84 to 1.04)	0.00 (−0.05 to 0.06)
External validation of new model*				
CovidCollab	Mortality	0.767 (0.754 to 0.780)	0.85 (0.75 to 0.94)	0.04 (−0.01 to 0.09)
CovidCollab	ITU admission	0.811 (0.795 to 0.828)	0.95 (0.82 to 1.08)	0.00 (−0.07 to 0.07)
External validation of ISARIC 4C score in UHB data				
UHB—4C score	Mortality	0.753 (0.720 to 0.785)	0.99 (0.85 to 1.12)	0.00 (−0.06 to 0.06)

*Models derived using logistic regression with stepwise selection of candidate predictors and categorisation of continuous variables into clinically meaningful categories (after imputing missing data).

†Not all variables included in the full UHB model were available in the CovidCollab dataset. Therefore, revised (reduced) models were developed in UHB data using a subset of the candidate predictors common to both the UHB and CovidCollab datasets (UHB-R), and these were then externally validated in the CovidCollab dataset.

AUROC, area under the receiver operating characteristic curve; ISARIC, International Severe Acute Respiratory and Emerging Infection Consortium; UHB, University Hospitals Birmingham; UHB-R, University Hospitals Birmingham reduced model.

**Table 3A:** Sensitivity, specificity, PPV and NPV for mortality at 28 days after admission (University Hospitals Birmingham derivation dataset)

Predicted probability (%)	TP	TN	FP	FN	Sensitivity (95% CI)	Specificity (95% CI)	PPV (95% CI)	NPV (95% CI)
10	270	275	477	18	0.938 (0.924 to 0.953)	0.365 (0.346 to 0.385)	0.362 (0.352 to 0.371)	0.939 (0.924 to 0.954)
20	238	437	315	50	0.828 (0.806 to 0.850)	0.581 (0.547 to 0.614)	0.431 (0.405 to 0.456)	0.898 (0.881 to 0.915)
30	194	542	210	94	0.672 (0.646 to 0.699)	0.721 (0.696 to 0.746)	0.480 (0.458 to 0.502)	0.852 (0.841 to 0.862)
40	156	622	130	132	0.540 (0.515 to 0.565)	0.827 (0.813 to 0.842)	0.545 (0.523 to 0.567)	0.825 (0.817 to 0.832)
50	116	678	74	172	0.404 (0.366 to 0.442)	0.902 (0.888 to 0.916)	0.613 (0.578 to 0.648)	0.798 (0.788 to 0.808)
60	82	712	40	206	0.284 (0.250 to 0.318)	0.947 (0.940 to 0.954)	0.673 (0.641 to 0.704)	0.775 (0.768 to 0.783)
70	50	731	21	238	0.174 (0.147 to 0.200)	0.972 (0.966 to 0.978)	0.706 (0.671 to 0.741)	0.754 (0.749 to 0.760)
80	24	741	11	264	0.083 (0.073 to 0.094)	0.986 (0.979 to 0.992)	0.691 (0.576 to 0.807)	0.737 (0.734 to 0.740)
90	8	750	2	280	0.026 (0.015 to 0.038)	0.997 (0.996 to 0.998)	0.772 (0.663 to 0.881)	0.728 (0.726 to 0.730)

FN, false negative; FP, false positive; NPV, negative predictive value; PPV, positive predictive value; TN, true negative; TP, true positive.

Addition of comorbidities to the candidate predictors included in the model did not improve performance of the model (online supplemental table 2). Since comorbidities are known to be under-reported during acute presentations,^6^ and they offered no improvement on model performance, models without comorbidities were preferred.

### ITU admission: UHB model and predictive performance

Area under the ROC curve values for each of the models performed are presented in online supplemental table 4.

The final model selected was a logistic regression using stepwise selection of variables with categorisation of continuous variables (with imputed missing values). The final 16 categorical predictors included in the model were: age, gender, fever, new onset diarrhoea or vomiting, heart rate, respiratory rate, FiO_2_, temperature, albumin, C-reactive protein, eGFR, pH, monocytes, WBC, frailty score, and Glasgow Coma Scale score.

AUROC was 0.893 (95% CI 0.864 to 0.922) (table 2). At a 20% predicted probability of ITU admission, sensitivity was 79% (95% CI 74 to 84), specificity was 83% (95% CI 81 to 84), positive predictive value was 49% (95% CI 46 to 52), and negative predictive was 95% (95% CI 94 to 96) (table 3B). Calibration was good; a calibration plot is shown in figure 1B, and the calibration slope was 0.91 (95% CI 0.80 to 1.01) (table 2). Model coefficients are presented in online supplemental table 5.

**Table 3B:** Sensitivity, specificity, PPV and NPV for intensive therapy unit admission within 28 days after admission (University Hospitals Birmingham derivation dataset)

Predicted probability (%)	TP	TN	FP	FN	Sensitivity (95% CI)	Specificity (95% CI)	PPV (95% CI)	NPV (95% CI)
10	161	617	240	22	0.878 (0.851 to 0.905)	0.720 (0.694 to 0.746)	0.401 (0.373 to 0.430)	0.965 (0.956 to 0.974)
20	144	709	148	39	0.789 (0.743 to 0.835)	0.827 (0.814 to 0.841)	0.494 (0.464 to 0.523)	0.948 (0.937 to 0.959)
30	129	771	86	54	0.707 (0.662 to 0.752)	0.900 (0.891 to 0.908)	0.601 (0.575 to 0.626)	0.935 (0.926 to 0.944)
40	115	797	60	68	0.631 (0.597 to 0.664)	0.930 (0.925 to 0.935)	0.659 (0.634 to 0.684)	0.922 (0.915 to 0.929)
50	97	817	40	86	0.532 (0.502 to 0.563)	0.954 (0.952 to 0.956)	0.711 (0.695 to 0.727)	0.905 (0.900 to 0.911)
60	81	831	26	102	0.445 (0.429 to 0.461)	0.970 (0.963 to 0.977)	0.760 (0.722 to 0.798)	0.891 (0.889 to 0.893)
70	67	841	16	116	0.367 (0.345 to 0.390)	0.982 (0.975 to 0.989)	0.812 (0.758 to 0.867)	0.879 (0.876 to 0.882)
80	55	848	9	128	0.303 (0.276 to 0.330)	0.989 (0.985 to 0.994)	0.860 (0.806 to 0.915)	0.869 (0.865 to 0.874)
90	35	854	3	148	0.190 (0.141 to 0.240)	0.997 (0.993 to 1)	0.924 (0.848 to 1)	0.852 (0.844 to 0.860)

FN, false negatives; FP, false positives; NPV, negative predictive value; PPV, positive predictive value; TN, true negatives; TP, true positives.

Addition of comorbidities to the predictors included in the model did not improve performance.

### Reduced UHB model and external validation in the CovidCollab dataset

A total of 6099 patients admitted with COVID-19 were included in the CovidCollab external validation dataset; 1668 (27%) died and 722 (12%) were admitted to ITU (table 1 and online supplemental table 1). Not all variables included in the UHB model derived previously were available in the CovidCollab dataset. Therefore, revised and reduced models were developed in UHB data using the subset of candidate predictors common to both the UHB and CovidCollab datasets (reduced UHB dataset, UHB-R), using logistic regression with stepwise selection, and these were then externally validated in the CovidCollab dataset.

The reduced set of 27 candidate predictors included demographic characteristics: age and gender; symptoms: cough, fever and delirium; physiological measures and vital signs: BMI, systolic blood pressure, diastolic blood pressure, heart rate, temperature, respiratory rate, oxygen saturation, FiO_2_ and chest X-ray; frailty score; Glasgow Coma Scale score; laboratory test results: eGFR, pH, base excess, lymphocytes, neutrophil:lymphocyte ratio, haemoglobin, bicarbonate, C reactive protein, alanine aminotransferase, urea and lactate.

#### Mortality (28 days)

For the 28-day mortality outcome, following stepwise selection, the final 10 categorical predictors (common to both datasets) included in the reduced logistic regression model were age, oxygen saturation, FiO_2_, respiratory rate, temperature, systolic blood pressure, C reactive protein, pH, urea and frailty score.

The selected predictors were a subset of those in the original UHB model derivation, but gave similar model performance. AUROC in the UHB-R dataset was 0.791 (95% CI 0.761 to 0.822), and AUROC in the CovidCollab external validation dataset was 0.767 (95% CI 0.754 to 0.780) (table 2). At a 20% predicted probability of mortality, in the UHB-R dataset, sensitivity was 86% (95% CI 85% to 88%); specificity was 54% (95% CI 51% to 57%); PPV was 42% (95% CI 40% to 43%); and NPV was 91% (95% CI 90% to 92%); in the CovidCollab dataset, sensitivity was 88% (95% CI 87% to 89%); specificity was 46% (95% CI 45% to 47%); PPV was 38% (95% CI 0.37% to 0.38%); and NPV was 91% (95% CI 91% to 92%) (table 4A).

**Table 4A:** Sensitivity, specificity, PPV and NPV for mortality at 28 days after admission for the reduced model (UHB derivation dataset and CovidCollab external validation dataset, using predictors common to both datasets)

Predicted probability	TP	TN	FP	FN	Sensitivity (95% CI)	Specificity (95% CI)	PPV (95% CI)	NPV (95% CI)
UHB-R derivation dataset (reduced model)								
10	278	249	503	10	0.964 (0.955 to 0.972)	0.332 (0.315 to 0.348)	0.356 (0.351 to 0.360)	0.960 (0.952 to 0.968)
20	248	406	346	40	0.862 (0.847 to 0.878)	0.539 (0.510 to 0.569)	0.418 (0.403 to 0.433)	0.911 (0.902 to 0.920)
30	202	536	216	86	0.703 (0.671 to 0.734)	0.713 (0.698 to 0.727)	0.484 (0.464 to 0.504)	0.862 (0.849 to 0.876)
40	153	635	117	135	0.532 (0.511 to 0.553)	0.844 (0.831 to 0.858)	0.567 (0.547 to 0.587)	0.825 (0.819 to 0.831)
50	104	686	66	184	0.360 (0.335 to 0.384)	0.913 (0.905 to 0.920)	0.612 (0.593 to 0.632)	0.788 (0.782 to 0.794)
60	66	723	29	222	0.228 (0.211 to 0.246)	0.962 (0.953 to 0.970)	0.696 (0.663 to 0.729)	0.765 (0.762 to 0.768)
70	35	744	8	253	0.122 (0.101 to 0.144)	0.990 (0.985 to 0.994)	0.819 (0.753 to 0.884)	0.746 (0.742 to 0.751)
80	14	750	2	274	0.050 (0.036 to 0.064)	0.997 (0.996 to 0.998)	0.868 (0.835 to 0.900)	0.733 (0.730 to 0.735)
90	3	752	0	285	0.010 (0 to 0.022)	0.999 (0.998 to 1)	0.900 (0.573 to 1)	0.725 (0.723 to 0.727)
CovidCollab external validation dataset								
10	1614	1218	3213	54	0.967 (0.962 to 0.973)	0.275 (0.260 to 0.290)	0.334 (0.330 to 0.338)	0.957 (0.951 to 0.963)
20	1471	2028	2403	197	0.882 (0.874 to 0.889)	0.458 (0.445 to 0.471)	0.380 (0.375 to 0.384)	0.911 (0.907 to 0.916)
30	1289	2721	1710	379	0.773 (0.763 to 0.782)	0.614 (0.602 to 0.626)	0.430 (0.425 to 0.435)	0.878 (0.874 to 0.881)
40	1059	3264	1167	609	0.635 (0.605 to 0.665)	0.737 (0.717 to 0.756)	0.476 (0.469 to 0.483)	0.843 (0.835 to 0.851)
50	850	3698	733	818	0.510 (0.470 to 0.549)	0.835 (0.817 to 0.852)	0.537 (0.523 to 0.551)	0.819 (0.810 to 0.828)
60	599	4025	406	1069	0.359 (0.305 to 0.414)	0.908 (0.890 to 0.927)	0.596 (0.578 to 0.615)	0.790 (0.779 to 0.801)
70	380	4222	209	1288	0.228 (0.186 to 0.270)	0.953 (0.940 to 0.965)	0.646 (0.626 to 0.666)	0.766 (0.759 to 0.774)
80	174	4355	76	1494	0.104 (0.077 to 0.132)	0.983 (0.978 to 0.987)	0.697 (0.671 to 0.722)	0.745 (0.740 to 0.750)
90	37	4414	17	1631	0.022 (0.006 to 0.039)	0.996 (0.995 to 0.998)	0.676 (0.572 to 0.780)	0.730 (0.727 to 0.733)

FN, false negative; FP, false positive; NPV, negative predictive value; PPV, positive predictive value; TN, true negative; TP, true positive; UHB, University Hospitals Birmingham; UHB-R, University Hospitals Birmingham reduced model.

Calibration was good for both derivation and external validation datasets; calibration plots are shown in figure 1C, D, and calibration slopes were 0.89 (95% CI 0.81 to 0.97) and 0.85 (95% CI 0.75 to 0.94) for the UHB-R and CovidCollab datasets, respectively (table 2). Model coefficients are presented in online supplemental table 6.

#### ITU admission

For the ITU admission outcome, the final 11 categorical predictors (common to both datasets) included in the reduced model were age, gender, fever, respiratory rate, FiO_2_, C reactive protein, eGFR, pH, neutrophil:lymphocyte ratio, frailty score and Glasgow Coma Scale score.

AUROC in the UHB-R dataset was 0.906 (95% CI 0.883 to 0.929), and in the CovidCollab dataset was 0.811 (95% CI 0.795 to 0.828) (table 2). At a 20% predicted probability of ITU admission, in the UHB-R dataset, sensitivity was 83% (95% CI 81% to 85%); specificity was 83% (95% CI 82% to 84%); PPV was 51% (95% CI 49% to 52%); and NPV was 96% (95% CI 95% to 96%); in the CovidCollab dataset, sensitivity was 64% (95% CI 62% to 67%); specificity was 80% (95% CI 79% to 82%); PPV was 30% (95% CI 29% to 32%); and NPV was 94% (95% CI 94% to 95%) (table 4B).

**Table 4B:** Sensitivity, specificity, PPV and NPV for intensive therapy unit admission within 28 days after admission in the reduced model (University Hospitals Birmingham derivation dataset and CovidCollab external validation dataset, using predictors common to both datasets)

Predicted probability	TP	TN	FP	FN	Sensitivity (95% CI)	Specificity (95% CI)	PPV (95% CI)	NPV (95% CI)
UHB-R derivation dataset (reduced model)								
10	165	590	267	18	0.904 (0.877 to 0.931)	0.689 (0.671 to 0.707)	0.383 (0.368 to 0.398)	0.971 (0.963 to 0.979)
20	152	709	148	31	0.831 (0.812 to 0.849)	0.827 (0.816 to 0.839)	0.507 (0.494 to 0.519)	0.958 (0.954 to 0.962)
30	132	765	92	51	0.723 (0.698 to 0.749)	0.893 (0.882 to 0.904)	0.591 (0.567 to 0.615)	0.938 (0.933 to 0.943)
40	112	803	54	71	0.613 (0.573 to 0.653)	0.937 (0.928 to 0.946)	0.675 (0.649 to 0.702)	0.919 (0.912 to 0.926)
50	92	829	28	91	0.505 (0.483 to 0.526)	0.967 (0.956 to 0.979)	0.768 (0.708 to 0.829)	0.901 (0.898 to 0.905)
60	77	841	16	106	0.419 (0.403 to 0.435)	0.981 (0.974 to 0.988)	0.828 (0.779 to 0.877)	0.888 (0.886 to 0.890)
70	67	847	10	116	0.365 (0.344 to 0.386)	0.988 (0.983 to 0.994)	0.870 (0.816 to 0.924)	0.879 (0.876 to 0.883)
80	46	851	6	137	0.251 (0.189 to 0.313)	0.993 (0.989 to 0.997)	0.889 (0.840 to 0.938)	0.861 (0.852 to 0.871)
90	20	855	2	163	0.108 (0.067 to 0.149)	0.997 (0.996 to 0.999)	0.898 (0.838 to 0.957)	0.840 (0.833 to 0.846)
CovidCollab external validation dataset								
10	574	3623	1754	148	0.794 (0.764 to 0.825)	0.674 (0.651 to 0.697)	0.247 (0.238 to 0.255)	0.961 (0.956 to 0.965)
20	465	4312	1065	257	0.644 (0.621 to 0.667)	0.802 (0.785 to 0.819)	0.304 (0.288 to 0.321)	0.944 (0.941 to 0.947)
30	377	4655	722	345	0.523 (0.497 to 0.549)	0.866 (0.847 to 0.885)	0.344 (0.320 to 0.367)	0.931 (0.929 to 0.933)
40	315	4881	496	407	0.437 (0.381 to 0.492)	0.908 (0.887 to 0.928)	0.389 (0.361 to 0.417)	0.923 (0.918 to 0.929)
50	250	5045	332	472	0.346 (0.258 to 0.434)	0.938 (0.920 to 0.956)	0.430 (0.413 to 0.447)	0.914 (0.905 to 0.923)
60	178	5168	209	544	0.247 (0.175 to 0.318)	0.961 (0.945 to 0.977)	0.462 (0.420 to 0.503)	0.905 (0.898 to 0.912)
70	113	5256	121	609	0.157 (0.105 to 0.208)	0.978 (0.967 to 0.988)	0.486 (0.439 to 0.534)	0.896 (0.891 to 0.901)
80	57	5319	58	665	0.079 (0.044 to 0.114)	0.989 (0.984 to 0.995)	0.500 (0.410 to 0.590)	0.889 (0.886 to 0.892)
90	18	5357	20	704	0.025 (0.006 to 0.044)	0.996 (0.993 to 1)	0.485 (0.367 to 0.603)	0.884 (0.882 to 0.886)

FN, false negatives; FP, false positives; NPV, negative predictive value; PPV, positive predictive value; TN, true negatives; TP, true positives; UHB-R, University Hospitals Birmingham reduced model.

Calibration was good for both derivation and external validation datasets; calibration plots are shown in figure 1E, F; calibration slopes were 0.94 (95% CI 0.84 to 1.04) and 0.95 (95% CI 0.82 to 1.08) for the UHB-R and CovidCollab datasets, respectively (table 2). Model coefficients are presented in online supplemental table 7.

### External validation of the ISARIC 4C score in the UHB dataset

The AUROC for the recently published ISARIC 4C score in the UHB dataset was 0.753 (95% CI 0.720 to 0.785). The calibration slope was 0.99 (95% CI 0.85 to 1.12) (table 2 and online supplemental figure 1).

10.1136/bmjopen-2021-049506.supp2 Supplementary data

It was not possible to externally validate the ISARIC 4C score in the CovidCollab dataset, as information on many of the comorbidities required to calculate the ISARIC comorbidity score was not available in the dataset.

### Sensitivity analyses

Analyses exploring different ways of handling missing data are reported in online supplemental appendix 2 and online supplemental figures 2 and 3.

10.1136/bmjopen-2021-049506.supp3 Supplementary data

10.1136/bmjopen-2021-049506.supp4 Supplementary data

#### Complete case analysis

Few patients in the dataset had complete data (n=224/1040, 22%); model performance in this patient subset was slightly poorer for the mortality outcome: AUROC 0.696 (95% CI 0.597 to 0.795) for mortality and 0.892 (95% CI 0.844 to 0.940) for ITU admission. Including patients with missing variables, with missing values imputed, improved model performance for predicting mortality; allowing even a single missing/imputed variable improved AUROC for mortality to 0.760 (95% CI 0.708 to 0.812) (online supplemental table 8).

#### Stratification by gender and age

When patients were stratified by gender, the reduced models predicting mortality and ITU still performed well: AUROCs for mortality were 0.775 (95% CI 0.726 to 0.823) for males and 0.755 (95% CI 0.706 to 0.804) for females, and those for ITU 0.897 (95% CI 0.856 to 0.937) for males and 0.873 (95% CI 0.833 to 0.913) for females (online supplemental table 9).

When patients were stratified by age, the models performed slightly better in patients aged >60 years (AUROC 0.778 (95% CI 0.722 to 0.834) and 0.897 (95% CI 0.864 to 0.930) for mortality and ITU admission, respectively) compared with those aged ≤60 years (AUROC 0.730 (95% CI 0.638 to 0.823) and 0.845 (95% CI 0.761 to 0.930) for mortality and ITU admission, respectively).

### Time series analysis

Online supplemental figure 4 shows variation in logistic regression coefficients for the candidate predictors from day of admission and up to 7 days later. The majority of coefficients remained relatively constant over time. However, several (not necessarily statistically significant) trends in the modification of effects over the week of admission on mortality were visible, such as a decrease over the week of the effect of obesity on mortality, elevated effect of eosinophils, and an increase over the week of the effect of elevated haemoglobin, elevated potassium and elevated oxygen saturation. Some of these might be depletion effects related to relatively high patient mortality in the first few days, for example, the apparent protective effect of obesity and high eosinophils.

10.1136/bmjopen-2021-049506.supp5 Supplementary data

## Discussion

Using routinely collected data for more than a thousand patients admitted with COVID-19 at a large UK hospital trust, we have developed and externally validated prognostic models for mortality and ITU admission. The models showed good discrimination and calibration. The candidate predictors explored included a clinically informed, wider range of demographics, clinical observations, symptoms, comorbidities, biomarkers and radiological investigations than those included in the derivation of existing prognostic scores or models.

If integrated into hospital electronic medical records systems, the model algorithms will provide a predicted probability of mortality or ITU admission within 28 days of hospital admission for each patient based on their individual data at, or close to, the time of admission, which will support clinicians’ decision making with regard to appropriate patient care pathways and triage. This information might also assist clinicians in explaining complex prognostic assessments and decisions to patients and their relatives, particularly at times when relatives are unable to see the patient and understand how unwell they are.

### Summary of results

The models developed using all 63 available candidate predictors from UHB performed well with an optimism-adjusted AUROC of 0.779 (95 % CI 0.744 to 0.813) for mortality within 28 days of admission and 0.893 (95% CI 0.864 to 0.922) for ITU admission.

Not all variables included in the UHB dataset are routinely collected at admission in other hospitals; therefore, reduced models using only variables common to both UHB and the CovidCollab external validation dataset were explored. Discrimination remained similar, with an AUROC of 0.791 (95% CI 0.761 to 0.822) for mortality and 0.906 (95% CI 0.883 to 0.929) for ITU admission in the UHB derivation dataset. These reduced models also performed well in the CovidCollab external validation dataset, with AUROCs of 0.767 (95% CI 0.754 to 0.780) and 0.811 (95% CI 0.795 to 0.828) for mortality and ITU admission, respectively. The models also performed well in gender-stratified and age-stratified patient subgroups.

Calibration of all models showed good agreement between observed and predicted probabilities, particularly at lower predicted probabilities in the range where the models would be of most clinical utility.

We found that addition of comorbidities to the model predictors did not improve overall model performance. This may be due to a correlation between presence of comorbidities and related physiological measurements and/or biomarkers which are already captured by the model.

### Comparison with existing literature

Two systematic reviews summarised the existing secondary care COVID-19 prognostic models or scores published until 31 May 2020.^1 17^ The majority of the reported models, along with several more recent ones,^18^ were derived in Chinese cohorts. Many of the models included in the reviews demonstrated high discriminatory performance; however, all pre-existing models when assessed using the PROBAST score were at high risk of bias. Furthermore, few models were externally validated in suitable cohorts. By deriving our model from routinely collected data, we were able to reduce the risk of bias in patient selection as well as predictor and outcome measurements. Additionally, in this study, we were able to externally validate models in a large global heterogeneous cohort.

More recently, the most notable secondary care prediction model advised for uptake in UK hospitals was derived from the ISARIC–WHO collaborating cohort and has been externally validated.^5 19^ Both the full and reduced UHB-derived models for mortality had slightly better discrimination than the ISARIC 4C score in the UHB data (AUROC 0.753, 95% CI 0.720 to 0.785 for 4C). This compares with an AUROC of 0.767 (95% CI 0.760 to 0.773) for the 4C score reported in the original ISARIC validation cohort.^5^ However, better performance may be expected for models evaluated in their development dataset compared with external datasets. The newly developed UHB model offers an advantage over the ISARIC 4C model in that it uses only routinely collected patient data recorded at admission and does not require additional assessment and recording of specific comorbidities (which are often not routinely fully recorded at the point of admission).

In our time series analysis, we did not find strong evidence for trends in predictor coefficients over the first 8 days of admission, particularly for variables included in the final models, suggesting that time-dependent effects due to effect modification or selection bias in the first week are small. Another recent model derived from patients with COVID-19 in a Hong Kong hospital adopted the use of time-dependent routinely collected predictors; the model in the Hong Kong study demonstrated high discrimination, with an AUROC of 0.91 when predicting severe COVID-19 outcomes.^20^ However, this model is yet to be peer-reviewed and externally validated.

### Strengths and limitations

The UHB dataset represents one of the largest and most ethnically diverse patient cohorts within the UK. Additionally, as part of the early UHB response to the COVID-19 pandemic, the hospital trust ensured that, on admission, all patients underwent a wide range of investigations to support international research efforts examining prognostic markers. This allowed us to examine a wide range of possible predictors (63 candidate predictors after exclusions). Lastly, a strength of this study was the good performance, in terms of both discrimination and calibration, of the simplified, reduced model in an externally validated cohort (CovidCollab), indicating its suitability for wider use, including potentially in LMICs.

Despite the strengths, the findings must be considered in light of the study’s limitations. Although we were able to use a derivation dataset from UHB with low levels of missing data, the overall sample size was relatively small compared with that of the ISARIC study and was limited to one UK geographical location. However, we were able to externally validate the model in a larger external cohort. A second limitation was that in the external validation cohort, we were unable to examine all of the predictors included in the original full UHB model due to only a reduced set of candidate predictors being available in CovidCollab. Nevertheless, the model performed well and the results suggest it may be applicable in a wide range of datasets where only a reduced set of predictor variables is available. It was not possible to carry out stratified analysis by ethnicity as, in the UHB dataset, too few patients were included in most of the strata; ethnicity data were not available in the CovidCollab dataset. Our definition of 28-day COVID-19 mortality aligns with the current technical guidance from Public Health England and the definition used by the UK government in reporting COVID-19 mortality statistics^21 22^; however, we acknowledge that this may not capture all COVID-19-related deaths, and some other studies have used a longer period of follow-up.^23^

## Conclusion

In this paper, we have described the development and external validation of novel prognostic models which predict mortality and ITU admission within 28 days of admission for patients admitted to hospital with COVID-19. The simple, reduced models used only routinely collected data gathered at admission, showed good discrimination and calibration, performed at least as well as the existing ISARIC 4C score and performed well in a validation cohort. The models can be integrated into existing electronic medical records systems to calculate each individual patient’s probability of death or ITU admission at the time of hospital admission. The models should be further validated to determine their applicability in other populations. In addition, implementation of the models and clinical utility should be evaluated.

## Supplementary Material

Reviewer comments

Author's manuscript

## Data Availability

No data are available.
