# Supplementary material for: Development and external validation of prognostic models for COVID-19 to support risk stratification in secondary care
Source: BMJ Open. 2022 Jan 17;12(1):e049506. doi: 10.1136/bmjopen-2021-049506 (PMC8764710; doi:10.1136/bmjopen-2021-049506)
Supplement: Supplementary data [file bmjopen-2021-049506supp001.pdf]

Supplementary Appendix 1. Methods

Continuous variables with non-linear associations

To represent continuous variables with non-linear relationships to the logit of the outcome, we initially fitted an exploratory predictive model (gam from the mgcv package in R), representing continuous features as thin-plate smoothed splines with the smoothing factor (gamma) set to 1.4 to shrink coefficient degrees of freedom. Based on visual inspection for obvious departure for linearity, if the variable appeared to have a linear relationship with the response term, we represented it as a linear term in variable selection and model testing and training. If the relationship between the response and the variable of interest appeared to be non-linear, we represented the feature as a spline term ("bs" from splines package) with the minimum degrees of freedom (3 d.f.) to avoid overfitting.

Supplementary Appendix 2: Results

28-day mortality outcome: UHB model – analyses exploring different ways of handling of missing data

We used different methods of handling missing data in order to explore the impact on the model performance: in primary analysis, missing values were imputed; in secondary analysis, missing categories were used. For the 28-day mortality outcome, the model derived using logistic stepwise selection using categorised variables with a missing category (rather than imputed data) gave a slightly higher AUROC (0.805, 95% CI 0.776-0.835); however, it was felt the inclusion of missing categories rather than imputed values might impact generalisability of the model outside the UHB/derivation data context. Other statistical modelling techniques (LASSO, GBM) offered no improvement in model performance and the models would be more difficult to implement in clinical practice. Using continuous predictors modelled using splines where non-linear relationship with the outcome was observed (Supplementary Figure 2) rather than categorising offered a small improvement in model performance (AUROC 0.792, 95% CI 0.762-0.822), but in the interests of clinical utility and interpretability the categorical model was favoured.

ITU admission: UHB model – analyses exploring different ways of handling of missing data

Again, for the ITU admission outcome, use of a missing category rather than imputed missing values improved model performance but was not selected as the final model due to potential problems with generalisability (AUROC 0.960, 95% CI 0.944-0.976). Models using continuous rather than categorical predictors (with splines where relevant; Supplementary Figure 3) gave a small improvement in AUROC (0.910, 95% CI 0.886-0.933), but were deemed less practical.

Supplementary Table 1. Baseline demographic characteristics, comorbidities and symptoms of participants admitted with COVID-19 in the derivation (University Hospitals Birmingham) and validation (CovidCollab) datasets stratified by intensive therapy unit admission

	Development cohort (UHB)			External validation cohort (CovidCollab)		
	Total	Not admitted to ITU	Admitted to ITU	Total	Not admitted to ITU	Admitted to ITU
N	1040	857	183	6099	5377	722
Age category (years), n (%)						
<30	35 (3.4)	29 (3.4)	6 (3.3)	125 (2.0)	102 (1.9)	23 (3.2)
30-39	42 (4.0)	29 (3.4)	13 (7.1)	270 (4.4)	228 (4.2)	42 (5.8)
40-49	91 (8.8)	63 (7.4)	28 (15.3)	497 (8.1)	388 (7.2)	109 (15.1)
50-59	146 (14.0)	90 (10.5)	56 (30.6)	793 (13.0)	615 (11.4)	178 (24.7)
60-69	181 (17.4)	136 (15.9)	45 (24.6)	944 (15.5)	751 (14.0)	193 (26.7)
70-79	220 (21.2)	189 (22.1)	31 (16.9)	1325 (21.7)	1185 (22.0)	140 (19.4)
80-89	214 (20.6)	210 (24.5)	4 (2.2)	1571 (25.8)	1535 (28.5)	36 (5.0)
≥90	111 (10.7)	111 (13.0)	0 (0.0)	574 (9.4)	573 (10.7)	1 (0.1)
Gender (male), n (%)	589 (56.6)	456 (53.2)	133 (72.7)	3361 (55.1)	2877 (53.5)	484 (67.0)
Ethnicity, n (%)				Not available		
White	590 (56.7)	519 (60.6)	71 (38.8)			
South Asian	127 (12.2)	99 (11.6)	28 (15.3)			
Black	68 (6.5)	56 (6.5)	12 (6.6)			
Other	255 (24.5)	183 (21.4)	72 (39.3)			
Comorbidities, n (%)						
Dementia	373 (35.9)	297 (34.7)	76 (41.5)	934 (15.3)	925 (17.2)	9 (1.2)
Cancer	135 (13.0)	124 (14.5)	11 (6.0)	649 (10.6)	585 (10.9)	64 (8.9)
Asthma	165 (15.9)	139 (16.2)	26 (14.2)	1579 (25.9)*	1404 (26.1)*	175 (24.2)*
Chronic obstructive pulmonary disease	283 (27.2)	241 (28.1)	42 (23.0)			
Sleep apnoea	49 (4.7)	42 (4.9)	7 (3.8)			
Cardiovascular disease	567 (54.5)	479 (55.9)	88 (48.1)	3033 (49.7)	2719 (50.6)	314 (43.5)
Hypertension	661 (63.6)	539 (62.9)	122 (66.7)	Not available		
Diabetes without complications	258 (24.8)	202 (23.6)	56 (30.6)	1794 (29.4)	1559 (29.0)	235 (32.5)
Diabetes with complications	112 (10.8)	103 (12.0)	9 (4.9)	Not available		
Peptic ulcer	29 (2.8)	26 (3.0)	3 (1.6)	Not available		
Liver disease	71 (6.8)	60 (7.0)	11 (6.0)	Not available		
Rheumatic/inflammatory disease	51 (4.9)	49 (5.7)	2 (1.1)	Not available		
Thyroid disorder	107 (10.3)	100 (11.7)	7 (3.8)	Not available		
ISARIC comorbidity score				Not applicable		
0	111 (10.7)	92 (10.7)	19 (10.4)			
1	234 (22.5)	176 (20.5)	58 (31.7)			
≥2	695 (66.8)	589 (68.7)	106 (57.9)			
Symptoms, n (%)						
Breathlessness	559 (59.2)	424 (54.7)	135 (79.9)	Not available		
Chest pain	39 (4.1)	29 (3.7)	10 (5.9)	Not available		
Cough	538 (57.0)	415 (53.5)	123 (72.8)	4259 (69.8)	3646 (67.8)	613 (84.9)
Fever	465 (49.3)	357 (46.1)	108 (63.9)	3212 (52.7)	2694 (50.1)	518 (71.7)
Headache	42 (4.4)	30 (3.9)	12 (7.1)	Not available		
Malaise	186 (19.7)	144 (18.6)	42 (24.9)	Not available		
New onset diarrhoea or vomiting	56 (5.9)	49 (6.3)	7 (4.1)	Not available		
Sputum	84 (8.9)	60 (7.7)	24 (14.2)	Not available		
Delirium	80 (8.5)	77 (9.9)	3 (1.8)	1160 (20.1)	1085 (21.3)	75 (10.9)
Outcomes						
Died within 28 days of admission	288 (27.7)	237 (27.7)	51 (27.9)	1668 (27.3)	1423 (26.5)	245 (33.9)
ITU admission within 28 days	183 (17.6)	0 (0.0)	183 (100.0)	722 (11.8)	0 (0.0)	722 (100.0)

*In CovidCollab, asthma, chronic obstructive pulmonary disease and sleep apnoea were combined as 'respiratory diseases'; data on the individual conditions was not available, therefore the n (%) given is for all respiratory diseases.

Supplementary Table 2. Area under the receiver operating characteristic curve (AUROC) for each of the models assessed in the derivation dataset (University Hospitals Birmingham) for predicting death within 28 days of hospital admission

Model	Continuous data	Missing continuous data	Candidate predictors included	AUROC (95% CI)
Logistic	Categorical	Imputed	All	0.743 (0.702-0.783)
LASSO	Categorical	Imputed	All	0.755 (0.720-0.791)
GBM	Categorical	Imputed	All	0.758 (0.719-0.797)
Logistic	Categorical	Imputed	Stepwise selection	0.779 (0.744-0.813)
Logistic	Categorical	Imputed	All plus comorbidities	0.730 (0.688-0.773)
Logistic	Categorical	Imputed	Demographic only	0.709 (0.676-0.742)
Logistic	Categorical	Missing category	All	0.773 (0.740-0.806)
Logistic	Categorical	Missing category	Demographic only	0.705 (0.670-0.740)
Logistic	Categorical	Missing category	Stepwise selection	0.805 (0.776-0.835)
LASSO	Categorical	Missing category	All	0.786 (0.750-0.823)
GBM	Categorical	Missing category	All	0.778 (0.745-0.811)
Logistic	Continuous	Imputed	All	0.768 (0.734-0.802)
Logistic	Continuous	Imputed	All with splines*	0.730 (0.652-0.807)
LASSO	Continuous	Imputed	All	0.778 (0.745-0.811)
LASSO	Continuous	Imputed	All with splines*	0.786 (0.755-0.817)
GBM	Continuous	Imputed	All	0.783 (0.750-0.816)
GBM	Continuous	Imputed	All with splines*	0.775 (0.742-0.809)
Logistic	Continuous	Imputed	Stepwise selection	0.794 (0.765-0.824)
Logistic	Continuous	Imputed	Stepwise selection with splines*	0.792 (0.762-0.822)

All = all demographic, symptoms, vital signs/physiological measures, and laboratory test result candidate predictors. *Splines were used to model continuous variables where non-liner associations with the outcome were observed. LASSO = least absolute shrinkage and selection operator; GBM = gradient boosting machine.

Supplementary Table 3. Logistic regression model coefficients for mortality within 28 days of admission in the derivation dataset (University Hospitals Birmingham)

Logistic regression equation:

$$\ln\left(\frac{p}{1-p}\right) = \beta_0 + \sum_{i=1}^{m} \beta_i x_i$$

Where p is probability, β_0 is the intercept (constant term), and β_i are the coefficients for each of the predictors x_i.

To calculate probability (p):

$$p = \frac{1}{1 + e^{-(\beta_0 + \sum_{i=1}^{m} \beta_i x_i)}}$$

Predictor	Coefficient (95% CI)
Intercept	-4.143 (-5.269--3.018)
Age category (years), n (%)	
<30	-13.985 (-746.83-718.86)
30-39	-0.609 (-1.929-0.710)
40-49	-1.353 (-2.557--0.149)
50-59	Ref
60-69	0.201 (-0.438-0.839)
70-79	0.978 (0.364-1.591)
80-89	1.353 (0.691-2.014)
≥90	1.705 (0.960-2.451)
Breathlessness	0.310 (-0.086-0.706)
Sputum	0.383 (-0.187-0.952)
Temperature (Celsius), n (%)	
<37.8	Ref
≥37.8	0.286 (-0.161-0.733)
Systolic blood pressure (mm Hg), n (%)	
<140	Ref
≥140	-0.340 (-0.695-0.016)
Respirations (per minute), n (%)	
<20	Ref
≥20	0.405 (0.035-0.775)
Oxygen saturation (%), n (%)	
<80	1.042 (-0.503-2.588)
80-88	1.425 (0.699-2.151)
89-93	0.332 (-0.085-0.749)
≥94	Ref
Portable oxygen concentrator fraction of inspired oxygen (%), n (%)	
≤0.28	Ref

0.28-0.49	0.624 (-0.069-1.316)
≥0.5	0.564 (-0.021-1.149)
pH, n (%)	
<7.30	0.344 (-0.308-0.997)
7.30-7.34	-0.347 (-0.931-0.238)
7.35-7.44	Ref
≥7.45	0.088 (-0.386-0.561)
White blood cell count (10^9/l), n (%)	
<3.9	-0.872 (-1.649--0.095)
3.9-10.8	Ref
≥10.9	0.158 (-0.252-0.568)
Platelets (10^9/l), n (%)	
<150	0.442 (-0.007-0.892)
150-399	Ref
≥400	-0.393 (-1.051-0.265)
C-reactive protein (mg/l), n (%)	
<10	Ref
10-99	0.613 (-0.312-1.538)
≥100	1.013 (0.109-1.917)
Glucose (mmol/l), n (%)	
<7.8	Ref
7.8-8.4	0.559 (-0.016-1.135)
≥8.5	0.359 (-0.005-0.723)
Alkaline phosphatase (U/l), n (%)	
<130	Ref
≥130	0.327 (-0.095-0.750)
Urea (mmol/l), n (%)	
<7.8	Ref
≥7.8	0.386 (0.035-0.738)
Corrected calcium (mmol/l), n (%)	
<2.2	0.575 (0.092-1.058)
2.2-2.5	Ref
≥2.6	1.057 (0.265-1.849)
Eosinophils (10^9/l), n (%)	
≤0.4	Ref
>0.4	-1.512 (-3.654-0.630)
Frailty score, n (%)	
1-3	Ref
4-6	0.345 (-0.231-0.920)
7-9	0.738 (0.113-1.363)

Supplementary Table 4. Area under the receiver operating characteristic curve (AUROC) for each of the models assessed in the derivation dataset (University Hospitals Birmingham) for predicting intensive therapy unit admission within 28 days of hospital admission

Model	Continuous data	Missing continuous data	Candidate predictors included	AUROC (95% CI)
Logistic	Categorical	Imputed	All	0.854 (0.824-0.885)
LASSO	Categorical	Imputed	All	0.877 (0.848-0.906)
GBM	Categorical	Imputed	All	0.874 (0.844-0.904)
Logistic	Categorical	Imputed	Stepwise selection	0.893 (0.864-0.922)
Logistic	Categorical	Imputed	All plus comorbidities	0.872 (0.835-0.909)
Logistic	Categorical	Imputed	Demographic only	0.766 (0.732-0.799)
Logistic	Categorical	Missing category	All	0.909 (0.858-0.960)
Logistic	Categorical	Missing category	Demographic only	0.764 (0.731-0.798)
Logistic	Categorical	Missing category	Stepwise selection	0.960 (0.944-0.976)
LASSO	Categorical	Missing category	All	0.961 (0.946-0.976)
GBM	Categorical	Missing category	All	0.956 (0.939-0.973)
Logistic	Continuous	Imputed	All	0.869 (0.837-0.901)
Logistic	Continuous	Imputed	All with splines*	0.892 (0.865-0.920)
LASSO	Continuous	Imputed	All	0.888 (0.860-0.915)
LASSO	Continuous	Imputed	All with splines*	0.903 (0.878-0.928)
GBM	Continuous	Imputed	All	0.896 (0.869-0.923)
GBM	Continuous	Imputed	All with splines*	0.899 (0.874-0.924)
Logistic	Continuous	Imputed	Stepwise selection	0.895 (0.869-0.922)
Logistic	Continuous	Imputed	Stepwise selection with splines*	0.910 (0.886-0.933)

All = all demographic, symptoms, vital signs/physiological measures, and laboratory test result candidate predictors. *Splines were used to model continuous variables where non-liner associations with the outcome were observed. LASSO = least absolute shrinkage and selection operator; GBM = gradient boosting machine.

Supplementary Table 5. Logistic regression model coefficients for intensive therapy unit admission within 28 days of hospital admission in the derivation dataset (University Hospitals Birmingham)

Predictor	Coefficient (95% CI)
Intercept	-0.459 (-2.077-1.159)
Age category (years), n (%)	
<30	-0.635 (-1.913-0.644)
30-39	-0.603 (-1.655-0.449)
40-49	-0.291 (-1.050-0.469)
50-59	Ref
60-69	-0.734 (-1.390--0.078)
70-79	-1.296 (-2.005--0.588)
80-89	-3.445 (-4.693--2.197)
≥90	-17.026 (-1075.95-1041.90)
Sex (male)	0.720 (0.214-1.226)
Fever	0.277 (-0.319-0.873)
New onset diarrhoea or vomiting	-0.937 (-2.090-0.217)
Temperature (Celsius), n (%)	
<37.8	Ref
≥37.8	0.619 (0.059-1.178)
Heart rate category (beats per minute), n (%)	
<80	Ref
80-99	-0.634 (-1.270-0.003)
≥100	-0.902 (-1.594--0.209)
Respirations (per minute), n (%)	
<20	Ref
≥20	0.799 (0.151-1.447)
Portable oxygen concentrator fraction of inspired oxygen (%), n (%)	
≤0.28	Ref
0.28-0.49	1.244 (0.357-2.131)
≥0.5	1.505 (0.686-2.324)
pH, n (%)	
<7.30	1.809 (0.861-2.757)
7.30-7.34	-0.104 (-0.958-0.750)
7.35-7.44	Ref
≥7.45	0.530 (-0.063-1.123)
White blood cell count (10^9/l), n (%)	
<3.9	-0.650 (-1.657-0.356)
3.9-10.8	Ref
≥10.9	1.372 (0.717-2.027)
C-reactive protein (mg/l), n (%)	
<10	Ref
10-99	0.227 (-0.913-1.367)
≥100	0.537 (-0.657-1.732)
Albumin (g/l), n (%)	
<25	Ref

25-34	-0.881 (-1.550--0.212)
≥35	-1.037 (-1.936--0.138)
Monocytes (10^9/l), n (%)	
<0.2	Ref
0.2-0.8	0.101 (-0.733-0.936)
>0.8	-0.972 (-2.067-0.122)
eGFR (ml/min), n (%)	
<30 (Stage 4 or above)	-0.731 (-1.506-0.045)
30-59 (Stage 3)	0.323 (-0.435-1.082)
60-89 (Stage 2)	-0.164 (-0.731-0.402)
>90 (Normal or high)	Ref
Frailty score, n (%)	
1-3	Ref
4-6	-0.522 (-1.047-0.002)
7-9	-2.080 (-3.117--1.044)
Glasgow Coma Scale score, n (%)	
<15	Ref
15	-1.348 (-1.896--0.801)

Supplementary Table 6. Logistic regression model coefficients for the external validation/reduced model (UHB-R) for mortality within 28 days of hospital admission (developed in University Hospitals Birmingham data and validated in CovidCollab)

Predictor	Coefficient (95% CI)
Intercept	-3.730 (-4.848--2.611)
Age category (years), n (%)	
<30	-14.108 (-766.78-738.56)
30-39	-0.617 (-1.922-0.689)
40-49	-1.333 (-2.487--0.178)
50-59	Ref
60-69	0.203 (-0.421-0.828)
70-79	0.925 (0.324-1.526)
80-89	1.204 (0.569-1.838)
≥90	1.590 (0.866-2.313)
Temperature (Celsius), n (%)	
<37.8	Ref
≥37.8	0.336 (-0.101-0.773)
Systolic blood pressure (mm Hg), n (%)	
<140	Ref
≥140	-0.353 (-0.691--0.015)
Respirations (per minute), n (%)	
<20	Ref
≥20	0.462 (0.120-0.804)
Oxygen saturation (%), n (%)	
<80	1.028 (-0.525-2.580)
80-88	1.491 (0.788-2.194)
89-93	0.480 (0.084-0.876)
≥94	Ref
Portable oxygen concentrator fraction of inspired oxygen (%), n (%)	
≤0.28	Ref
0.28-0.49	0.667 (0.027-1.307)
≥0.5	0.588 (0.028-1.148)
pH, n (%)	
<7.30	0.412 (-0.217-1.042)
7.30-7.34	-0.262 (-0.809-0.284)
7.35-7.44	Ref
≥7.45	0.186 (-0.269-0.640)
C-reactive protein (mg/l), n (%)	
<10	Ref
10-99	0.721 (-0.172-1.615)
≥100	1.167 (0.301-2.033)
Urea (mmol/l), n (%)	
<7.8	Ref
≥7.8	0.494 (0.159-0.828)
Frailty score, n (%)	

1-3	Ref
4-6	0.333 (-0.228-0.895)
7-9	0.685 (0.072-1.297)

Supplementary Table 7. Logistic regression model coefficients for the external validation/reduced model (UHB-R) model for intensive therapy unit admission within 28 days of hospital admission (developed in University Hospitals Birmingham data and validated in CovidCollab)

Predictor	Coefficient (95% CI)
Intercept	-2.482 (-4.012--0.951)
Age category (years), n (%)	
<30	-0.688 (-1.859-0.483)
30-39	-0.534 (-1.531-0.464)
40-49	-0.253 (-0.975-0.470)
50-59	Ref
60-69	-0.648 (-1.274--0.021)
70-79	-1.080 (-1.745--0.416)
80-89	-3.035 (-4.233--1.837)
≥90	-16.756 (-1103.28-1069.77)
Sex (male)	0.608 (0.112-1.105)
Fever	0.279 (-0.267-0.824)
Respirations (per minute), n (%)	
<20	Ref
≥20	0.648 (0.052-1.245)
Portable oxygen concentrator fraction of inspired oxygen (%), n (%)	
≤0.28	Ref
0.28-0.49	1.205 (0.392-2.018)
≥0.5	1.703 (1.011-2.396)
pH, n (%)	
<7.30	1.838 (0.917-2.759)
7.30-7.34	0.125 (-0.655-0.905)
7.35-7.44	Ref
≥7.45	0.621 (0.047-1.196)
C-reactive protein (mg/l), n (%)	
<10	Ref
10-99	-0.046 (-1.116-1.023)
≥100	0.456 (-0.632-1.544)
Neutrophil:lymphocyte ratio, n (%)	
<2.21	Ref
2.21-4.82	0.959 (-0.159-2.077)
>4.82	1.305 (0.212-2.399)
eGFR (ml/min), n (%)	
<30 (Stage 4 or above)	-0.598 (-1.331-0.136)
30-59 (Stage 3)	0.300 (-0.385-0.985)
60-89 (Stage 2)	-0.253 (-0.780-0.275)
>90 (Normal or high)	Ref
Frailty score, n (%)	
1-3	Ref
4-6	-0.475 (-0.979-0.028)
7-9	-1.991 (-3.025--0.957)

Glasgow Coma Scale score, n (%)	
<15	Ref
15	-1.310 (-1.821--0.798)

Supplementary Table 8. Sensitivity analysis – complete case analysis and exploration of different numbers of missing candidate predictors: Area under the receiver operating characteristic curve (AUROC) in the University Hospitals Birmingham dataset for predicting mortality and intensive therapy unit admission within 28 days of hospital admission

Number of missing predictor variables per patient	n	Outcome	
		Death	ITU
0	224	0.696 (0.597-0.795)	0.892 (0.844-0.940)
0-1	471	0.760 (0.708-0.812)	0.880 (0.846-0.915)
0-2	564	0.763 (0.715-0.812)	0.877 (0.842-0.911)
0-5	643	0.756 (0.712-0.799)	0.886 (0.854-0.918)
0-10	911	0.772 (0.739-0.806)	0.885 (0.857-0.914)

Supplementary Table 9. Sensitivity analysis – stratification by gender and age category: Area under the receiver operating characteristic curve (AUROC) in the University Hospitals Birmingham dataset for predicting mortality and intensive therapy unit admission within 28 days of hospital admission, using the reduced UHB-R model

Model		Outcome	
Derivation/Train	Test	Death	ITU
**Gender-stratified**			
UHB-R (fitted on all patients)	Male	0.775 (0.726-0.823)	0.897 (0.856-0.937)
UHB-R (fitted on all patients)	Female	0.755 (0.706-0.804)	0.873 (0.833-0.913)
**Age-stratified**			
UHB-R (fitted on all patients)	≤60 years	0.730 (0.638-0.823)	0.845 (0.761-0.930)
UHB-R (fitted on all patients)	>60 years	0.778 (0.722-0.834)	0.897 (0.864-0.930)

Supplementary figure legends:

Supplementary Figure 1. Calibration plot (observed probability (y-axis) against predicted probability (x-axis)) for ISARIC 4C score in UHB dataset (mortality outcome).

Supplementary Figure 2. Exploration of non-linear relationships between continuous predictors and 28-day mortality using general additive models (GAM); splines (continuous smoothed predictors) were included in the models where a non-linear relationship was observed.

Supplementary Figure 3. Exploration of non-linear relationships between continuous predictors and intensive therapy unit admission using general additive models (GAM); splines (continuous smoothed predictors) were included in the models where a non-linear relationship was observed.

Supplementary Figure 4. Forest plot of logistic regression coefficients for days 0-7 from admission for mortality outcome.
