# Supplementary figures and images for: Development and external validation of prognostic models for COVID-19 to support risk stratification in secondary care
Source: BMJ Open. 2022 Jan 17;12(1):e049506. doi: 10.1136/bmjopen-2021-049506 (PMC8764710; doi:10.1136/bmjopen-2021-049506)

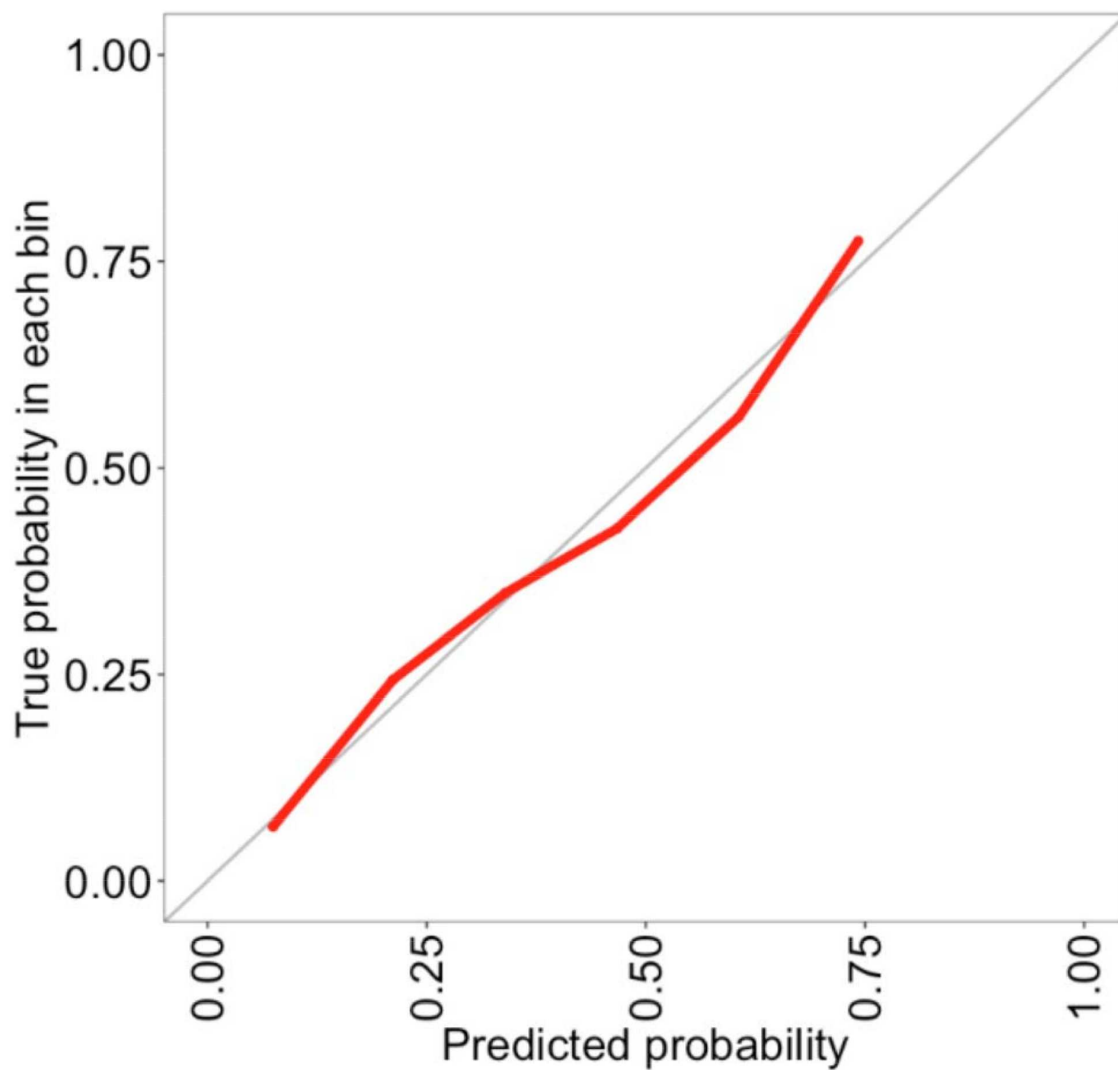

Supplement: Supplementary data [file bmjopen-2021-049506supp002.pdf]

# Supplemental material

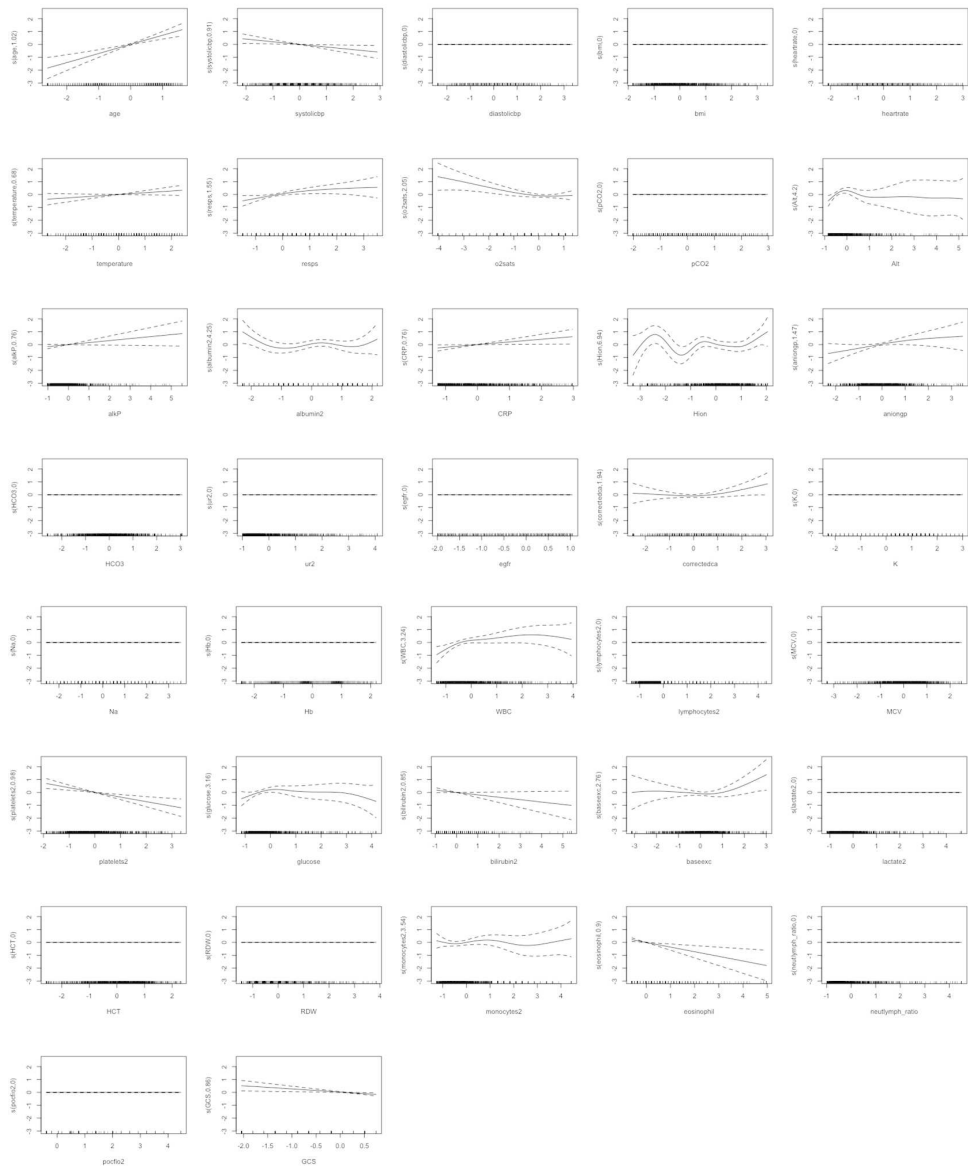

Supplement: Supplementary data [file bmjopen-2021-049506supp003.pdf]

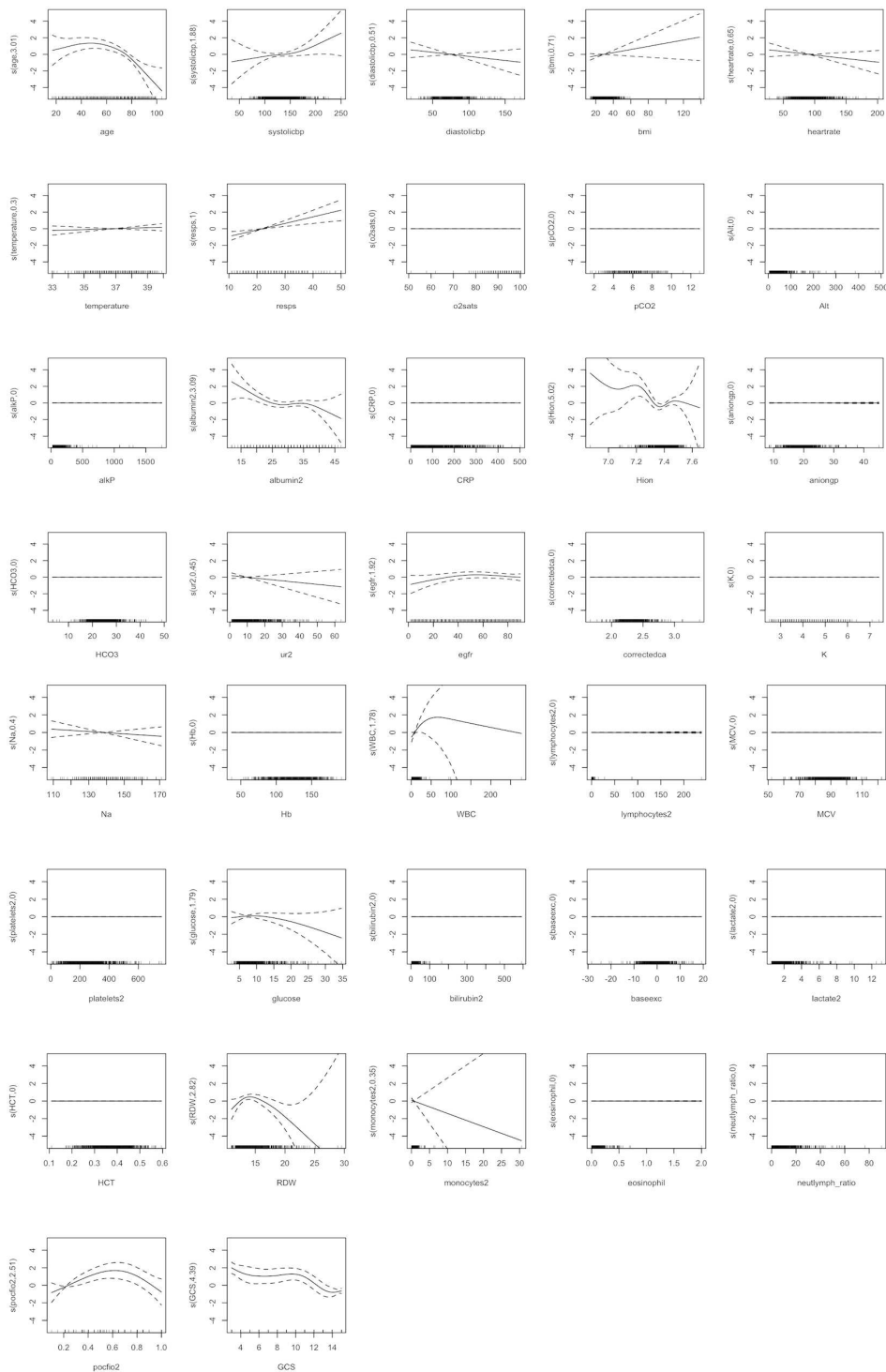

Supplement: Supplementary data [file bmjopen-2021-049506supp004.pdf]

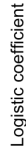

Supplement: Supplementary data [file bmjopen-2021-049506supp005.pdf]
