# Supplementary material for: Development and external validation of prognostic models for COVID-19 to support risk stratification in secondary care
Source: BMJ Open. 2022 Jan 17;12(1):e049506. doi: 10.1136/bmjopen-2021-049506 (PMC8764710; doi:10.1136/bmjopen-2021-049506)
Supplement: Author's manuscript [file bmjopen-2021-049506.draft_revisions.pdf]

BMJ Open

BMJ Open is committed to open peer review. As part of this commitment we make the peer review history of every article we publish publicly available.

When an article is published we post the peer reviewers' comments and the authors' responses online. We also post the versions of the paper that were used during peer review. These are the versions that the peer review comments apply to.

The versions of the paper that follow are the versions that were submitted during the peer review process. They are not the versions of record or the final published versions. They should not be cited or distributed as the published version of this manuscript.

BMJ Open is an open access journal and the full, final, typeset and author-corrected version of record of the manuscript is available on our site with no access controls, subscription charges or pay-per-view fees (http://bmjopen.bmj.com).

If you have any questions on BMJ Open's open peer review process please email info.bmjopen@bmj.com

BMJ Open

Development and external validation of prognostic models for COVID-19 to support risk stratification in secondary care

Journal:	*BMJ Open*
Manuscript ID	bmjopen-2021-049506
Article Type:	Original research
Date Submitted by the Author:	26-Jan-2021
Complete List of Authors:	Adderley, Nicola; University of Birmingham, Institute of Applied Health Research<br>Taverner, Thomas ; University of Birmingham, Institute of Applied Health Research<br>Price, Malcolm; University of Birmingham, Institute of Applied Health Research<br>Sainsbury, Christopher; University of Birmingham College of Medical and Dental Sciences, Institute of Applied Health Research<br>Greenwood, David; University of Birmingham College of Medical and Dental Sciences, Institute of Applied Health Research<br>Chandan, Joht; University of Birmingham College of Medical and Dental Sciences, Institute of Applied Health Research<br>Takwoingi, Yemisi; University of Birmingham, Institute of Applied Health Research<br>Haniffa, Rashan; University of Oxford<br>Hosier, Isaac; University of Birmingham College of Medical and Dental Sciences, Institute of Applied Health Research<br>Welch, Carly; University of Birmingham, Institute of Inflammation and Ageing<br>Parekh, Dhruv; University of Birmingham, Institute of Inflammation and Ageing<br>Gallier, Suzy; University Hospitals Birmingham NHS Foundation Trust<br>Gokhale, Krishna; University of Birmingham<br>Denniston, Alastair; University Hospitals Birmingham NHS Foundation Trust, Department of Ophthalmology<br>Sapey, Elizabeth; University of Birmingham, PIONEER HDR-UK Hub<br>Nirantharakumar, Krishnarajah; University of Birmingham, Institute of Applied Health Research
Keywords:	COVID-19, PUBLIC HEALTH, Public health < INFECTIOUS DISEASES

SCHOLARONE™
Manuscripts

I, the Submitting Author has the right to grant and does grant on behalf of all authors of the Work (as defined in the below author licence), an exclusive licence and/or a non-exclusive licence for contributions from authors who are: i) UK Crown employees; ii) where BMJ has agreed a CC-BY licence shall apply, and/or iii) in accordance with the terms applicable for US Federal Government officers or employees acting as part of their official duties; on a worldwide, perpetual, irrevocable, royalty-free basis to BMJ Publishing Group Ltd ("BMJ") its licensees and where the relevant Journal is co-owned by BMJ to the co-owners of the Journal, to publish the Work in this journal and any other BMJ products and to exploit all rights, as set out in our licence.

The Submitting Author accepts and understands that any supply made under these terms is made by BMJ to the Submitting Author unless you are acting as an employee on behalf of your employer or a postgraduate student of an affiliated institution which is paying any applicable article publishing charge ("APC") for Open Access articles. Where the Submitting Author wishes to make the Work available on an Open Access basis (and intends to pay the relevant APC), the terms of reuse of such Open Access shall be governed by a Creative Commons licence – details of these licences and which Creative Commons licence will apply to this Work are set out in our licence referred to above.

Other than as permitted in any relevant BMJ Author's Self Archiving Policies, I confirm this Work has not been accepted for publication elsewhere, is not being considered for publication elsewhere and does not duplicate material already published. I confirm all authors consent to publication of this Work and authorise the granting of this licence.

For peer review only

Development and external validation of prognostic models for COVID-19 to support risk stratification in secondary care

Nicola J Adderley PhD,[1] Thomas Taverner PhD,[1] Malcolm Price PhD,[1,2] Christopher Sainsbury MD,[1,3] David Greenwood MSc,[1] Joht Singh Chandan PhD,[1] Yemisi Takwoingi PhD,[1,2] Rashan Haniffa PhD,[4,5,6] Isaac Hosier BMedSci,[1] Carly Welch MRCP,[7,8,9] Dhruv Parekh PhD,[7,8] Suzy Gallier BSc,[8] Krishna Gokhale MSc,[1] Alastair K Denniston PhD FRCOphth,[7,10,11] Elizabeth Sapey FRCP,[7,8,11] Krishnarajah Nirantharakumar MD[1,11]

Affiliations:

1. Institute of Applied Health Research, University of Birmingham, Edgbaston, Birmingham, B15 2TT, UK
2. NIHR Birmingham Biomedical Research Centre, University Hospitals Birmingham NHS Foundation Trust and University of Birmingham, UK
3. Department of Diabetes, Gartnavel General Hospital, NHS Greater Glasgow and Clyde, Glasgow, UK
4. National Intensive Care Surveillance-MORU, Colombo, Sri Lanka
5. Mahidol Oxford Tropical Medicine Research Unit, University of Oxford
6. University College London Hospitals, London, United Kingdom
7. Institute of Inflammation and Ageing, University of Birmingham, Birmingham, UK
8. University Hospitals Birmingham NHS Foundation Trust, Birmingham, UK
9. Medical Research Council and Versus Arthritis Centre for Musculoskeletal Ageing Research, University of Birmingham and University of Nottingham, Birmingham and Nottingham, UK
10. University College London/Moorfields Eye Hospital National Institute for Health Research Biomedical Research Centre
11. Health Data Research UK

Corresponding author:
Nicola J Adderley, n.j.adderley@bham.ac.uk
ORCID: 0000-0003-0543-3254

Abstract: 277
Word count: 5404
Tables: 4 (+9 supplementary)
Figures: 1 (+3 supplementary)

For peer review only - http://bmjopen.bmj.com/site/about/guidelines.xhtml

Article Summary

Strengths and limitations of this study

- We developed novel prognostic models predicting mortality and ITU admission within 28 days of admission for patients hospitalised with COVID-19, using a large routinely collected dataset gathered at admission with a wide range of possible predictors (demographic variables, symptoms, physiological measures, imaging, laboratory test results).
- These novel models showed good discrimination and calibration in both derivation and external validation cohorts, and outperformed the existing ISARIC model and 4C score in the derivation dataset. We found that addition of comorbidities to the set of candidate predictors included in model derivation did not improve model performance.
- If integrated into hospital electronic medical records systems, the model algorithms will provide a predicted probability of mortality or ITU admission for each patient based on their individual data at, or close to, the time of admission, which will support clinicians' decision making with regard to appropriate patient care pathways and triage. This information might also assist clinicians in explaining complex prognostic assessments and decisions to patients and their relatives.
- A limitation of the study was that in the external validation cohort we were unable to examine all of the predictors included in the original full UHB model due to only a reduced set of candidate predictors being available in CovidCollab. Nevertheless, the reduced model performed well and the results suggest it may be applicable in a wide range of datasets where only a reduced set of predictor variables is available.
- Furthermore, it was not possible to carry out stratified analysis by ethnicity as the UHB dataset contained too few patients in most of the strata, and no ethnicity data was available in the CovidCollab dataset.

For peer review only - http://bmjopen.bmj.com/site/about/guidelines.xhtml

Abstract

Objectives

Existing UK prognostic models for patients admitted to hospital with COVID-19 are limited by reliance on comorbidities, which are under-recorded in secondary care, and lack of imaging data among the candidate predictors. Our aims were to develop and externally validate novel prognostic models for adverse outcomes (death, intensive therapy unit (ITU) admission) in UK secondary care; and externally validate the existing 4C score.

Design

Candidate predictors included demographic variables, symptoms, physiological measures, imaging, laboratory tests. Final models used logistic regression with stepwise selection.

Setting

Model development was performed in data from University Hospitals Birmingham (UHB). External validation was performed in the CovidCollab dataset.

Participants

Patients with COVID-19 admitted to UHB January-August 2020 were included.

Main outcome measures

Death and ITU admission within 28 days of admission.

Results

1040 patients with COVID-19 were included in the derivation cohort; 288 (28%) died and 183 (18%) were admitted to ITU within 28 days of admission. Area under the receiver operating curve (AUROC) for mortality was 0.791 (95%CI 0.761-0.822) in UHB and 0.767 (95%CI 0.754-0.780) in CovidCollab; AUROC for ITU admission was 0.906 (95%CI 0.883-0.929) in UHB and 0.811 (95%CI 0.795-0.828) in CovidCollab. Models showed good calibration. Addition of comorbidities to candidate predictors did not improve model performance. AUROC for the 4C score in the UHB dataset was 0.754 (95%CI 0.721-0.786).

Conclusions

The novel prognostic models showed good discrimination and calibration in derivation and external validation datasets, and outperformed the existing 4C score. The models can be integrated into electronic medical records systems to calculate each individual patient's probability of death or ITU admission at the time of hospital admission. Implementation of the models and clinical utility should be evaluated.

For peer review only - http://bmjopen.bmj.com/site/about/guidelines.xhtml

Background

The coronavirus disease 2019 (COVID-19) pandemic has placed exceptional strain on health care systems globally – in high income as well as low and middle income countries (LMIC). Health systems, and especially critical care services, can be overwhelmed given the number of patients, and the duration and severity of their illness. A proportion of patients with COVID-19 can deteriorate rapidly. Clinicians need to differentiate between those with COVID-19 who are at high risk of the most severe symptoms (requiring intensive care treatment/ventilation) or death, and those who can be considered low risk and potentially managed in the community. Early identification of patients at highest risk of severe outcomes may provide opportunity to prioritise, intervene and improve outcomes.

Objective prognostic tools for patients with COVID-19 would be of considerable benefit to clinicians in a secondary care setting. Prognostic models that can accurately discriminate between patients who will progress to more severe symptoms or death, and those who do not, can be used by clinicians to triage and manage patients. Such models based on patients' initial characteristics, symptoms, biomarkers and imaging at the time of hospital admission can be used at or just after admission. This could potentially reduce time to appropriate interventions and improve patient outcomes.

A rapid systematic review has identified a number of prediction models developed for COVID-19, including prognostic models.[1] However, while these existing studies provided useful information on candidate predictors for further exploration, there were substantial limitations: many models were developed exclusively in a Chinese population; many were at high risk of bias, particularly in terms of inclusion of non-representative control participants, inappropriate exclusion criteria, and small sample sizes leading to high risk of overfitting; most models have not considered imaging findings as candidate predictor variables; and external validation was limited.

More recent models have since been developed,[2,3] some of which overcome a number of these limitations, including the International Severe Acute Respiratory and emerging Infection Consortium (ISARIC) model and corresponding (simplified) 4C score, which was developed in a UK secondary care population representing 260 hospitals in England, Scotland and Wales (the ISARIC dataset).[3] Whilst the 4C score showed reasonable discrimination for mortality, there are a number of limitations, including a reliance on clinicians counting specific comorbidities, which are known to be under-recorded in secondary care,[4] and an absence of imaging data among the candidate predictors. Furthermore, it is unclear if this model can help with predicting deteriorating patients beyond day of admission. The latter is important given the unpredictable clinical course of COVID-19 patients in the early days after admission. This raises two important research questions: first, to what extent does the inclusion of comorbidities and additional patient information (such as imaging and additional biomarkers) improve prognostic models for hospitalised patients with COVID-19? And second, is there any added value in updating the clinical parameters with evolving biomarkers to improve prediction of the clinical course of patients, with patients reassessed in real-time as the disease evolves?

For peer review only - http://bmjopen.bmj.com/site/about/guidelines.xhtml

The overarching aims of this study were to novel develop prognostic models for adverse outcomes (death, intensive therapy unit (ITU) admission) in a UK secondary care setting; to externally validate these models in an international dataset (including data from UK hospitals); to externally validate the existing UK ISARIC model and 4C score;[3] and to compare performance of the newly developed models with the UK ISARIC model and 4C score. In addition, we developed daily models using time series data from the first eight days from admission to explore changes in predictors over time.

Methods

Data source

Data from University Hospitals Birmingham NHS Foundation Trust (UHB) was sourced via the PIONEER Health Data Research Hub for acute care, and used for model development and for external validation of the ISARIC model and 4C score. Data from patients with COVID-19 admitted to Queen Elizabeth Hospital, Birmingham (part of UHB), between 1st January 2020 and 16th August 2020 was included. Data included symptoms recorded at admission, comorbidities (from International Classification of Diseases 10th revision (ICD-10) discharge codes), vital signs (e.g. blood pressure, oxygen saturation), laboratory results (biochemistry, haematology, microbiology, pathology), imaging, and outcomes (ITU admission and death).

External validation of the newly developed models was performed in the CovidCollab dataset. CovidCollab is an international project utilising routinely collected health care data to develop a better understanding of how best to treat and care for adults with COVID-19.[5,6] The dataset includes symptoms, comorbidities, vital signs, laboratory results, imaging findings, and outcomes.

For peer review only

Study population

Patients of all ages diagnosed with COVID-19 and hospitalised were included. Diagnosis was defined as a positive test result for SARS-CoV-2 from one or more reverse transcription polymerase chase reaction (RT-PCR) or transcription mediated amplification tests. In the CovidCollab dataset, COVID-19 diagnosis was by either PCR or antibody test.

Study Design

Retrospective cohort analyses; index date (start of follow-up) was the hospital admission date. Study period was 1st January 2020 to 12th September 2020 (last admission date was 16th August to ensure a minimum of 28 days of follow-up).

Outcomes

Primary outcome was death within 28 days of admission (in-hospital or post-discharge). Secondary outcome was ITU admission within 28 days of admission.

For peer review only - http://bmjopen.bmj.com/site/about/guidelines.xhtml

Study follow-up

Participants were followed from index (admission) date until the earliest of outcome date or study end (latest available data, 12th September 2020). Participants were censored 28 days after index date. Participants admitted after 16th August 2020 (less than 28 days prior to study end date) were excluded.

Candidate predictor variables

Candidate predictors were selected a priori following a review of existing literature, discussion with clinical experts (specialists in acute care, critical care and geriatric medicine), and based on availability of variables routinely collected in secondary care/UHB. These included demographic variables, symptoms, comorbidities, physiological measures, imaging findings and laboratory test results. Comorbidities are not reliably and completely collected at admission, with the most complete hospital record of comorbidities usually being the discharge ICD-10 codes; therefore, the development and performance of models with and without comorbidity predictors were compared in order to explore the potential for developing models which would require no additional data collection (other than routinely collected data) at the point of admission.

Model development

Models were trained using UHB data (patients admitted up to and including 16th August 2020). We used a multi-stage model building process that assessed the impact of a range of feature representation and modelling choices to select important candidate predictors. All analyses were performed in R.

Three sets of models were fitted which incorporated continuous variables in three different ways, to explore the impact of treating these variables as continuous or categorical, and also to explore the impact of different methods of handling missing data:

(a) as continuous numeric values, with missing values imputed ("continuous");
(b) as categorical values derived from the imputed continuous values ("categorical-imputed").
(c) and, in secondary analysis, as categorical values, using clinically meaningful categories and reference ranges, with missing indicators as a separate category ("categorical");

To represent continuous variables with nonlinear relationships to the logit of the outcome, we initially fitted an exploratory predictive model (gam from the mgcv package in R), representing continuous features as thin-plate smoothed splines with the smoothing factor (gamma) set to 1.4 to shrink coefficient degrees of freedom. Based on visual inspection for obvious departure for linearity, if the variable appeared to have a linear relationship with the response term, we represented it as a linear term in variable selection and model testing and training. If the relationship between the response and the variable of interest appeared to be nonlinear, we represented the feature as a spline term ("bs" from splines package) with the minimum degrees of freedom (3 d.f.) to avoid overfitting.

For peer review only - http://bmjopen.bmj.com/site/about/guidelines.xhtml

For the three ways of handling numeric features and missing variables above, we fitted outcomes of death within 28 days and ITU admission (within 28 days) to candidate predictors using a range of models, which allowed both linear relationships and complex interactions between variables:

(a) logistic regression with i) all baseline parameters (demographic variables, symptoms, vital signs/physiological measures, and laboratory test results), ii) demographic variables only, and iii) all baseline parameters with the addition of recorded comorbidities (recorded up to the point of discharge);
(b) logistic regression with stepwise AIC minimisation, both forward and backward;[7]
(c) LASSO (l1 penalized) logistic regression using all baseline parameters;
(d) gradient boosted model (GBM) using all baseline parameters with default hyperparameter values of 150 trees, maximum interaction depth 3, minimum of 10 observations in nodes, and shrinkage 0.1.[8]

For each of these four variable selection models, in order to reduce overfitting and selection bias, we internally validated using 5-fold cross-validation (80/20 train/test split) to derive the candidate variable list. To avoid sensitivity to imputation, this cross-validation was repeated for each of the 5 multiple imputations.

Due to the relatively small number of outcome events (<300) we did not attempt to systematically look for interactions between multiple variables.

Model performance (discrimination) was assessed by calculating the area under the receiver operating characteristics curve (AUROC, or C-statistic).[9] Calibration was assessed by plotting observed probability of the outcome against predicted probability, and by calculating the calibration slope and intercept. We also calculated sensitivity, specificity, positive predicted value (PPV) and negative predictive value (NPV) for the final models. For each feature set and each model, the final results for cross-validated (optimism-adjusted) AUROC and other metrics were combined from all the multiple imputations of the data set using Rubin's rules for the mean and confidence interval (derived from the standard deviation).[10]

Missing data

Information on candidate predictors was collected at the point of admission; however, where information on physiological or laboratory measures was not available on the day of admission, measures recorded up to 72 hours after admission were used. Candidate predictors for which >40% of patients had missing data were excluded from the analysis. Further missing continuous variables (vital signs, laboratory tests) and symptoms were imputed using the R “mice” (Multiple Imputation using Chained Equations) multiple imputation package; we performed 5 imputations, using predictive mean matching and a maximum of 50 iterations.[11] We also explored use of a missing category for missing test results. Absence of a record of a comorbidity was taken to indicate absence of the condition.

For peer review only - http://bmjopen.bmj.com/site/about/guidelines.xhtml

External validation

To investigate the transferability of models, we performed external validation of logistic regression models derived from the UHB dataset in the CovidCollab dataset, for predicting outcomes of 28-day mortality and ITU admission.

Not all candidate predictors were common to both datasets; therefore, new logistic regression models for death within 28 days and for ITU admission were re-fitted on the UHB data using only those variables also present in the CovidCollab data. We then performed an external validation of these UHB models in the CovidCollab dataset, and ascertained the AUROC in both the UHB and CovidCollab datasets. Based on model performance observed in the initial model derivation and in the interest of clinical utility, we used only categorical rather than continuous numeric variables, with imputed missing values (imputed prior to categorisation). To verify that predictors behaved similarly, we compared logistic coefficients from UHB to the same models fitted on the CovidCollab dataset. To account for sensitivity to missing values, we performed training and testing five times on 5-fold multiply imputed data sets for both UHB and CovidCollab.

External validation of ISARIC and 4C models

An extreme gradient boosting (XGBoost) model using the ISARIC study parameters and logistic regression using the 4C score was performed in the UHB dataset (following the same modelling methods used in the original ISARIC study). The ISARIC XGBoost model was calibrated to the UHB data by deriving UHB-specific coefficients for the included variables. Model performance was assessed by calculating the AUROC and plotting calibration curves.

Sensitivity analyses

Most patient records had some missing variables; we therefore performed a complete case analysis, where we re-fitted the best forward stepwise selection model to complete case data, then data with ≤1, 2, 5, and 10 missing values, imputing missing values in the same way as above, and examined AUROCs and logistic coefficients for stability.

In addition, we performed a sensitivity analysis within male and female strata by assessing the final model performance (AUROC) on male and female patients separately, and by comparing to performance of separate models which were fitted to male and female patients only.

Time series analysis

The UHB regression models utilised baseline measurement data collected on admission; where not available at admission, we accepted values up to 72 hours after admission. To investigate fine-grained temporal effects of data acquisition, we produced a series of separate logistic regression models using data collected at different time windows from within 24 hours of admission up to within 7 days of admission, in 1-day increments, for the mortality outcome. Each dataset included only those patients eligible at the end of the window (not dead or discharged). This created eight different sets of predictors, including baseline variables of age, gender, symptoms, and the time-sensitive variables of the latest physiological and laboratory measurements available.

For peer review only - http://bmjopen.bmj.com/site/about/guidelines.xhtml

For missing data, data were carried forward from the first observation (last observation carried forward, LOCF) and 5-fold multiple imputation was performed for missing data after LOCF was done, within each separate time-window dataset. Each model was trained and tested in 5-fold cross validation, within each imputation, and AUROCs averaged using Rubin's rule. We compared the AUROCs for each of the eight models for predicting 28-day mortality from the time of admission and compared the logistic coefficients for the models. For additional insight into possible effects of changing measurements, we produced an additional logistic model for 28-day mortality to time-sensitive data collected within 4 days of admission, augmented with predictors indicating an increase or decrease in the category of each time-sensitive predictor relative to the reference category from 0 to 4 days – for example, whether temperature had crossed from below to above 37.8°C in that period.

Patient and public involvement

We engaged with members of the PIONEER patient and public involvement group during development of the study protocol. We will further engage with this group, as well as other local and national patient and public involvement groups, in order to discuss dissemination of the findings and the best way to communicate these to patients and the public. We also consulted with several secondary care clinicians before and during the study to ensure that the tools developed meet the needs of clinicians. We have engaged with local NHS trusts to ensure that the algorithms developed are implemented/tested in a hospital setting.

Results

Derivation cohort characteristics

A total of 1040 participants with COVID-19 admitted to UHB were included in the derivation cohort. 288 (28%) died within 28 days of admission and 183 (18%) were admitted to ITU. Baseline characteristics are presented in Table 1. The mean (SD) age of participants was 68.2 (17.7) years; 57% (589) were male; and almost 90% had at least one comorbidity.

Candidate predictors

After exclusion of 7 candidate predictors with >40% missing data (D-dimer, ferritin, high sensitivity troponin, fibrinogen, lactate dehydrogenase, vitamin D, haemoglobin A1c), 63 predictors were considered for inclusion in the models:

Demographic characteristics: age, gender, ethnicity;

Symptoms (binary, presence or absence of symptom at admission): breathlessness, chest pain, cough, fever, headache, malaise, new onset diarrhoea or vomiting, sputum, delirium;

Physiological measures and vital signs: body mass index (BMI; kg/m^2), systolic blood pressure (mm Hg), diastolic blood pressure (mm Hg), temperature (degrees Celsius), heart rate (beats per minute), respiratory rate (respirations per minute), oxygen saturation (%), partial pressure of CO_2 (kPa), portable oxygen concentrator fraction of inspired oxygen (FiO_2; %);

For peer review only - http://bmjopen.bmj.com/site/about/guidelines.xhtml

Imaging: chest X-ray finding (categorised as clear/unchanged, local consolidation, ground glass opacity/bilateral infiltrates, other/no firm diagnosis, none performed/missing);

Scores: Frailty score (Rockwood Clinical Frailty Scale);[12] Glasgow Coma Scale score;[13,14]

Laboratory test results: estimated glomerular filtration rate (eGFR; ml/min), pH (%), base excess (mmol/l), anion gap (mmol/l), white blood cell (WBC) count (10^9/l), platelets (10^9/l), lymphocytes (10^9/l), neutrophil:lymphocyte ratio, mean corpuscular volume (fl), red cell distribution width (%), monocytes (10^9/l), eosinophils (10^9/l), haemoglobin (g/l), glucose (mmol/l), bicarbonate (mmol/l), C-reactive protein (mg/l), albumin (g/l), bilirubin (µmol/l), alanine aminotransferase (U/l), alkaline phosphatase (U/l), urea (mmol/l), potassium (mmol/l), sodium (mmol/l), corrected calcium (mmol/l), lactate (U/l) and haematocrit (l/l);

Comorbidities (binary), presence or absence of record in discharge ICD-10 codes): dementia, cancer, asthma, chronic obstructive pulmonary disease, sleep apnoea, cardiovascular disease, hypertension, diabetes without complications, diabetes with complications, peptic ulcer, liver disease, rheumatic/inflammatory disease, thyroid disorder.

28-day mortality outcome: UHB model and predictive performance

Area under the ROC curve values for each of the logistic, LASSO and GBM models, treating continuous variables in one of three ways (as continuous variables with imputed missing values; as clinically meaningful categorical variables with imputed missing values; and as categorical variables with missing categories), are presented in Supplementary Table 1.

The final model selected was a logistic regression using stepwise selection of variables with categorisation of continuous variables (with imputed missing values). The final 18 categorical predictors included in the model were: age, breathlessness, sputum, systolic blood pressure, temperature, respiratory rate, oxygen saturation, FiO_2, alkaline phosphatase, C-reactive protein, corrected calcium, eosinophils, glucose, pH, urea, WBC count, platelets, and frailty score. AUROC for the UHB cross-validated model was 0.778 (95 % CI 0.741-0.815) (Table 2). At a 20% predicted probability of mortality, sensitivity was 82% (95% CI 79-85), specificity was 59% (95% CI 56-61), positive predictive value was 43% (95% CI 41-45), and negative predictive was 90% (95% CI 88-91) (Table 3a). Calibration was very good at low to medium predicted probabilities, but was poorer at very high predicted probabilities; a calibration plot is shown in Figure 1A, calibration slope was 0.78 (95% CI 0.66-0.91) (Supplementary Table 2). Model coefficients (model equation) are shown in Supplementary Table 3.

We used different methods of handling missing data in order to explore the impact on the model performance: in primary analysis, missing values were imputed; in secondary analysis, missing categories were used. The model derived using logistic stepwise selection using categorised variables with a missing category (rather than imputed data) gave a slightly higher AUROC (0.805, 95% CI 0.777-0.834); however, it was felt the inclusion of missing categories rather than imputed values might impact generalisability of the model outside the UHB/derivation data context. Other statistical modelling techniques (LASSO, GBM) offered no improvement in model performance and the models would be more difficult to implement in clinical practice. Using continuous predictors modelled using splines where non-linear relationship with the outcome was observed (Supplementary

For peer review only - http://bmjopen.bmj.com/site/about/guidelines.xhtml

Figure 1) rather than categorising offered a small improvement in model performance (AUROC 0.791, 95% CI 0.759-0.823), but in the interests of clinical utility and interpretability the categorical model was favoured.

Addition of comorbidities to the candidate predictors included in the model did not improve performance of the model (Supplementary Table 1). Since comorbidities are known to be under-reported during acute presentations,[4] and they offered no improvement on model performance, models without comorbidities were preferred.

ITU admission: UHB model and predictive performance

Area under the ROC curve values for each of the models performed are presented in Supplementary Table 4.

The final model selected was a logistic regression using stepwise selection of variables with categorisation of continuous variables (with imputed missing values). The final 16 categorical predictors included in the model were: age, gender, fever, new onset diarrhoea or vomiting, heart rate, respiratory rate, FiO_2, temperature, albumin, C-reactive protein, eGFR, pH, monocytes, WBC, frailty score, and Glasgow Coma Scale score. AUROC was 0.892 (95% CI 0.865-0.920) (Table 2). At a 20% predicted probability of ITU admission, sensitivity was 78% (95% CI 73-83), specificity was 83% (95% CI 82-85), positive predictive value was 50% (95% CI 46-54), and negative predictive was 95% (95% CI 93-96) (Table 3b). Calibration was good; a calibration plot is shown in Figure 1B, calibration slope was 0.89 (95% CI 0.77-1.02) (Supplementary Table 2). Model coefficients are shown in Supplementary Table 5.

Again, use of a missing category rather than imputed missing values improved model performance but was not selected as the final model due to potential problems with generalisability (AUROC 0.955, 95% CI 0.933-0.978). Models using continuous rather than categorical predictors (with splines where relevant; Supplementary Figure 2) gave a small improvement in AUROC (0.908, 95% CI 0.883-0.934), but were deemed less practical. Addition of comorbidities to the predictors included in the model did not improve performance.

Reduced UHB model and external validation in the CovidCollab dataset

A total of 6099 patients admitted with COVID-19 were included in the CovidCollab external validation dataset; 1668 (27%) died and 722 (12%) were admitted to ITU. Not all variables included in the UHB model derived above were available in the CovidCollab dataset. Therefore, revised and reduced models were developed in UHB data using the subset of candidate predictors common to both the UHB and CovidCollab datasets (reduced UHB dataset, UHB-R), using logistic regression with stepwise selection, and these were then externally validated in the CovidCollab dataset. The reduced set of 27 candidate predictors included: demographic characteristics: age, gender; symptoms: cough, fever, delirium; physiological measures and vital signs: BMI, systolic blood pressure, diastolic blood pressure, heart rate, temperature, respiratory rate, oxygen saturation, FiO_2, chest X-ray; frailty score; Glasgow Coma Scale score; laboratory test results: eGFR, pH, base excess, lymphocytes, neutrophil:lymphocyte ratio, haemoglobin, bicarbonate, C-reactive protein, alanine aminotransferase, urea, and lactate.

For peer review only - http://bmjopen.bmj.com/site/about/guidelines.xhtml

For the 28-day mortality outcome, following stepwise selection, the final 10 categorical predictors (common to both datasets) included in the logistic regression model were: age, oxygen saturation, FiO_2, respiratory rate, temperature, systolic blood pressure, C-reactive protein, pH, urea, and frailty score. These predictors were a subset of those in the original UHB model derivation, but gave similar model performance. AUROC in the UHB-R dataset was 0.791 (95% CI 0.761-0.822), and in the CovidCollab dataset was 0.767 (95% CI 0.754-0.780) (Table 2). At a 20% predicted probability of mortality, in the UHB-R dataset sensitivity was 86% (95% CI 85-88), specificity was 54% (95% CI 51-57), PPV was 42% (95% CI 40-43) and NPV was 91% (95% CI 90-92); in the CovidCollab dataset sensitivity was 88% (95% CI 87-89), specificity was 46% (95% CI 45-47), PPV was 38% (95% CI 0.37-0.38), and NPV was 91% (95% CI 91-92) (Table 4a). Calibration was good for both derivation and external validation datasets; calibration plots are shown in Figures 1C-D, calibration slopes were 0.86 (95% CI 0.82-0.90) and 0.88 (95% CI 0.80-0.96) for the UHB-R and CovidCollab datasets, respectively (Supplementary Table 2). Model coefficients are shown in Supplementary Table 6.

For the ITU admission outcome, the final 11 categorical predictors (common to both datasets) included in the model were: age, gender, fever, respiratory rate, FiO_2, C-reactive protein, eGFR, pH, neutrophil:lymphocyte ratio, frailty score, and Glasgow Coma Scale score. AUROC in the UHB-R dataset was 0.906 (95% CI 0.883-0.929), and in the CovidCollab dataset was 0.811 (95% CI 0.795-0.828) (Table 2). At a 20% predicted probability of ITU admission, in the UHB-R dataset sensitivity was 83% (95% CI 81-85), specificity was 83% (95% CI 82-84), PPV was 51% (95% CI 49-52) and NPV was 96% (95% CI 95-96); in the CovidCollab dataset sensitivity was 64% (95% CI 62-67), specificity was 80% (95% CI 79-82), PPV was 30% (95% CI29-32), and NPV was 94% (95% CI 94-95) (Table 4b). Calibration was good for both derivation and external validation datasets; calibration plots are shown in Figures 1E-F, calibration slopes were 0.94 (95% CI 0.84-1.03) and 0.93 (95% CI 0.84-1.02) for the UHB-R and CovidCollab datasets, respectively (Supplementary Table 2). Model coefficients are shown in Supplementary Table 7.

External validation of the ISARIC models in the UHB dataset

The recently published ISARIC model (XGBoost) was recalibrated to the UHB data and performance in the UHB data assessed for predicting mortality; AUROC was 0.757 (95% CI 0.721-0.792) (Table 2). For the simplified 4C score (logistic regression), the AUROC was marginally lower at 0.754 (95% CI 0.721-0.786).

It was not possible to externally validate the ISARIC model and 4C score in the CovidCollab dataset, as information on many of the comorbidities required to calculate the ISARIC comorbidity score was not available in the dataset.

Sensitivity analyses

Sensitivity analyses were carried out using the logistic regression models initially derived in the UHB dataset (18 predictors for mortality outcome and 16 predictors for ITU admission).

For peer review only - http://bmjopen.bmj.com/site/about/guidelines.xhtml

Complete case analysis

Few patients in the dataset had complete data (n = 224/1040, 22%); model performance in this patient subset was slightly poorer for mortality outcome: AUROC 0.690 (95% CI 0.588-0.791) for mortality and 0.887 (95% CI 0.825-0.949) for ITU admission. Including patients with missing variables, with missing values imputed, improved model performance; allowing even a single missing/imputed variable improved AUROC for mortality to 0.767 (95% CI 0.715-0.819) (Supplementary Table 8).

Stratification by gender

When patients were stratified by gender, the model predicting mortality performed slightly worse in males (AUROC 0.766, 95% CI 0.700-0.831) than females (0.788, 95% CI 0.729-0.847). The model predicting ITU admission performed similarly in males and females: 0.893 (95% CI 0.823-0.963) in males and 0.893 (95% CI 0.851-0.935) in females (Supplementary Table 8). Training models in gender-specific datasets offered no improvement on model performance (Supplementary Table 9).

Time series analysis

Supplementary Figure 3 shows variation in logistic regression coefficients for the candidate predictors from day of admission and up to 7 days later. The majority of coefficients remained relatively constant over time. However, several (not necessarily statistically significant) trends in the modification of effects over the week of admission on mortality were visible, such as a decrease over the week of the effect of obesity on mortality, elevated effect of eosinophils, and an increase over the week of the effect of elevated haemoglobin, elevated potassium and elevated oxygen saturation. Some of these might be depletion effects related to relatively high patient mortality in the first few days, for example, the apparent protective effect of obesity and high eosinophils.

Discussion

Using routinely collected data for more than a thousand patients admitted with COVID-19 at a large UK hospital trust, we have developed and externally validated prognostic models for mortality and ITU admission. The models showed good discrimination and calibration. The candidate predictors explored included a clinically informed, wider range of demographics, clinical observations, symptoms, comorbidities, biomarkers and radiological investigations than those included in the derivation of existing prognostic scores or models. If integrated into hospital electronic medical records systems, the model algorithms will provide a predicted probability of mortality or ITU admission within 28 days of hospital admission for each patient based on their individual data at, or close to, the time of admission, which will support clinicians' decision making with regard to appropriate patient care pathways and triage. This information might also assist clinicians in explaining complex prognostic assessments and decisions to patients and their relatives, particularly at a time when relatives are unable to see the patient and understand how unwell they are.

The models developed using all 63 available candidate predictors from UHB performed well with an optimism-adjusted AUROC of 0.778 (95 % CI 0.741-0.815) for mortality within 28

For peer review only - http://bmjopen.bmj.com/site/about/guidelines.xhtml

For peer review only

days of admission and 0.892 (95% CI 0.865-0.920) for ITU admission. Models performed well in male and female subgroups.

Not all variables included in the UHB dataset are routinely collected at admission in other hospitals; therefore, reduced models using only variables common to both UHB and the CovidCollab external validation dataset were explored. These were found to have slightly better discrimination, with an AUROC of 0.791 (95% CI 0.761-0.822) for mortality and 0.906 (95% CI 0.883-0.929) for ITU admission in the UHB derivation dataset. These reduced models also performed well in the CovidCollab external validation dataset, with AUROCs of 0.767 (95% CI 0.754-0.780) and 0.811 (95% CI 0.795-0.828) for mortality and ITU admission, respectively. Calibration of all models showed good agreement between observed and predicted probabilities, particularly at lower predicted probabilities in the range where the models would be of most clinical utility.

Two systematic reviews summarised the existing secondary care COVID-19 prognostic models or scores published until the 31st May 2020.[1,15] Many of the reported models (largely derived from Chinese cohorts) demonstrated high discriminatory performance, however all pre-existing models when assessed using the PROBAST score were at high risk of bias. Additionally, few models were externally validated in suitable cohorts. By deriving our model from routinely collected data, we were able to reduce the risk of bias in patient selection as well as predictor and outcome measurements. Additionally, in this study we were able to externally validate models in a large global heterogeneous cohort. Our study builds on the existing literature as the CovidCollab dataset includes some patients from low-middle income countries where validation studies of prediction models are yet to be published.

More recently, the most notable secondary care prediction model advised for uptake in UK hospitals was derived from the ISARIC-WHO collaborating cohort.[3] Both the full and reduced UHB-derived models for mortality had slightly better discrimination than the ISARIC model and 4C score in the UHB data (AUROC 0.757 (95% CI 0.724-0.791) for recalibrated ISARIC model and 0.754 (95% CI 0.721-0.786) for the simplified 4C score).This compares with AUROCs of 0.779 (0.772 to 0.785) for the ISARIC model and 0.767 (0.760 to 0.773) for the 4C score reported in the original ISARIC validation cohort.[3] Furthermore, the newly developed UHB model offers an advantage over the ISARIC and 4C models in that it utilises only routinely collected patient data recorded at admission, and does not require additional assessment and recording of specific comorbidities.

In our time series analysis we did not find strong evidence for trends in predictor coefficients over the first 8 days of admission, particularly for variables included in the final models, suggesting that time-dependent effects due to effect modification or selection bias in the first week are small. If models were implemented to treat time after admission in a fine-grained way, only a limited subset of biomarkers might be of interest. Another recent model derived from patients with COVID-19 in a Hong Kong hospital adopted the use of time-dependent routinely collected predictors; the model in the Hong Kong study demonstrated high discrimination, with an AUROC of 0.91 when predicting severe COVID-19 outcomes.[16] However, this model is yet to be externally validated.

For peer review only - http://bmjopen.bmj.com/site/about/guidelines.xhtml

Strengths and limitations

The UHB dataset represents one of the largest and most ethnically diverse patient cohorts within the UK. Additionally, as part of the early UHB response to the COVID-19 pandemic, the hospital trust ensured that, upon admission, all patients underwent a wide range of investigations to support international research efforts examining prognostic markers. This allowed us to examine a wide range of possible predictors (63 candidate predictors after exclusions). Lastly, a strength of this study was the good performance, in terms of both discrimination and calibration, of the simplified, reduced model in an externally validated cohort (CovidCollab), indicating its suitability for wider use, including potentially in LMICs.

Despite the strengths, the findings must be considered in light of the study's limitations. Although we were able to use a derivation dataset from UHB with low levels of missing data, the overall sample size was relatively small compared to that of the ISARIC study and was limited to one UK geographical location. However, we were able to externally validate the model in a larger external cohort. A second limitation was that in the external validation cohort we were unable to examine all of the predictors included in the original full UHB model due to only a reduced set of candidate predictors being available in CovidCollab. Nevertheless, the model performed well and the results suggest it may be applicable in a wide range of datasets where only a reduced set of predictor variables is available. It was not possible to carry out stratified analysis by ethnicity as, in the UHB dataset, too few patients were included in most of the strata; ethnicity data was not available in the CovidCollab dataset.

Conclusion

In this paper we have described the development and external validation of novel prognostic models which predict mortality and ITU admission within 28 days of admission for patients admitted to hospital with COVID-19, using routinely collected data gathered at admission. The simple, reduced models showed good discrimination and calibration, outperformed the existing ISARIC model and 4C score, and performed well in a validation cohort. The models can be integrated into existing electronic medical records systems to calculate each individual patient's probability of death or ITU admission at the time of hospital admission. The models should be further validated to determine their applicability in other populations. In addition, implementation of the models and clinical utility should be evaluated.

For peer review only - http://bmjopen.bmj.com/site/about/guidelines.xhtml

Figure legend

Figure 1. Calibration plots (observed probability (y-axis) against predicted probability (x-axis)): A. University Hospitals Birmingham (UHB) derivation dataset for mortality outcome; B. UHB derivation dataset for intensive therapy unit (ITU) admission outcome; C. UHB-R derivation/train dataset reduced model for mortality outcome; D. UHB-R derivation/train dataset reduced model for ITU admission outcome; E. CovidCollab external validation dataset reduced model for mortality outcome; F. CovidCollab external validation dataset reduced model for ITU admission outcome.

Ethical approval

Ethical approval was provided by the East Midlands – Derby REC (reference: 20/EM/0158) for the PIONEER Research Database (data from University Hospitals Birmingham).

For CovidCollab data, local, regional, and national approvals were obtained from all participating sites. In the UK, this study was registered as clinical audit or service evaluation, with approval granted in line with local information governance policies, in line with assessment and guidance by the Health Research Authority. At the lead site (University Hospitals Birmingham NHS Trust) this study was registered as clinical audit (CARMS-15986). In other countries, local principal investigators were responsible for obtaining approvals in line with their local, regional, and national guidelines and recommendations. Only routinely collected data was collected and patient care was not altered by this study. Anonymised data was securely transferred to the Birmingham Centre for Prospective and Observational Studies (BiCOPS), University of Birmingham via REDCap. All sites were required to confirm that approvals were in place prior to being provided with logins; written data sharing agreements were arranged where requested by individual sites.

Contributorship statement

The study was designed by NA, KN, ES, MP, YT, CS and TT. Data was collected by KG, ES, CS and SG. TT, NA and DG analysed the data. The first draft was written by NA and TT. All authors critically reviewed and revised the manuscript.

Funding

This work was funded by the Medical Research Council UK Research and Innovation (MRC UKRI) (reference COV0306). The funder had no role in developing the research question or the study protocol.

Competing interests

NJA, ES, KN, MP, AD, CS, TT and YT report a grant from UKRI MRC during the conduct of the study. ES reports grants from National Institute for Health Research (NIHR), Wellcome Trust, MRC, Health Data Research UK (HDR-UK), British Lung Foundation, and Alpha 1 Foundation

For peer review only - http://bmjopen.bmj.com/site/about/guidelines.xhtml

outside the submitted work. KN reports grants from MRC and HDR-UK outside the submitted work. DP reports grants from NIHR, MRC, and Chernakovsky Foundation outside the submitted work. All other authors have nothing to declare.

Transparency declaration

The lead author (the manuscript's guarantor) affirms that this manuscript is an honest, accurate, and transparent account of the study being reported; that no important aspects of the study have been omitted; and that any discrepancies from the study as planned (and, if relevant, registered) have been explained.

Exclusive licence

The Corresponding Author has the right to grant on behalf of all authors and does grant on behalf of all authors, a worldwide licence (http://www.bmj.com/sites/default/files/BMJ%20Author%20Licence%20March%202013.doc) to the Publishers and its licensees in perpetuity, in all forms, formats and media (whether known now or created in the future), to i) publish, reproduce, distribute, display and store the Contribution, ii) translate the Contribution into other languages, create adaptations, reprints, include within collections and create summaries, extracts and/or, abstracts of the Contribution and convert or allow conversion into any format including without limitation audio, iii) create any other derivative work(s) based in whole or part on the on the Contribution, iv) to exploit all subsidiary rights to exploit all subsidiary rights that currently exist or as may exist in the future in the Contribution, v) the inclusion of electronic links from the Contribution to third party material where-ever it may be located; and, vi) licence any third party to do any or all of the above.

Data sharing

No additional data available.

Dissemination declaration

Dissemination to participants is not possible as this is a review of existing evidence. Patients and the public will not be involved in dissemination of the findings.

Acknowledgements

MP and YT are supported by the NIHR Birmingham Biomedical Research Centre. The views expressed are those of the author(s) and not necessarily those of the NHS, NIHR or the Department of Health and Social Care.

We would also like to acknowledge the collaborators for the CovidCollab study: Sarah Richardson, Miles Witham, AGE Research Group, NIHR Newcastle Biomedical Research Centre, University of Newcastle and Newcastle-upon-Tyne Hospitals NHS Foundation Trust, UK; Omar A Abdelwahab, Elsayed M Awad, Ahmed Y Azzam, Ahmed Cordie, Ahmed O Elmehrath, Mostafa El-Shazly, Almontacer EB Masood, Alazher University Hospital, Egypt;

For peer review only - http://bmjopen.bmj.com/site/about/guidelines.xhtml

Osama MAS Abdulhadi, Hazem Ahmed, Muhammed Elhadi, Ahmed KM Hadreiez, Ahmed A Momen, Mosab AA Shaban, Alkhadra Hospital, Libya; Giuseppe Cecere, Aldo Rocca, Antonio Cardarelli Hospital, Italy; Hossam Aldein S Abd Elazeem, Mohammed H Abdelhafez, Islam A Ahmed, Shrouk M Elghazaly, Helal F Hetta, Mohamed Eltaher AA Ibrahim, Soha M Mohamed, Aliae AR Mohamed Hussein, Mohamed M Moustafa, Mariam Albatoul Nageh, Mahmoud M Saad, Alshaimaa M Saad, Omar Zein Elabedeen, Assiut University Hospital, Egypt; Victoria Cox, Danielle Hunsley, Rebecca Ryall, Kathleen T Shakespeare, Thyn Thyn, Rachael Webb, Barnsley Hospital NHS Foundation Trust, UK; Deepthy Hari Madhavan, Nik Sanyal, Birmingham Heartlands Hospital, UK; Bryony Brown, Matthew Hale, Bradford Teaching Hospitals NHS Foundation Trust, UK; Marie Goujon, Cambridge University Hospitals NHS Foundation Trust, UK; Benjamin Jelley, Cardiff University, UK; Laxmi Babar, Tina Doll, Agnieszka Felska, Daniel N Guerero, Sandeep Karthikeyan, Anne Karunatilleke, Helena Lee, Emma Livesey, Amelia Roberts, Charlotte Roberts-Rhodes, City Hospital, Birmingham, UK; Teresa Perra, Alberto Porcu, Cliniche San Pietro, A.O.U. Sassari, Italy; Antonio Buondonno, Giuseppe Cecere, Aldo Rocca, Department of Medicine and Health Science "V. Tiberio", University of Molise, Italy; Vesna Hogan, Iain Wilkinson, East Surrey Hospital, UK; Ioannis Baloyiannis, Jiannis Hajiioannou, Konstantinos Perivoliotis, George Tzovaras, General University Hospital of Larissa, Greece; Anna Fleck, Aine McGovern, Glasgow Royal Infirmary, UK; Victoria Gaunt, Gloucestershire Hospitals NHS Foundation Trust, UK; Laura Babb, Emily Bailey, Jay Darley, Ioan M Draghita, Alexander Hickman, Jason Kalloo, Akhil Kanzaria, Katy Madden, Wasim Nawaz, Ambreen Sadiq, Good Hope Hospital, UK; Rifa Cardoso, Margherita Faulkner, William Hurst, Ellen James, Aimee Leadbetter, Jordan Mayer, Tanya Robinson, Emma Stratton, Miriam Thake, Hannah Thould, Hannah Watson, Great Western Hospital, UK; Ravindra Belgamwar, Corrina Bentley, Harplands Hospital, UK; Sarah Freshwater, Health Education West Midlands, UK; Sergio DV Ruiz, Nuria M Sanz, Milagros Carrasco Prats, Pedro VF Fernández, Clara G Francés, Esther M Manuel, Miguel R Marin, Pedro L Morales, Patricia P Pérez, María V Soriano , Ismael Mora-Guzmán, Hospital General Reina Sofía, Spain; Ismael Mora-Guzmán, Hospital Santa Bárbara, Spain; Melanie Dani, Imperial College Healthcare NHS Trust, UK; Fabio Barra, Antonella Ferraiolo, Simone Ferrero, Claudio Gustavino, Chiara Kratochwila, IRCCS Ospedale Policlinico San Martino, Italy; Eric W Etchill, Alodia Gabre-Kidan, Joshua H Gray, Elliott R Haut, Harsha Malapati, Sarah F Rapaport, Kent A Stevens, Dominique Vervoort, Johns Hopkins Hospital, USA; Mohammed A Azab, King Abdullah Medical City Specialist Hospital, Saudi Arabia; Catherine Bryant, Hannah Cheney-Lowe, Catrin Cox, Andrew Crowe, Gordon Dick, Sarah Evans, Patrick CP Hogan, Kar Yee Law, Alexandra Richardson, Fabio Speranza, Kathryn Toppley, Julie Whitney, Eirene Yeung, King's College Hospital, UK; Mary Ni Lochlainn, Claire Steves, King's College London, UK; Alexandros Charalabopoulos, Spyridon Davakis, Amalia Karapanou, Theodore Liakakos, Eustratia Mpaili, Maria Mpoura, Michail A Sampanis, Nikolaos V Sipsas, Laiko University Hospital, Greece; Lucy Beishon, Elinor Burn, Parveen Doddamani, Victoria Haunton, Shahriar Kabir, Hannah Shaw, Chloe Warner, Leicester Royal Infirmary, UK; Chee Soo, Maidstone and Tunbridge Wells NHS Trust, UK; Yasmin K NasrEldin, Minia University Hospital, Egypt; Nourhan AA Ghannam, Minya General Hospital, Egypt; Isobel Sleeman, NHS Grampian, UK; Ravindra Belgamwar, Corrina Bentley, North Staffordshire Combined Healthcare NHS Trust, UK; Ali Ali, Sylvia Amini, James Belcher, Marie Giles, Hayley Jarvis, Nathan Jenko, Suvira Madan, Alexander Noar, Favour Nwolu, Jessica Parkin, Lauren C Passby, Jarita Sivam, Michael Surtees, Joanne Wagland, Ruth West, David Williams, Northern General Hospital, UK; Avinash Aujayeb, Lindsey Dew, Catherine Dotchin,

For peer review only - http://bmjopen.bmj.com/site/about/guidelines.xhtml

James M Dundas, Elinor Edwards, Georgia F Gilbert, Karl Jackson, Sarah H Manning, Dominic Maxfield, Nicholas Moss, Declan C Murphy, Ellen Tullo, Sarah H Welsh, Northumbria NHS Hospital Trust, UK; Tahir Masud, Nottingham University Hospitals NHS Trust, UK; Mustafa Alsahab, Oxford University Hospitals NHS Trust, UK; Antonio Buondonno, Enrico Pinotti, Policlinico San Pietro, Italy; Francesco Alessandri, Gioia Brachini, Giancarlo Ceccarelli, Flavia Ciccarone, Pierfranco M Cicerchia, Bruno Cirillo, Giorgio De Toma, Giulia Duranti, Enrico Fiori, Giovanni B Fonsi, Pierfrancesco Lapolla, Simona Meneghini, Andrea Mingoli, Francesco Pugliese, Paolo Sapienza, Luigi Simonelli, Martina Zambon, Policlinico Umberto I, Sapienza University of Rome, Italy; Caterina Cattel, Laurenny Guzman, Princess Royal Hospital, King's College Hospital Trust, Surrey, UK; Hannah Dowell, Aina Ibukunoluwakitan, Fawsiya Mohamed, Claire Spice, Amanda Stafford, Queen Alexandra Hospital, Portsmouth, UK; Jolene Atia, Catherine Atkin, Hannah Currie, Felicity Evison, Heena Khiroya, Zeinab Majid, Maria Qurashi, Queen Elizabeth Hospital Birmingham, UK; Siobhan Coulter, Claire McDonald, Georgina Muir, Catherine O'Mahony, Caroline Tait, Queen Elizabeth Hospital Gateshead, UK; Rowan Davies, Katie Honney, Laura Winter, Queen Elizabeth Hospital King's Lynn, UK; Olubayode Adewole, Queen's Hospital Romford, UK; Amir Abdelmalak, Mohammed Ahmad, Muhammed H Ansari, Kingsley Appiah, Rajesh Dwivedi, Hope Elrick, Hedra Ghobrial, Rosie Jackson, Sophie Jeffs, Sasha Jeyakumar, Eleanor Lunt, Bushra Muzammil, Sylvia Pytraczyk, Jonathan Sheldrake, Jennifer Smith, Hannah Tobiss, Mark Vettasseri, Ruth H Willott, Hein Zaw, Queens Medical Centre, Nottingham, UK; Katherine Patterson, Queen's University Belfast, UK; Moulinath Bannerjee, Jean Cummings, Barbara Hart, Tom Maughan, Royal Bolton Hospital, UK; Clare Baguneid, Gabrielle Budd, Lizzie Moriarty, Omoteniola Odutola, Hannah Street, Royal Derby Hospital, UK; Alexis Carr, Royal Devon and Exeter Hospital, UK; Jennifer Pigott, Royal Free London NHS Foundation Trust, UK; Sarah Baldwin, Hannah Bashir, Jake Gibbon, Amy Gray, Grace Lewis, Christina Page, Rosanna Varden, Royal Victoria Infirmary, UK; Anthony Grubb, Elizabeth Holmes, Harjinder Kainth, Natalie McNeela, Lara Reilly, Abigail Reynolds, Mark Whitsey, Royal Wolverhampton NHS Trust, UK; Mertcan Akcay, Yeşim Akdeniz, Emrah Akın, Fatih Altintoprak, Zülfü Bayhan, Recayi Capoglu, Hakan Demir, Necattin Firat, Emre Gonullu, Tarık Harmantepe, Baris Mantoglu, Ali Muhtaroglu, Merve Yigit, Yasin A Yildiz, Sakarya Faculty of Medicine, Turkey; Lobna Al-Sodani, Nicole Burden, Evelyn Charsley, Thomas Kneen, Angeline Price, Emma Swinnerton, Salford Royal Hospital, UK; Yen Nee J Bo, Hayley R Boden, Reem Bulla, Alison Eastaugh, Helena Lee, Asma Khan, Mohammed Mubin, Amelia Roberts, Anthony Umeadi, Stephanie Wallis, Megan Williamson, Yu Lelt Win, Sandwell General Hospital, UK; Eltayeb A Ahmed, Abdulmoiz Aljafari, Abdulmalek Aljafari, Abdulkader Mohammad, Sharq Alneel Hospital, Sudan; Manpreet Badh, Amy Birchenough, Nick Coulthard, Alice Devaney, Ratnam Gandhi, Katharine Hood, Samuel North, Martha Pinkney, Ellie Shaw, Elisha Whelan, Solihull Hospital, UK; Adam Seed, Southport and Ormskirk Hospital NHS Trust, UK; Gurinder Dogra, Claire Morris, Rebecca Wright, South Tyneside District Hospital, UK; Stephen Lim, Lia Orlando, Harnish Patel, Prabhleen Puri, Sing Yang Sim, Southampton General Hospital, UK; Carolyn Akladious, Gitanjali Amaratungaz, Taha Amir, Cheran Anandarajah, Rachael Anders, Sally Aziz, Anna Barnard, Monica Bawor, Laura Bremner, Hannah Bridgwater, Hejab Butt, Andra Caracostea, Theodore Chevallier, Victoria Comerford, Jack Cullen, Niamh Cunningham, Daniel Curley, Madeleine Daly, Nikhita Dattani, Benyamin Deldar, Arjun Desai, Nirali Desai, Jugdeep Dhesi, Maria Dias, Hannah C Dooley, Samiullah Dost, Hiren Dusara, Alexander Emery, Cassandra Fairhead, Antia Fernandez, Gracie Fisk, Madeleine Garner, Hannah Gerretsen, Andrew Ghobrial, Zaynub Ghufoor, Deirdre Green, Charlotte Greene,

For peer review only - http://bmjopen.bmj.com/site/about/guidelines.xhtml

Karla Griffith, Ayushi Gupta, Patrick Harrison, Aidan Haslam, Torben Heinsohn, Lindsay Hennah, Abigail Hobill, Katherine Hopkinson, Lara Howells, Nicole Hrouda, Irem Ishlek, Rishi Iyer, Nuha Kardaman, Mairead Kelly, Nicola I Kelly, Hesham Khalid, Muhammad S Khan, Haris Khan, Matthew King, Li Kok, Aneliya Kuzeva, Rebecca Lau, Gabriel Lee, Gavriella Levinson, Danielle Lis, Baguiasri Mandane, Jamie Mawhinney, Henry Maynard, Sophie Mclachlan, Michelle Metcalf, John Millwood-Hargrave, Kelvin Miu, Aaliya Mohammed, Hamilton Morrin, Stephanie Mulhern, Daniel Muller, Varun Nadkarni, Hanna Nguyen, Alice O'Docherty, Sinead O'Dwyer, Marc Osterdahl, Ismini Panayotidis, Shefali Patel, Rose Penfold, Rupini Perinpanathan, Dina Radenkovic, Thurkka Rajeswaran, Tahmina Razzak, Emily Ross-Skinner, Hazel Sanghvi, Ross Sayers, Luca Scott, Sri Sivarajan, Katharine Stambollouian, Jack Stewart, Amybel Taylor, Hrisheekesh Vaidya, Vittoria Vergani, Madiha Virk, Vaishali Vyas, Eleanor Watkins, Catherine Wilcock, Mettha Wimalasundera, Stephanie Worrall, Natalie Yeo, Humza Yusuf, St Thomas' Hospital, UK; Adam H Dyer, Cliona Ni Cheallaigh, Liam Townsend, St. James's Hospital, Ireland; Jocelyn Amer, Emily Lyon, Michael Sen, Sunderland Royal Hospital, UK; Mohammed Al-Sadawi, Adam Budzikoski, Ishmam Ibtida, Yusra Qaiser, SUNY Downstate Brooklyn, USA; Mohammad T Azam, Asad J Choudhry, William Marx, SUNY Upstate University Hospital, USA; Ahmad Bouhuwaish, Ahmed SA Taher, Tobruk Medical Center, Libya; Nikolaos Georgiou, Jade Man, Paul Reynolds, Benjaman To, Tunbridge Wells Hospital, UK; Fatma D Collins, Sharon Budd, Ellanna Griffin, Yue Guan, Deevia Hanji, Lily Lowes, Awolkhier Mohammedseid-Nurhussien, Farhana Moomo, Olebu Ogochukwu, Katie Thin, University Hospitals Coventry and Warwickshire NHS Trust, UK; Elinor Burn, University Hospitals of Leicester NHS Trust; Terry Hughes, Thomas A Jackson, Laura Magill, Lauren McCluskey, Hannah Moorey, Kelvin Okoth, Rita Perry, Michala Petitt, Thomas Pinkney, Daisy Wilson, University of Birmingham; Grace ME Pearson, University of Bristol, UK; Christopher N Osuafor, Kelli Torsney, University of Cambridge, UK; David Strain, Jane Masoli, University of Exeter, UK; Jenni Burton, Terence Quinn, University of Glasgow, UK; Lucy Beishon, University of Leicester, UK; Joanne Taylor, University of Manchester, UK; Adam Gordon, University of Nottingham, UK; Gilda De Paola, Gaetano Gallo, Giuseppe Sammarco, Giuseppina Vescio, University 'Magna Graecia' of Catanzaro, Italy; Shivam Pancholi, University of Nicosia, Cyprus; Natalie Cox, University of Southampton, UK; Rajni Lal, Western Sydney Local Health District, Australia; Rand A Hussein, Zafaraniyah General Hospital, Iraq.

For peer review only

Table 1. Baseline characteristics of participants admitted with COVID-19 in the derivation (University Hospitals Birmingham) and validation (CovidCollab) datasets

a. Demographic characteristics, comorbidities and symptoms

	Development cohort (UHB)			External validation cohort (CovidCollab)		
	Total	Alive	Died	Total	Alive	Died
N	1040	752	288	6099	4431	1668
Age category (years), n (%)						
<30	35 (3.4)	35 (4.7)	0 (0.0)	125 (2.0)	122 (2.8)	3 (0.2)
30-39	42 (4.0)	39 (5.2)	3 (1.0)	270 (4.4)	257 (5.8)	13 (0.8)
40-49	91 (8.8)	87 (11.6)	4 (1.4)	497 (8.1)	459 (10.4)	38 (2.3)
50-59	146 (14.0)	123 (16.4)	23 (8.0)	793 (13.0)	707 (16.0)	86 (5.2)
60-69	181 (17.4)	143 (19.0)	38 (13.2)	944 (15.5)	736 (16.6)	208 (12.5)
70-79	220 (21.2)	147 (19.5)	73 (25.3)	1325 (21.7)	891 (20.1)	434 (26.0)
80-89	214 (20.6)	124 (16.5)	90 (31.2)	1571 (25.8)	936 (21.1)	635 (38.1)
≥90	111 (10.7)	54 (7.2)	57 (19.8)	574 (9.4)	323 (7.3)	251 (15.0)
Gender (male), n (%)	589 (56.6)	423 (56.2)	166 (57.6)	3361 (55.1)	2342 (52.9)	1019 (61.1)
Ethnicity, n (%)				Not available		
White	590 (56.7)	406 (54.0)	184 (63.9)			
South Asian	127 (12.2)	95 (12.6)	32 (11.1)			
Black	68 (6.5)	46 (6.1)	22 (7.6)			
Other	255 (24.5)	205 (27.3)	50 (17.4)			
Comorbidities, n (%)						
Dementia	373 (35.9)	249 (33.1)	124 (43.1)	934 (15.3)	539 (12.2)	395 (23.7)
Cancer	135 (13.0)	90 (12.0)	45 (15.6)	649 (10.6)	409 (9.2)	240 (14.4)
Asthma	165 (15.9)	135 (18.0)	30 (10.4)	Not available		
Chronic obstructive pulmonary disease	283 (27.2)	211 (28.1)	72 (25.0)	Not available		
Sleep apnoea	49 (4.7)	36 (4.8)	13 (4.5)	1579 (25.9)	1125 (25.4)	454 (27.2)
Cardiovascular disease	567 (54.5)	366 (48.7)	201 (69.8)	3033 (49.7)	1977 (44.6)	1056 (63.3)
Hypertension	661 (63.6)	449 (59.7)	212 (73.6)	Not available		
Diabetes without complications	258 (24.8)	181 (24.1)	77 (26.7)	1794 (29.4)	1229 (27.7)	565 (33.9)
Diabetes with complications	112 (10.8)	76 (10.1)	36 (12.5)	Not available		
Peptic ulcer	29 (2.8)	25 (3.3)	4 (1.4)	Not available		
Liver disease	71 (6.8)	53 (7.0)	18 (6.2)	Not available		
Rheumatic/inflammatory disease	51 (4.9)	36 (4.8)	15 (5.2)	Not available		
Thyroid disorder	107 (10.3)	75 (10.0)	32 (11.1)	Not available		
ISARIC comorbidity score				Not applicable		
0	111 (10.7)	94 (12.5)	17 (5.9)			
1	234 (22.5)	175 (23.3)	59 (20.5)			
≥2	695 (66.8)	483 (64.2)	212 (73.6)			
Symptoms, n (%)						
Breathlessness	559 (59.2)	392 (56.6)	167 (66.3)	Not available		
Chest pain	39 (4.1)	33 (4.8)	6 (2.4)	Not available		
Cough	538 (57.0)	398 (57.5)	140 (55.6)	4259 (69.8)	3110 (70.2)	1149 (68.9)
Fever	465 (49.3)	339 (49.0)	126 (50.0)	3212 (52.7)	2394 (54.0)	818 (49.0)
Headache	42 (4.4)	37 (5.3)	5 (2.0)	Not available		
Malaise	186 (19.7)	147 (21.2)	39 (15.5)	Not available		
New onset diarrhoea or vomiting	56 (5.9)	49 (7.1)	7 (2.8)	Not available		
Sputum	84 (8.9)	53 (7.7)	31 (12.3)	Not available		
Delirium	80 (8.5)	41 (5.9)	39 (15.5)	1160 (20.1)	699 (16.7)	461 (28.8)
Outcomes						
Died within 28 days of admission	288 (27.7)	0 (0.0)	288 (100.0)	1668 (27.3)	0 (0.0)	1668 (100.0)
ITU admission within 28 days	183 (17.6)	132 (17.6)	51 (17.7)	722 (11.8)	477 (10.8)	245 (14.7)

For peer review only

b. Physiological measures and scores

	Development cohort (UHB)			External validation cohort (CovidCollab)		
	Total	Alive	Died	Total	Alive	Died
BMI category, kg/m^2						
Underweight (<18.5)	27 (2.6)	16 (2.1)	11 (3.8)	155 (2.5)	112 (2.5)	43 (2.6)
Normal weight (18.5-24.9)	273 (26.2)	179 (23.8)	94 (32.6)	1284 (21.1)	940 (21.2)	344 (20.6)
Overweight (25-29.9)	341 (32.8)	252 (33.5)	89 (30.9)	1247 (20.4)	999 (22.5)	248 (14.9)
Obese (>30)	366 (35.2)	280 (37.2)	86 (29.9)	1180 (19.3)	940 (21.2)	240 (14.4)
Missing	33 (3.2)	25 (3.3)	8 (2.8)	2233 (36.6)	1440 (32.5)	793 (47.5)
Systolic blood pressure (mm Hg), n (%)						
<140	688 (66.2)	503 (66.9)	185 (64.2)	4051 (66.4)	2936 (66.3)	1115 (66.8)
≥140	340 (32.7)	249 (33.1)	91 (31.6)	1926 (31.6)	1414 (31.9)	512 (30.7)
Missing	12 (1.2)	0 (0.0)	12 (4.2)	122 (2.0)	81 (1.8)	41 (2.5)
Diastolic blood pressure (mm Hg), n (%)						
<90	870 (83.7)	636 (84.6)	234 (81.2)	5075 (83.2)	3654 (82.5)	1421 (85.2)
≥90	158 (15.2)	116 (15.4)	42 (14.6)	910 (14.9)	701 (15.8)	209 (12.5)
Missing	12 (1.2)	0 (0.0)	12 (4.2)	114 (1.9)	76 (1.7)	38 (2.3)
Temperature (Celsius), n (%)						
<37.8	851 (81.8)	619 (82.3)	232 (80.6)	4164 (68.3)	3025 (68.3)	1139 (68.3)
≥37.8	187 (18.0)	133 (17.7)	54 (18.8)	1805 (29.6)	1316 (29.7)	489 (29.3)
Missing	2 (0.2)	0 (0.0)	2 (0.7)	130 (2.1)	90 (2.0)	40 (2.4)
Heart rate category (beats per minute), n (%)						
<80	288 (27.7)	211 (28.1)	77 (26.7)	1654 (27.1)	1190 (26.9)	464 (27.8)
80-99	441 (42.4)	326 (43.4)	115 (39.9)	2400 (39.4)	1794 (40.5)	606 (36.3)
≥100	309 (29.7)	215 (28.6)	94 (32.6)	1935 (31.7)	1370 (30.9)	565 (33.9)
Missing	2 (0.2)	0 (0.0)	2 (0.7)	110 (1.8)	77 (1.7)	33 (2.0)
Respirations (per minute), n (%)						
<20	450 (43.3)	363 (48.3)	87 (30.2)	2249 (36.9)	1769 (39.9)	480 (28.8)
≥20	573 (55.1)	389 (51.7)	184 (63.9)	3659 (60.0)	2522 (56.9)	1137 (68.2)
Missing	17 (1.6)	0 (0.0)	17 (5.9)	191 (3.1)	140 (3.2)	51 (3.1)
Oxygen saturation (%), n (%)						
<80	9 (0.9)	4 (0.5)	5 (1.7)	158 (2.6)	71 (1.6)	87 (5.2)
80-88	47 (4.5)	17 (2.3)	30 (10.4)	443 (7.3)	230 (5.2)	213 (12.8)
89-93	173 (16.6)	110 (14.6)	63 (21.9)	1108 (18.2)	775 (17.5)	333 (20.0)
≥94	809 (77.8)	621 (82.6)	188 (65.3)	4281 (70.2)	3284 (74.1)	997 (59.8)
Missing	2 (0.2)	0 (0.0)	2 (0.7)	109 (1.8)	71 (1.6)	38 (2.3)
Partial pressure of CO_2 (kPa), n (%)						
<4.67	184 (17.7)	125 (16.6)	59 (20.5)	947 (15.5)	627 (14.2)	320 (19.2)
4.67-6.3	380 (36.5)	278 (37.0)	102 (35.4)	650 (10.7)	467 (10.5)	183 (11.0)
≥6.4	176 (16.9)	124 (16.5)	52 (18.1)	214 (3.5)	128 (2.9)	86 (5.2)
Missing	300 (28.8)	225 (29.9)	75 (26.0)	4288 (70.3)	3209 (72.4)	1079 (64.7)
Portable oxygen concentrator fraction of inspired oxygen (%), n (%)						
≤0.28	629 (60.5)	458 (60.9)	171 (59.4)	3518 (57.7)	2730 (61.6)	788 (47.2)
0.28-0.49	56 (5.4)	35 (4.7)	21 (7.3)	1132 (18.6)	794 (17.9)	338 (20.3)
≥0.5	80 (7.7)	51 (6.8)	29 (10.1)	1003 (16.4)	541 (12.2)	462 (27.7)
Missing	275 (26.4)	208 (27.7)	67 (23.3)	446 (7.3)	366 (8.3)	80 (4.8)
Chest X-ray						
Clear/unchanged	210 (20.2)	161 (21.4)	49 (17.0)	1604 (26.3)	1225 (27.6)	379 (22.7)
Local consolidation	235 (22.6)	169 (22.5)	66 (22.9)	3226 (52.9)	2313 (52.2)	913 (54.7)
Ground glass opacity/bilateral infiltrates	393 (37.8)	273 (36.3)	120 (41.7)	637 (10.4)	402 (9.1)	235 (14.1)
Other/no firm diagnosis	99 (9.5)	65 (8.6)	34 (11.8)	-	-	-
None performed/missing	103 (9.9)	84 (11.2)	19 (6.6)	632 (10.4)	491 (11.1)	141 (8.5)
Frailty score, n (%)						
1-3	376 (36.2)	321 (42.7)	55 (19.1)	1451 (23.8)	1326 (29.9)	125 (7.5)

For peer review only - http://bmjopen.bmj.com/site/about/guidelines.xhtml

4-6	277 (26.6)	179 (23.8)	98 (34.0)	2079 (34.1)	1539 (34.7)	540 (32.4)
7-9	119 (11.4)	55 (7.3)	64 (22.2)	1911 (31.3)	1146 (25.9)	765 (45.9)
Missing	268 (25.8)	197 (26.2)	71 (24.7)	658 (10.8)	420 (9.5)	238 (14.3)
Glasgow Coma Scale score, n (%)						
<15	274 (26.3)	178 (23.7)	96 (33.3)	1314 (21.5)	744 (16.8)	570 (34.2)
15	222 (21.3)	186 (24.7)	36 (12.5)	4250 (69.7)	3366 (76.0)	884 (53.0)
Missing	544 (52.3)	388 (51.6)	156 (54.2)	535 (8.8)	321 (7.2)	214 (12.8)

For peer review only

For peer review only - http://bmjopen.bmj.com/site/about/guidelines.xhtml

c. Laboratory test results

	Development cohort (UHB)			External validation cohort (CovidCollab)		
	Total	Alive	Died	Total	Alive	Died
eGFR (ml/min), n (%)						
<30 (Stage 4 or above)	200 (19.2)	119 (15.8)	81 (28.1)	693 (11.4)	364 (8.2)	329 (19.7)
30-59 (Stage 3)	216 (20.8)	141 (18.8)	75 (26.0)	1362 (22.3)	846 (19.1)	516 (30.9)
60-89 (Stage 2)	317 (30.5)	246 (32.7)	71 (24.7)	1825 (29.9)	1393 (31.4)	432 (25.9)
>90 (Normal or high)	259 (24.9)	215 (28.6)	44 (15.3)	1818 (29.8)	1531 (34.6)	287 (17.2)
Missing	48 (4.6)	31 (4.1)	17 (5.9)	401 (6.6)	297 (6.7)	104 (6.2)
pH, n (%)						
<7.30	64 (6.2)	39 (5.2)	25 (8.7)	416 (6.8)	234 (5.3)	182 (10.9)
7.30-7.34	88 (8.5)	64 (8.5)	24 (8.3)	530 (8.7)	351 (7.9)	179 (10.7)
7.35-7.44	429 (41.2)	313 (41.6)	116 (40.3)	2300 (37.7)	1643 (37.1)	657 (39.4)
≥7.45	152 (14.6)	107 (14.2)	45 (15.6)	960 (15.7)	725 (16.4)	235 (14.1)
Missing	307 (29.5)	229 (30.5)	78 (27.1)	1893 (31.0)	1478 (33.4)	415 (24.9)
Base excess (mmol/l), n (%)						
<-2	202 (19.4)	125 (16.6)	77 (26.7)	981 (16.1)	560 (12.6)	421 (25.2)
-2-2	349 (33.6)	262 (34.8)	87 (30.2)	1996 (32.7)	1491 (33.6)	505 (30.3)
>2	182 (17.5)	136 (18.1)	46 (16.0)	1077 (17.7)	801 (18.1)	276 (16.5)
Missing	307 (29.5)	229 (30.5)	78 (27.1)	2045 (33.5)	1579 (35.6)	466 (27.9)
Anion gap (mmol/l), n (%)				Not available		
6-15	89 (8.6)	62 (8.2)	27 (9.4)			
≥16	579 (55.7)	409 (54.4)	170 (59.0)			
Missing	372 (35.8)	281 (37.4)	91 (31.6)			
White blood cell count (10^9/l), n (%)				Not available		
<3.9	79 (7.6)	69 (9.2)	10 (3.5)			
3.9-10.8	696 (66.9)	518 (68.9)	178 (61.8)			
≥10.9	215 (20.7)	135 (18.0)	80 (27.8)			
Missing	50 (4.8)	30 (4.0)	20 (6.9)			
Platelets (10^9/l), n (%)				Not available		
<150	179 (17.2)	121 (16.1)	58 (20.1)			
150-399	728 (70.0)	538 (71.5)	190 (66.0)			
≥400	80 (7.7)	62 (8.2)	18 (6.2)			
Missing	53 (5.1)	31 (4.1)	22 (7.6)			
Lymphocytes (10^9/l), n (%)						
<1.5	801 (77.0)	572 (76.1)	229 (79.5)	4684 (76.8)	3349 (75.6)	1335 (80.0)
≥1.5	195 (18.8)	154 (20.5)	41 (14.2)	1183 (19.4)	929 (21.0)	254 (15.2)
Missing	44 (4.2)	26 (3.5)	18 (6.2)	232 (3.8)	153 (3.5)	79 (4.7)
Neutrophil:lymphocyte ratio, n (%)						
<2.21	94 (9.0)	82 (10.9)	12 (4.2)	600 (9.8)	509 (11.5)	91 (5.5)
2.21-4.82	282 (27.1)	223 (29.7)	59 (20.5)	1635 (26.8)	1341 (30.3)	294 (17.6)
>4.82	620 (59.6)	421 (56.0)	199 (69.1)	3387 (55.5)	2265 (51.1)	1122 (67.3)
Missing	44 (4.2)	26 (3.5)	18 (6.2)	477 (7.8)	316 (7.1)	161 (9.7)
Mean corpuscular volume (fl), n (%)				Not available		
<80	91 (8.8)	69 (9.2)	22 (7.6)			
80-95	782 (75.2)	578 (76.9)	204 (70.8)			
≥96	123 (11.8)	79 (10.5)	44 (15.3)			
Missing	44 (4.2)	26 (3.5)	18 (6.2)			
Red cell distribution width (%), n (%)				Not available		
<11.5	13 (1.2)	11 (1.5)	2 (0.7)			
11.5-15.4	742 (71.3)	555 (73.8)	187 (64.9)			
≥15.5	240 (23.1)	160 (21.3)	80 (27.8)			
Missing	45 (4.3)	26 (3.5)	19 (6.6)			
Monocytes (10^9/l), n (%)				Not available		
<0.2	71 (6.8)	51 (6.8)	20 (6.9)			

For peer review only - http://bmjopen.bmj.com/site/about/guidelines.xhtml

For peer review only

0.2-0.8	731 (70.3)	539 (71.7)	192 (66.7)			
>0.8	194 (18.7)	136 (18.1)	58 (20.1)			
Missing	44 (4.2)	26 (3.5)	18 (6.2)			
Eosinophils (10^9/l), n (%)				Not available		
≤0.4	979 (94.1)	710 (94.4)	269 (93.4)			
>0.4	17 (1.6)	16 (2.1)	1 (0.3)			
Missing	44 (4.2)	26 (3.5)	18 (6.2)			
Haemoglobin (g/l), n (%)						
<115	310 (29.8)	215 (28.6)	95 (33.0)	1454 (23.8)	975 (22.0)	479 (28.7)
115-153	582 (56.0)	434 (57.7)	148 (51.4)	3718 (61.0)	2783 (62.8)	935 (56.1)
≥154	98 (9.4)	73 (9.7)	25 (8.7)	557 (9.1)	413 (9.3)	144 (8.6)
Missing	50 (4.8)	30 (4.0)	20 (6.9)	370 (6.1)	260 (5.9)	110 (6.6)
Glucose (mmol/l), n (%)				Not available		
<7.8	585 (56.2)	441 (58.6)	144 (50.0)			
7.8-8.4	76 (7.3)	47 (6.2)	29 (10.1)			
≥8.5	295 (28.4)	201 (26.7)	94 (32.6)			
Missing	84 (8.1)	63 (8.4)	21 (7.3)			
Bicarbonate (mmol/l), n (%)						
<22	184 (17.7)	118 (15.7)	66 (22.9)	996 (16.3)	608 (13.7)	388 (23.3)
22-28	451 (43.4)	339 (45.1)	112 (38.9)	2704 (44.3)	1981 (44.7)	723 (43.3)
≥29	98 (9.4)	66 (8.8)	32 (11.1)	437 (7.2)	320 (7.2)	117 (7.0)
Missing	307 (29.5)	229 (30.5)	78 (27.1)	1962 (32.2)	1522 (34.3)	440 (26.4)
C-reactive protein (mg/l), n (%)						
<10	84 (8.1)	76 (10.1)	8 (2.8)	690 (11.3)	616 (13.9)	74 (4.4)
10-99	406 (39.0)	321 (42.7)	85 (29.5)	2735 (44.8)	2101 (47.4)	634 (38.0)
≥100	483 (46.4)	314 (41.8)	169 (58.7)	2213 (36.3)	1381 (31.2)	832 (49.9)
Missing	67 (6.4)	41 (5.5)	26 (9.0)	461 (7.6)	333 (7.5)	128 (7.7)
Albumin (g/l), n (%)				Not available		
<25	189 (18.2)	123 (16.4)	66 (22.9)			
25-34	569 (54.7)	408 (54.3)	161 (55.9)			
≥35	214 (20.6)	176 (23.4)	38 (13.2)			
Missing	68 (6.5)	45 (6.0)	23 (8.0)			
Bilirubin (µmol/l), n (%)						
<21	822 (79.0)	604 (80.3)	218 (75.7)	Not available		
≥21	151 (14.5)	104 (13.8)	47 (16.3)			
Missing	67 (6.4)	44 (5.9)	23 (8.0)			
Alanine aminotransferase (U/l), n (%)						
<55	837 (80.5)	601 (79.9)	236 (81.9)	4126 (67.7)	2986 (67.4)	1140 (68.3)
≥55	134 (12.9)	106 (14.1)	28 (9.7)	777 (12.7)	559 (12.6)	218 (13.1)
Missing	69 (6.6)	45 (6.0)	24 (8.3)	1196 (19.6)	886 (20.0)	310 (18.6)
Alkaline phosphatase (U/l), n (%)				Not available		
<130	814 (78.3)	605 (80.5)	209 (72.6)			
≥130	159 (15.3)	103 (13.7)	56 (19.4)			
Missing	67 (6.4)	44 (5.9)	23 (8.0)			
Urea (mmol/l), n (%)						
<7.8	567 (54.5)	466 (62.0)	101 (35.1)	2585 (42.4)	2122 (47.9)	463 (27.8)
≥7.8	429 (41.2)	259 (34.4)	170 (59.0)	2409 (39.5)	1399 (31.6)	1010 (60.6)
Missing	44 (4.2)	27 (3.6)	17 (5.9)	1105 (18.1)	910 (20.5)	195 (11.7)
Potassium (mmol/l), n (%)				Not available		
2.5-5.2	868 (83.5)	644 (85.6)	224 (77.8)			
≥5.3	49 (4.7)	29 (3.9)	20 (6.9)			
Missing	123 (11.8)	79 (10.5)	44 (15.3)			
Sodium (mmol/l), n (%)				Not available		
<133	146 (14.0)	105 (14.0)	41 (14.2)			
133-144	746 (71.7)	567 (75.4)	179 (62.2)			
≥145	104 (10.0)	53 (7.0)	51 (17.7)			
Missing	44 (4.2)	27 (3.6)	17 (5.9)			

For peer review only

For peer review only - http://bmjopen.bmj.com/site/about/guidelines.xhtml

Corrected calcium (mmol/l), n (%)				Not available		
<2.2	142 (13.7)	99 (13.2)	43 (14.9)			
2.2-2.5	767 (73.8)	577 (76.7)	190 (66.0)			
≥2.6	38 (3.7)	17 (2.3)	21 (7.3)			
Missing	93 (8.9)	59 (7.8)	34 (11.8)			
Lactate (U/l), n (%)						
≤2.2	490 (47.1)	362 (48.1)	128 (44.4)	3091 (50.7)	2327 (52.5)	764 (45.8)
>2.2	192 (18.5)	121 (16.1)	71 (24.7)	996 (16.3)	558 (12.6)	438 (26.3)
Missing	358 (34.4)	269 (35.8)	89 (30.9)	2012 (33.0)	1546 (34.9)	466 (27.9)
Haematocrit (l/l), n (%)				Not available		
<0.5	963 (92.6)	707 (94.0)	256 (88.9)			
≥0.5	33 (3.2)	19 (2.5)	14 (4.9)			
Missing	44 (4.2)	26 (3.5)	18 (6.2)			

Table 2. Area under the receiver operating characteristic curve (AUROC) for models developed in University Hospitals Birmingham (UHB) data and externally validated in CovidCollab data, and for external validation of the ISARIC model and 4C score

Dataset	Outcome	AUROC (95% CI)
**Model development***		
UHB	Mortality	0.778 (0.741-0.815)
UHB	ITU admission	0.892 (0.865-0.920)
UHB-R†	Mortality	0.791 (0.761-0.822)
UHB-R†	ITU admission	0.906 (0.883-0.929)
**External validation of new model***		
CovidCollab	Mortality	0.767 (0.754-0.780)
CovidCollab	ITU admission	0.811 (0.795-0.828)
**External validation of ISARIC and 4C models in UHB data**		
UHB – ISARIC model (XGBoost)	Mortality	0.757 (0.721-0.792)
UHB – 4C score	Mortality	0.754 (0.721-0.786)

*Models derived using logistic regression with stepwise selection of candidate predictors and categorisation of continuous variables into clinically meaningful categories (after imputing missing data).

†Not all variables included in the full University Hospital Birmingham (UHB) model were available in the CovidCollab dataset. Therefore, revised (reduced) models were developed in UHB data using a subset of the candidate predictors common to both the UHB and CovidCollab datasets (UHB-R), and these were then externally validated in the CovidCollab dataset.

For peer review only

Table 3a. Sensitivity, specificity, positive predictive value (PPV) and negative predictive value (NPV) for mortality at 28 days after admission (University Hospitals Birmingham derivation dataset)

Predicted probability	TP	TN	FP	FN	Sensitivity (95% CI)	Specificity (95% CI)	PPV (95% CI)	NPV (95% CI)
10%	271	277	475	17	0.941 (0.915-0.967)	0.369 (0.355-0.382)	0.363 (0.354-0.373)	0.942 (0.917-0.968)
20%	236	441	311	52	0.821 (0.792-0.850)	0.587 (0.563-0.611)	0.432 (0.412-0.452)	0.895 (0.878-0.912)
30%	194	544	208	94	0.673 (0.636-0.710)	0.723 (0.703-0.743)	0.482 (0.452-0.512)	0.852 (0.836-0.869)
40%	152	626	126	136	0.527 (0.492-0.562)	0.833 (0.815-0.850)	0.547 (0.505-0.589)	0.821 (0.807-0.835)
50%	118	680	72	170	0.410 (0.378-0.443)	0.904 (0.888-0.919)	0.620 (0.568-0.672)	0.800 (0.789-0.811)
60%	82	713	39	206	0.284 (0.231-0.337)	0.948 (0.944-0.952)	0.675 (0.631-0.719)	0.776 (0.763-0.788)
70%	50	728	24	238	0.174 (0.151-0.197)	0.968 (0.967-0.969)	0.673 (0.644-0.703)	0.754 (0.748-0.759)
80%	24	741	11	264	0.082 (0.062-0.102)	0.985 (0.978-0.993)	0.685 (0.568-0.801)	0.737 (0.733-0.741)
90%	8	750	2	280	0.027 (0.015-0.039)	0.998 (0.995-1)	0.808 (0.573-1)	0.728 (0.725-0.731)

TP = true positives, TN = true negatives, FP = false positives, FN = false negatives.

For peer review only

For peer review only - http://bmjopen.bmj.com/site/about/guidelines.xhtml

Table 3b. Sensitivity, specificity, positive predictive value (PPV) and negative predictive value (NPV) for intensive therapy unit admission within 28 days after admission (University Hospitals Birmingham derivation dataset)

Predicted probability	TP	TN	FP	FN	Sensitivity (95% CI)	Specificity (95% CI)	PPV (95% CI)	NPV (95% CI)
10%	160	618	239	23	0.877 (0.839-0.914)	0.721 (0.694-0.748)	0.402 (0.377-0.426)	0.965 (0.954-0.975)
20%	143	714	143	40	0.779 (0.726-0.833)	0.834 (0.816-0.851)	0.500 (0.457-0.543)	0.946 (0.933-0.960)
30%	127	769	88	56	0.695 (0.655-0.735)	0.898 (0.888-0.908)	0.592 (0.557-0.627)	0.932 (0.924-0.941)
40%	113	798	59	70	0.620 (0.577-0.663)	0.931 (0.922-0.940)	0.659 (0.616-0.701)	0.920 (0.911-0.929)
50%	94	817	40	89	0.513 (0.472-0.553)	0.953 (0.944-0.963)	0.701 (0.648-0.754)	0.902 (0.894-0.909)
60%	79	832	25	104	0.430 (0.378-0.481)	0.970 (0.966-0.975)	0.755 (0.716-0.795)	0.888 (0.879-0.898)
70%	67	840	17	116	0.366 (0.327-0.405)	0.981 (0.972-0.989)	0.802 (0.742-0.863)	0.879 (0.872-0.885)
80%	53	848	9	130	0.292 (0.255-0.329)	0.990 (0.987-0.992)	0.859 (0.839-0.879)	0.867 (0.862-0.873)
90%	34	854	3	149	0.188 (0.146-0.230)	0.997 (0.995-0.999)	0.924 (0.875-0.974)	0.852 (0.845-0.858)

TP = true positives, TN = true negatives, FP = false positives, FN = false negatives.

For peer review only

For peer review only - http://bmjopen.bmj.com/site/about/guidelines.xhtml

Table 4a. Sensitivity, specificity, positive predictive value (PPV) and negative predictive value (NPV) for mortality at 28 days after admission (University Hospitals Birmingham derivation dataset and CovidCollab external validation dataset, using predictors common to both datasets)

Predicted probability	TP	TN	FP	FN	Sensitivity (95% CI)	Specificity (95% CI)	PPV (95% CI)	NPV (95% CI)
**UHB-R derivation dataset (reduced model)**								
10%	278	249	503	10	0.964 (0.955-0.972)	0.332 (0.315-0.348)	0.356 (0.351-0.360)	0.960 (0.952-0.968)
20%	248	406	346	40	0.862 (0.847-0.878)	0.539 (0.510-0.569)	0.418 (0.403-0.433)	0.911 (0.902-0.920)
30%	202	536	216	86	0.703 (0.671-0.734)	0.713 (0.698-0.727)	0.484 (0.464-0.504)	0.862 (0.849-0.876)
40%	153	635	117	135	0.532 (0.511-0.553)	0.844 (0.831-0.858)	0.567 (0.547-0.587)	0.825 (0.819-0.831)
50%	104	686	66	184	0.360 (0.335-0.384)	0.913 (0.905-0.920)	0.612 (0.593-0.632)	0.788 (0.782-0.794)
60%	66	723	29	222	0.228 (0.211-0.246)	0.962 (0.953-0.970)	0.696 (0.663-0.729)	0.765 (0.762-0.768)
70%	35	744	8	253	0.122 (0.101-0.144)	0.990 (0.985-0.994)	0.819 (0.753-0.884)	0.746 (0.742-0.751)
80%	14	750	2	274	0.050 (0.036-0.064)	0.997 (0.996-0.998)	0.868 (0.835-0.900)	0.733 (0.730-0.735)
90%	3	752	0	285	0.010 (0-0.022)	0.999 (0.998-1)	0.900 (0.573-1)	0.725 (0.723-0.727)
**CovidCollab external validation dataset**								
10%	1614	1218	3213	54	0.967 (0.962-0.973)	0.275 (0.260-0.290)	0.334 (0.330-0.338)	0.957 (0.951-0.963)
20%	1471	2028	2403	197	0.882 (0.874-0.889)	0.458 (0.445-0.471)	0.380 (0.375-0.384)	0.911 (0.907-0.916)
30%	1289	2721	1710	379	0.773 (0.763-0.782)	0.614 (0.602-0.626)	0.430 (0.425-0.435)	0.878 (0.874-0.881)
40%	1059	3264	1167	609	0.635 (0.605-0.665)	0.737 (0.717-0.756)	0.476 (0.469-0.483)	0.843 (0.835-0.851)
50%	850	3698	733	818	0.510 (0.470-0.549)	0.835 (0.817-0.852)	0.537 (0.523-0.551)	0.819 (0.810-0.828)
60%	599	4025	406	1069	0.359 (0.305-0.414)	0.908 (0.890-0.927)	0.596 (0.578-0.615)	0.790 (0.779-0.801)
70%	380	4222	209	1288	0.228 (0.186-0.270)	0.953 (0.940-0.965)	0.646 (0.626-0.666)	0.766 (0.759-0.774)
80%	174	4355	76	1494	0.104 (0.077-0.132)	0.983 (0.978-0.987)	0.697 (0.671-0.722)	0.745 (0.740-0.750)
90%	37	4414	17	1631	0.022 (0.006-0.039)	0.996 (0.995-0.998)	0.676 (0.572-0.780)	0.730 (0.727-0.733)

TP = true positives, TN = true negatives, FP = false positives, FN = false negatives.

For peer review only - http://bmjopen.bmj.com/site/about/guidelines.xhtml

Table 4b. Sensitivity, specificity, positive predictive value (PPV) and negative predictive value (NPV) for intensive therapy unit admission within 28 days after admission (University Hospitals Birmingham derivation dataset and CovidCollab external validation dataset, using predictors common to both datasets)

Predicted probability	TP	TN	FP	FN	Sensitivity (95% CI)	Specificity (95% CI)	PPV (95% CI)	NPV (95% CI)
**UHB-R derivation dataset (reduced model)**								
10%	165	590	267	18	0.904 (0.877-0.931)	0.689 (0.671-0.707)	0.383 (0.368-0.398)	0.971 (0.963-0.979)
20%	152	709	148	31	0.831 (0.812-0.849)	0.827 (0.816-0.839)	0.507 (0.494-0.519)	0.958 (0.954-0.962)
30%	132	765	92	51	0.723 (0.698-0.749)	0.893 (0.882-0.904)	0.591 (0.567-0.615)	0.938 (0.933-0.943)
40%	112	803	54	71	0.613 (0.573-0.653)	0.937 (0.928-0.946)	0.675 (0.649-0.702)	0.919 (0.912-0.926)
50%	92	829	28	91	0.505 (0.483-0.526)	0.967 (0.956-0.979)	0.768 (0.708-0.829)	0.901 (0.898-0.905)
60%	77	841	16	106	0.419 (0.403-0.435)	0.981 (0.974-0.988)	0.828 (0.779-0.877)	0.888 (0.886-0.890)
70%	67	847	10	116	0.365 (0.344-0.386)	0.988 (0.983-0.994)	0.870 (0.816-0.924)	0.879 (0.876-0.883)
80%	46	851	6	137	0.251 (0.189-0.313)	0.993 (0.989-0.997)	0.889 (0.840-0.938)	0.861 (0.852-0.871)
90%	20	855	2	163	0.108 (0.067-0.149)	0.997 (0.996-0.999)	0.898 (0.838-0.957)	0.840 (0.833-0.846)
**CovidCollab external validation dataset**								
10%	574	3623	1754	148	0.794 (0.764-0.825)	0.674 (0.651-0.697)	0.247 (0.238-0.255)	0.961 (0.956-0.965)
20%	465	4312	1065	257	0.644 (0.621-0.667)	0.802 (0.785-0.819)	0.304 (0.288-0.321)	0.944 (0.941-0.947)
30%	377	4655	722	345	0.523 (0.497-0.549)	0.866 (0.847-0.885)	0.344 (0.320-0.367)	0.931 (0.929-0.933)
40%	315	4881	496	407	0.437 (0.381-0.492)	0.908 (0.887-0.928)	0.389 (0.361-0.417)	0.923 (0.918-0.929)
50%	250	5045	332	472	0.346 (0.258-0.434)	0.938 (0.920-0.956)	0.430 (0.413-0.447)	0.914 (0.905-0.923)
60%	178	5168	209	544	0.247 (0.175-0.318)	0.961 (0.945-0.977)	0.462 (0.420-0.503)	0.905 (0.898-0.912)
70%	113	5256	121	609	0.157 (0.105-0.208)	0.978 (0.967-0.988)	0.486 (0.439-0.534)	0.896 (0.891-0.901)
80%	57	5319	58	665	0.079 (0.044-0.114)	0.989 (0.984-0.995)	0.500 (0.410-0.590)	0.889 (0.886-0.892)
90%	18	5357	20	704	0.025 (0.006-0.044)	0.996 (0.993-1)	0.485 (0.367-0.603)	0.884 (0.882-0.886)

TP = true positives, TN = true negatives, FP = false positives, FN = false negatives.

For peer review only

References

[1] Wynants L, Van Calster B, Bonten MMJ, et al. Prediction models for diagnosis and prognosis of covid-19 infection: systematic review and critical appraisal. BMJ 2020;369:m1328. doi:10.1136 bmj.m1328

[2] Liang W, et al. Development and Validation of a Clinical Risk Score to Predict the Occurrence of Critical Illness in Hospitalized Patients with COVID-19. JAMA Intern Med 2020. doi:10.1001/jamainternmed.2020.2033

[3] Knight SR, Ho A, Pius R et al. Risk stratification of patients admitted to hospital with covid-19 using the ISARIC WHO Clinical Characterisation Protocol: development and validation of the 4C Mortality Score. BMJ 2020;370:m3339 doi:10.1136/bmj.m3339

[4] Hua-Gen Li M, Hutchinson A, Tacey M, Duke G. Reliability of comorbidity scores derived from administrative data in the tertiary hospital intensive care setting: a cross-sectional study BMJ Health & Care Informatics 2019;26:e000016. doi:10.1136/bmjhci-2019-000016

[5] Reynolds A, Seed A, Khan A, et al. on behalf of NIHR Clinical Research Network West Midlands, Birmingham Surgical Trials Consortium, and Geriatric Medicine Research Collaborative. CovidCollab. A panspecialty international project to determine point of care predictors from routinely collected data. Protocol v 1.4. 2020. Available at: https://www.bgs.org.uk/sites/default/files/content/attachment/2020-04-03/CovidCollab%20Main%20Study%20Protocol%20v2.pdf [accessed 26th Oct 2020].

[6] Geriatric Medicine Research Collaborative, Covid Collaborative, Carly Welch. Age and Frailty Are Independently Associated with Increased Mortality in COVID-19: Results of an International Multi-Centre Study. Preprint available at: https://ssrn.com/abstract=3709847, doi:10.2139/ssrn.3709847 [accessed 9th December 2020]

[7] Zhang Z. Variable selection with stepwise and best subset approaches. Ann Transl Med 2016;4(7):136. doi:10.21037/atm.2016.03.35

[8] Freund Y, Schapire RE. A decision-theoretic generalization of on-line learning and an application to boosting. Journal of Computer and System Sciences 1997;55(1):119-139.

[9] Mandrekar JN. Receiver operating characteristic curve in diagnostic test assessment. J Thorac Oncol 2010;5:1315-6. doi:10.1097/JTO.0b013e3181ec173d

[10] Rubin DB (ed.). Multiple imputation for nonresponse in surveys. 1987. doi:10.1002/9780470316696

[11] White IR, Royston P, Wood AM. Multiple imputation using chained equations: Issues and guidance for practice. Statistics in Medicine 2011;30:377-399.

[12] Rockwood K, Song X, MacKnight C, Bergman H, Hogan DB, McDowell I, Mitnitski A. A global clinical measure of fitness and frailty in elderly people. CMAJ. 2005;173(5):489-95. doi:10.1503/cmaj.050051

[13] Teasdale G, Jennett B. Assessment and prognosis of coma after head injury. Acta Neurochirurgica 1976;34:45-55. doi:10.1007/BF01405862

[14] Institute of Neurological Sciences NHS Greater Glasgow and Clyde. Glasgow Coma Scale: Do it this way. 2015. Royal College of Physicians and Surgeons of Glasgow: Glasgow. Available at: https://glasgowcomascale.org/downloads/GCS-Assessment-Aid-English.pdf?v=3 [accessed 9th December 2020].

[15] Katzenschlager S, Zimmer AJ, Gottschalk C, et al. Can we predict the severe course of COVID-19 – a systematic review and meta-analysis of indicators of clinical outcome? medRxiv preprint 2020. doi:10.1101/2020.11.09.20228858

[16] Zhou J, Lee S, Wang X, et al. Development of a predictive risk model for severe COVID-19 disease using population-based administrative data. medRxiv preprint 2020. doi:10.1101/2020.10.21.20217380

For peer review only - http://bmjopen.bmj.com/site/about/guidelines.xhtml

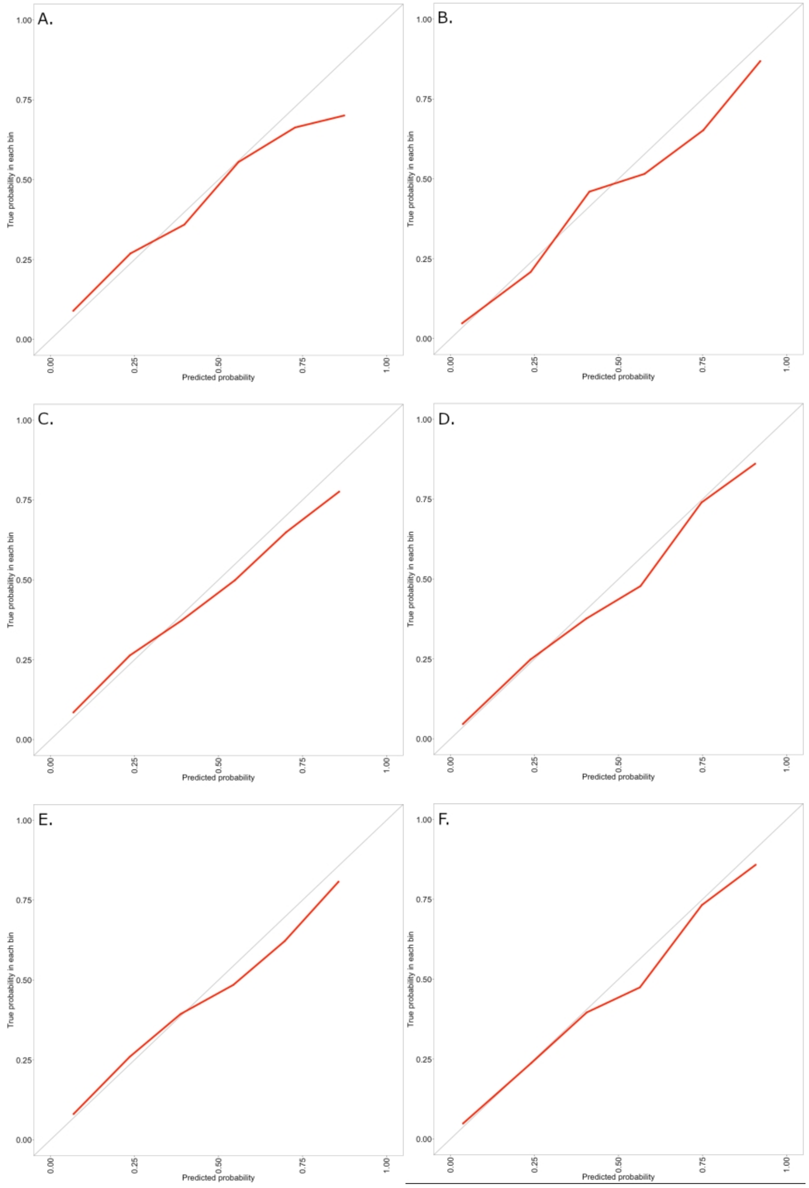

169x254mm (300 x 300 DPI)

Supplementary Table 1. Area under the receiver operating characteristic curve (AUROC) for each of the models assessed in the derivation dataset (University Hospitals Birmingham) for predicting death within 28 days of hospital admission

Model	Continuous data	Missing continuous data	Candidate predictors included	AUROC (95% CI)
Logistic	Categorical	Imputed	All	0.744 (0.709-0.780)
LASSO	Categorical	Imputed	All	0.751 (0.716-0.786)
GBM	Categorical	Imputed	All	0.756 (0.721-0.790)
Logistic	Categorical	Imputed	Stepwise selection	0.778 (0.741-0.815)
Logistic	Categorical	Imputed	All plus comorbidities	0.734 (0.697-0.771)
Logistic	Categorical	Imputed	Demographic only	0.709 (0.674-0.744)
Logistic	Categorical	Missing category	All	0.766 (0.725-0.808)
Logistic	Categorical	Missing category	Demographic only	0.705 (0.670-0.740)
Logistic	Categorical	Missing category	Stepwise selection	0.805 (0.777-0.834)
LASSO	Categorical	Missing category	All	0.781 (0.745-0.817)
GBM	Categorical	Missing category	All	0.776 (0.739-0.813)
Logistic	Continuous	Imputed	All	0.765 (0.724-0.806)
Logistic	Continuous	Imputed	All with splines*	0.719 (0.633-0.805)
LASSO	Continuous	Imputed	All	0.777 (0.745-0.808)
LASSO	Continuous	Imputed	All with splines*	0.782 (0.752-0.812)
GBM	Continuous	Imputed	All	0.773 (0.741-0.804)
GBM	Continuous	Imputed	All with splines*	0.773 (0.739-0.808)
Logistic	Continuous	Imputed	Stepwise selection	0.794 (0.764-0.823)
Logistic	Continuous	Imputed	Stepwise selection with splines*	0.791 (0.759-0.823)

All = all demographic, symptoms, vital signs/physiological measures, and laboratory test result candidate predictors. *Splines were used to model continuous variables where non-liner associations with the outcome were observed. LASSO = least absolute shrinkage and selection operator; GBM = gradient boosting machine.

For peer review only

Supplementary Table 2. Calibration slopes and intercepts for model performance in the University Hospitals Birmingham (UHB) derivation datasets (full (UHB) and reduced (UHB-R) datasets) and in the CovidCollab external validation dataset

Dataset	Outcome	Calibration	
		Slope	Intercept
**Model development***			
UHB	Mortality	0.78 (0.66-0.91)	0.06 (0.00-0.13)
UHB	ITU admission	0.89 (0.77-1.02)	0.02 (-0.05-0.09)
UHB-R†	Mortality	0.86 (0.82-0.90)	0.04 (0.02-0.06)
UHB-R†	ITU admission	0.94 (0.84-1.03)	0.01 (-0.05-0.06)
**External validation of new model**			
CovidCollab	Mortality	0.88 (0.80-0.96)	0.03 (-0.01-0.07)
CovidCollab	ITU admission	0.93 (0.84-1.02)	0.01 (-0.04-0.06)

*Models derived using logistic regression with stepwise selection of candidate predictors and categorisation of continuous variables into clinically meaningful categories (after imputing missing data).

†Not all variables included in the full University Hospital Birmingham (UHB) model were available in the CovidCollab dataset. Therefore, revised (reduced) models were developed in UHB data using a subset of the candidate predictors common to both the UHB and CovidCollab datasets (UHB-R), and these were then externally validated in the CovidCollab dataset.

For peer review only

Supplementary Table 3. Logistic regression model coefficients for mortality within 28 days of admission in the derivation dataset (University Hospitals Birmingham)

Predictor	Coefficient
Intercept	-16.312
Age category (years), n (%)	
<30	Ref
30-39	13.508
40-49	12.722
50-59	14.158
60-69	14.350
70-79	15.160
80-89	15.570
≥90	15.864
Breathlessness	0.304
Sputum	0.442
Temperature (Celsius), n (%)	
<37.8	Ref
≥37.8	0.339
Systolic blood pressure (mm Hg), n (%)	
<140	Ref
≥140	-0.360
Respirations (per minute), n (%)	
<20	Ref
≥20	0.358
Oxygen saturation (%), n (%)	
<80	Ref
80-88	0.319
89-93	-0.808
≥94	-1.135
Portable oxygen concentrator fraction of inspired oxygen (%), n (%)	
≤0.28	Ref
0.28-0.49	0.518
≥0.5	0.627
pH, n (%)	
<7.30	Ref
7.30-7.34	-1.017
7.35-7.44	-0.486
≥7.45	-0.406
White blood cell count ($10^{!}$/l), n (%)	
<3.9	Ref
3.9-10.8	0.915
≥10.9	0.999
Platelets ($10^{!}$/l), n (%)	
<150	Ref

For peer review only

150-399	-0.507
≥400	-0.792
C-reactive protein (mg/l), n (%)	
<10	Ref
10-99	0.530
≥100	1.014
Glucose (mmol/l), n (%)	
<7.8	Ref
7.8-8.4	0.571
≥8.5	0.426
Alkaline phosphatase (U/l), n (%)	
<130	Ref
≥130	0.389
Urea (mmol/l), n (%)	
<7.8	Ref
≥7.8	0.376
Corrected calcium (mmol/l), n (%)	
<2.2	Ref
2.2-2.5	-0.695
≥2.6	0.503
Eosinophils ($10^{!}$/l), n (%)	
≤0.4	Ref
>0.4	-1.408
Frailty score, n (%)	
1-3	
4-6	0.170
7-9	0.722

(Coefficients derived after a single imputation of missing data.)

For peer review only

Supplementary Table 4. Area under the receiver operating characteristic curve (AUROC) for each of the models assessed in the derivation dataset (University Hospitals Birmingham) for predicting intensive therapy unit admission within 28 days of hospital admission

Model	Continuous data	Missing continuous data	Candidate predictors included	AUROC (95% CI)
Logistic	Categorical	Imputed	All	0.846 (0.813-0.880)
LASSO	Categorical	Imputed	All	0.872 (0.842-0.901)
GBM	Categorical	Imputed	All	0.875 (0.843-0.906)
Logistic	Categorical	Imputed	Stepwise selection	0.892 (0.865-0.920)
Logistic	Categorical	Imputed	All plus comorbidities	0.870 (0.836-0.904)
Logistic	Categorical	Imputed	Demographic only	0.763 (0.728-0.798)
Logistic	Categorical	Missing category	All	0.911 (0.867-0.954)
Logistic	Categorical	Missing category	Demographic only	0.767 (0.734-0.800)
Logistic	Categorical	Missing category	Stepwise selection	0.955 (0.933-0.978)
LASSO	Categorical	Missing category	All	0.962 (0.947-0.976)
GBM	Categorical	Missing category	All	0.954 (0.938-0.970)
Logistic	Continuous	Imputed	All	0.869 (0.833-0.905)
Logistic	Continuous	Imputed	All with splines*	0.891 (0.864-0.919)
LASSO	Continuous	Imputed	All	0.885 (0.857-0.914)
LASSO	Continuous	Imputed	All with splines*	0.905 (0.88-00.931)
GBM	Continuous	Imputed	All	0.897 (0.871-0.924)
GBM	Continuous	Imputed	All with splines*	0.899 (0.870-0.928)
Logistic	Continuous	Imputed	Stepwise selection	0.894 (0.867-0.921)
Logistic	Continuous	Imputed	Stepwise selection with splines*	0.908 (0.883-0.934)

All = all demographic, symptoms, vital signs/physiological measures, and laboratory test result candidate predictors. *Splines were used to model continuous variables where non-liner associations with the outcome were observed. LASSO = least absolute shrinkage and selection operator; GBM = gradient boosting machine.

For peer review only

Supplementary Table 5. Logistic regression model coefficients for intensive therapy unit admission within 28 days of hospital admission in the derivation dataset (University Hospitals Birmingham)

Predictor	Coefficient
Intercept	-1.394
Age category (years), n (%)	
<30	Ref
30-39	0.221
40-49	0.637
50-59	1.046
60-69	0.296
70-79	-0.282
80-89	-2.240
≥90	-15.912
Sex (male)	0.791
Fever	0.429
New onset diarrhoea or vomiting	-0.808
Temperature (Celsius), n (%)	
<37.8	Ref
≥37.8	0.605
Heart rate category (beats per minute), n (%)	
<80	Ref
80-99	-0.572
≥100	-0.818
Respirations (per minute), n (%)	
<20	Ref
≥20	0.541
Portable oxygen concentrator fraction of inspired oxygen (%), n (%)	
≤0.28	Ref
0.28-0.49	1.206
≥0.5	1.766
pH, n (%)	
<7.30	Ref
7.30-7.34	-1.912
7.35-7.44	-1.740
≥7.45	-1.243
White blood cell count ($10^{!}$/l), n (%)	
<3.9	Ref
3.9-10.8	0.757
≥10.9	2.203
C-reactive protein (mg/l), n (%)	
<10	Ref
10-99	0.241
≥100	0.634
Albumin (g/l), n (%)	
<25	Ref

For peer review only

25-34	-0.835
≥35	-0.913
Monocytes ($10^{!}$/l), n (%)	
<0.2	Ref
0.2-0.8	0.068
>0.8	-0.956
eGFR (ml/min), n (%)	
<30 (Stage 4 or above)	Ref
30-59 (Stage 3)	1.079
60-89 (Stage 2)	0.524
>90 (Normal or high)	0.723
Frailty score, n (%)	
1-3	Ref
4-6	-0.519
7-9	-1.929
Glasgow Coma Scale score, n (%)	
<15	Ref
15	-1.182

(Coefficients derived after a single imputation of missing data.)

For peer review only

Supplementary Table 6. Logistic regression model coefficients for the external validation model for mortality within 28 days of hospital admission (developed in University Hospitals Birmingham data and validated in CovidCollab)

Predictor	Coefficient
Intercept	-16.189
Age category (years), n (%)	
<30	Ref
30-39	13.569
40-49	12.853
50-59	14.234
60-69	14.451
70-79	15.180
80-89	15.461
≥90	15.803
Temperature (Celsius), n (%)	
<37.8	Ref
≥37.8	0.402
Systolic blood pressure (mm Hg), n (%)	
<140	Ref
≥140	-0.361
Respirations (per minute), n (%)	
<20	Ref
≥20	0.412
Oxygen saturation (%), n (%)	
<80	Ref
80-88	0.452
89-93	-0.564
≥94	-1.045
Portable oxygen concentrator fraction of inspired oxygen (%), n (%)	
≤0.28	Ref
0.28-0.49	0.562
≥0.5	0.632
pH, n (%)	
<7.30	Ref
7.30-7.34	-0.945
7.35-7.44	-0.535
≥7.45	-0.352
C-reactive protein (mg/l), n (%)	
<10	Ref
10-99	0.607
≥100	1.110
Urea (mmol/l), n (%)	
<7.8	Ref
≥7.8	0.489
Frailty score, n (%)	

For peer review only

1-3	Ref
4-6	0.166
7-9	0.659

(Coefficients derived after a single imputation of missing data.)

For peer review only

Supplementary Table 7. Logistic regression model coefficients for the external validation model for intensive therapy unit admission within 28 days of hospital admission (developed in University Hospitals Birmingham data and validated in CovidCollab)

Predictor	Coefficient
Intercept	-2.155
Age category (years), n (%)	
<30	Ref
30-39	0.309
40-49	0.651
50-59	0.969
60-69	0.319
70-79	-0.110
80-89	-1.887
≥90	-15.724
Sex (male)	0.683
Fever	0.448
Respirations (per minute), n (%)	
<20	Ref
≥20	0.433
Portable oxygen concentrator fraction of inspired oxygen (%), n (%)	
≤0.28	Ref
0.28-0.49	1.180
≥0.5	1.816
pH, n (%)	
<7.30	Ref
7.30-7.34	-1.776
7.35-7.44	-1.925
≥7.45	-1.388
C-reactive protein (mg/l), n (%)	
<10	Ref
10-99	-0.018
≥100	0.612
Neutrophil:lymphocyte ratio, n (%)	
<2.21	Ref
2.21-4.82	0.747
>4.82	1.143
eGFR (ml/min), n (%)	
<30 (Stage 4 or above)	Ref
30-59 (Stage 3)	0.936
60-89 (Stage 2)	0.363
>90 (Normal or high)	0.606
Frailty score, n (%)	
1-3	Ref
4-6	-0.424
7-9	-1.852

For peer review only

Glasgow Coma Scale score, n (%)	
<15	Ref
15	-1.139

(Coefficients derived after a single imputation of missing data.)

For peer review only

Supplementary table 8. Sensitivity analysis – complete case analysis and exploration of different numbers of missing candidate predictors: Area under the receiver operating characteristic curve (AUROC) in the University Hospitals Birmingham dataset for predicting mortality and intensive therapy unit admission within 28 days of hospital admission

Number of missing predictor variables per patient	n	Outcome	
		Death	ITU
0	224	0.690 (0.588-0.791)	0.887 (0.825-0.949)
0-1	471	0.767 (0.715-0.819)	0.885 (0.843-0.927)
0-2	564	0.761 (0.711-0.811)	0.880 (0.848-0.912)
0-5	643	0.753 (0.709-0.797)	0.890 (0.860-0.921)
0-10	911	0.770 (0.735-0.805)	0.883 (0.855-0.912)

Supplementary table 9. Sensitivity analysis – stratification by gender: Area under the receiver operating characteristic curve (AUROC) in the University Hospitals Birmingham dataset for predicting mortality and intensive therapy unit admission within 28 days of hospital admission

Model		Outcome	
Derivation/Train	Test	Death	ITU
Fitted on all patients	Male	0.766 (0.700-0.831)	0.893 (0.823-0.963)
Fitted on all patients	Female	0.788 (0.729-0.847)	0.893 (0.851-0.935)
Fitted on males	Male	0.738 (0.678-0.798)	0.837 (0.770-0.905)
Fitted on females	Female	0.779 (0.726-0.832)	0.884 (0.842-0.926)

For peer review only

Supplementary figures:

Supplementary Figure 1. Exploration of non-linear relationships between continuous predictors and 28-day mortality using general additive models (GAM); splines (continuous smoothed predictors) were included in the models where a non-linear relationship was observed.

Supplementary Figure 2. Exploration of non-linear relationships between continuous predictors and intensive therapy unit admission using general additive models (GAM); splines (continuous smoothed predictors) were included in the models where a non-linear relationship was observed.

Supplementary Figure 3. Forest plot of logistic regression coefficients for days 0-7 from admission for mortality outcome.

For peer review only

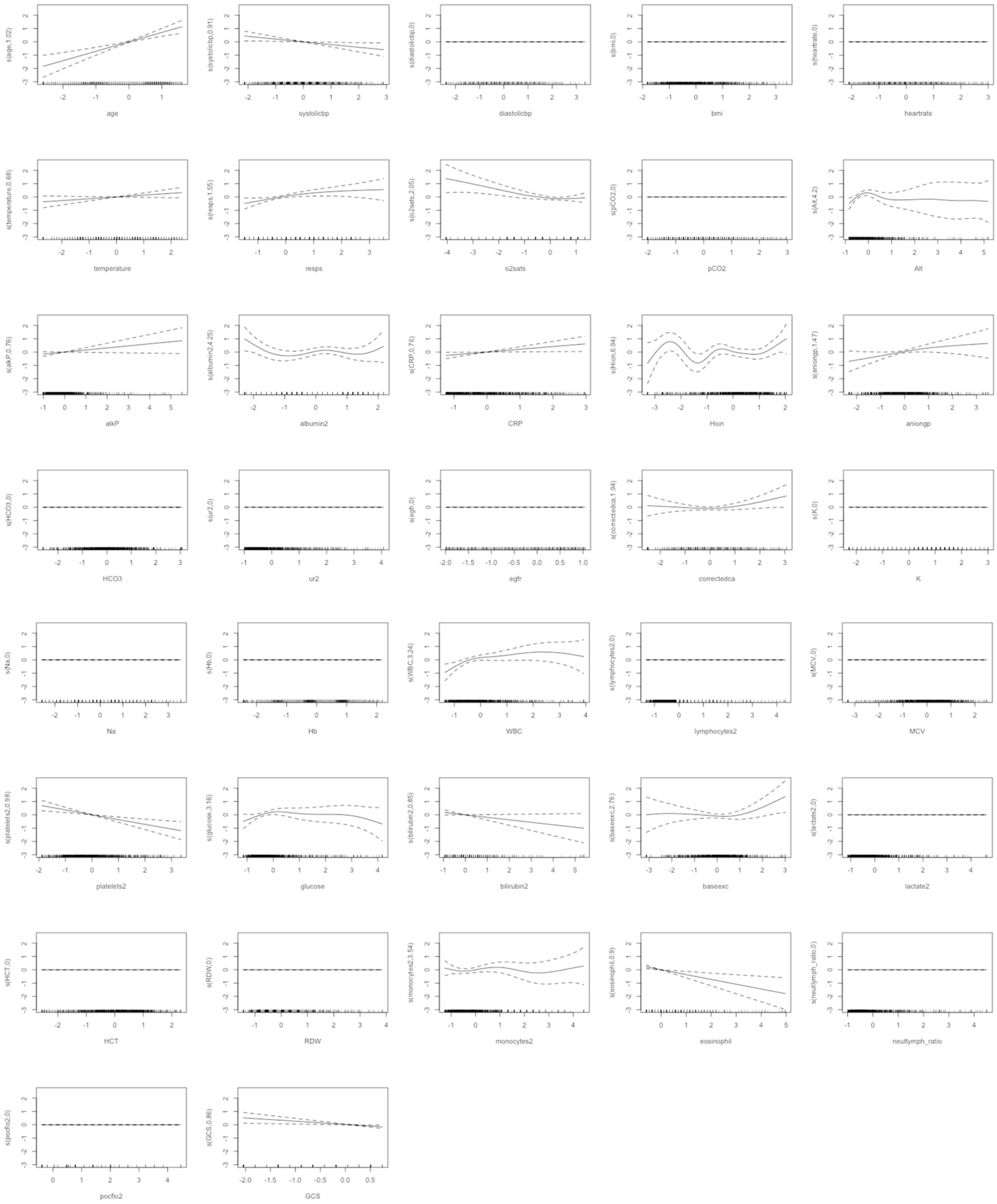
age
systolicbp
diastolicbp
bmi
heartrate
temperature
resps
o2sats
pCO2
Alt
alkP
albumin2
CRP
Hion
aniongp
HCO3
ur2
egfr
correctedca
K
Na
Hb
WBC
lymphocytes2
MCV
platelets2
glucose
bilirubin2
baseexc
lactate2
HCT
RDW
monocytes2
eosinophil
neutlymph_ratio
pocfio2
GCS

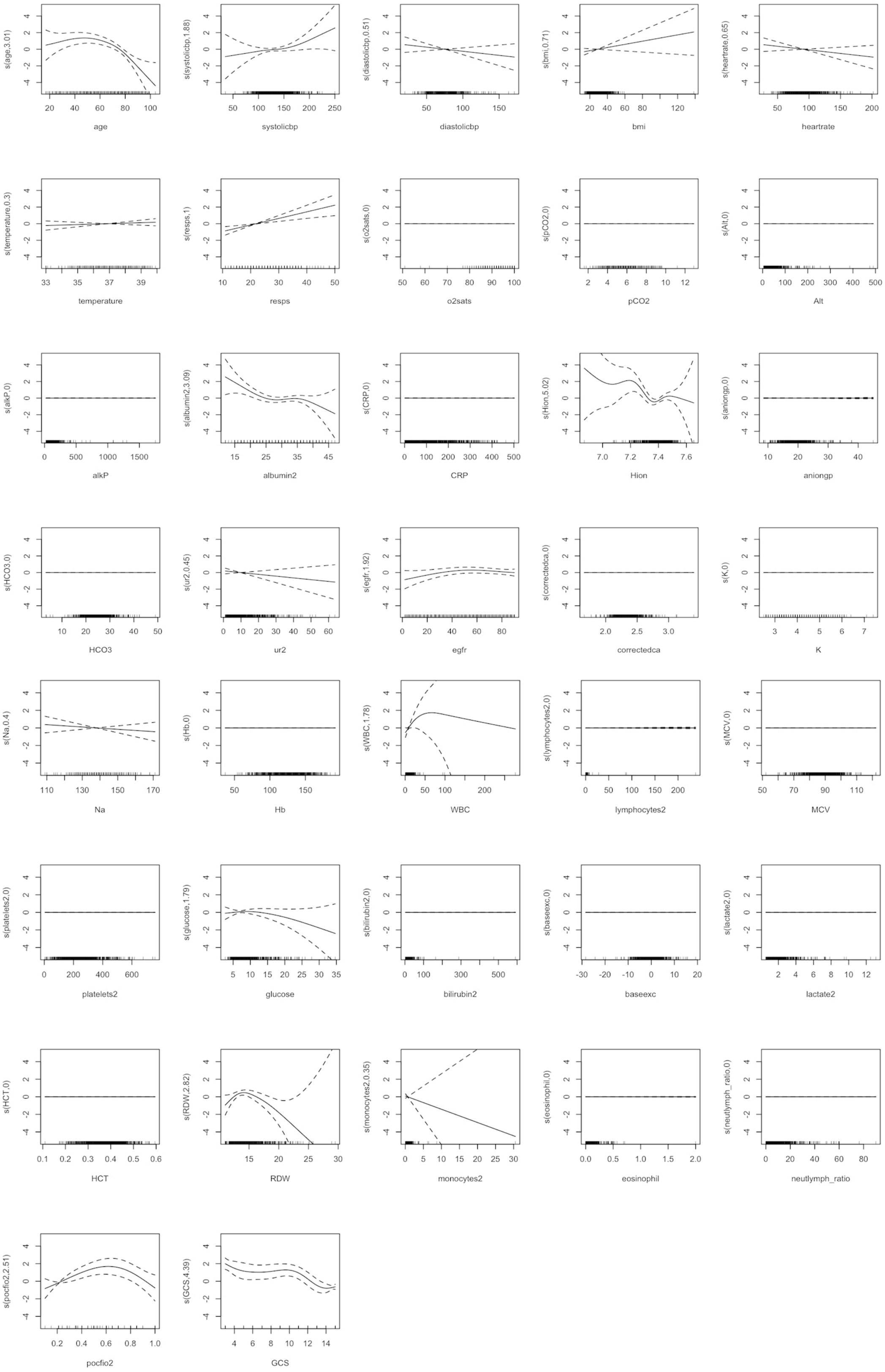
s(age,3.01)
age
s(systolicbp,1.88)
systolicbp
s(diastolicbp,0.51)
diastolicbp
s(bmi,0.71)
bmi
s(heartrate,0.65)
heartrate
s(temperature,0.3)
temperature
s(resps,1)
resps
s(o2sats,0)
o2sats
s(pCO2,0)
pCO2
s(Alt,0)
Alt
s(alkP,0)
alkP
s(albumin2,3.09)
albumin2
s(CRP,0)
CRP
s(Hion,5.02)
Hion
s(aniongp,0)
aniongp
s(HCO3,0)
HCO3
s(ur2,0.45)
ur2
s(egfr,1.92)
egfr
s(correctedca,0)
correctedca
s(K,0)
K
s(Na,0.4)
Na
s(Hb,0)
Hb
s(WBC,1.78)
WBC
s(lymphocytes2,0)
lymphocytes2
s(MCV,0)
MCV
s(platelets2,0)
platelets2
s(glucose,1.79)
glucose
s(bilirubin2,0)
bilirubin2
s(baseexc,0)
baseexc
s(lactate2,0)
lactate2
s(HCT,0)
HCT
s(RDW,2.82)
RDW
s(monocytes2,0.35)
monocytes2
s(eosinophil,0)
eosinophil
s(neutlymph_ratio,0)
neutlymph_ratio
s(pocfio2,2.51)
pocfio2
s(GCS,4.39)
GCS

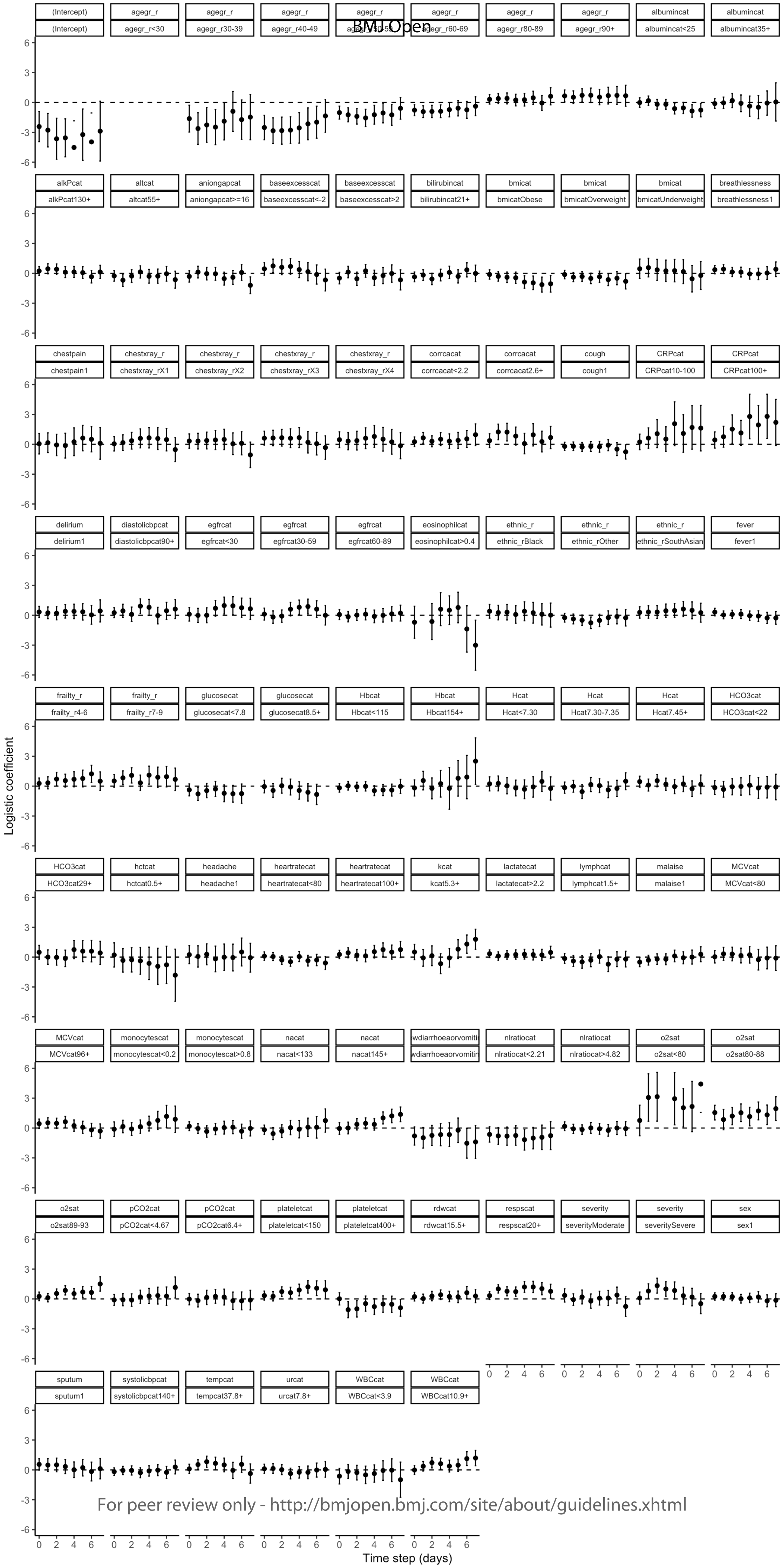
(Intercept) (Intercept)
agegr_r agegr_r<30
agegr_r agegr_r30-39
agegr_r agegr_r40-49
agegr_r agegr_r50-59
agegr_r agegr_r60-69
agegr_r agegr_r80-89
agegr_r agegr_r90+
albumincat albumincat<25
albumincat albumincat35+
alkPcat alkPcat130+
altcat altcat55+
aniongapcat aniongapcat>=16
baseexcesscat baseexcesscat<-2
baseexcesscat baseexcesscat>2
bilirubincat bilirubincat21+
bmicat bmicatObese
bmicat bmicatOverweight
bmicat bmicatUnderweight
breathlessness breathlessness1
chestpain chestpain1
chestxray_r chestxray_rX1
chestxray_r chestxray_rX2
chestxray_r chestxray_rX3
chestxray_r chestxray_rX4
corrcacat corrcacat<2.2
corrcacat corrcacat2.6+
cough cough1
CRPcat CRPcat10-100
CRPcat CRPcat100+
delirium delirium1
diastolicbpcat diastolicbpcat90+
egfrcat egfrcat<30
egfrcat egfrcat30-59
egfrcat egfrcat60-89
eosinophilcat eosinophilcat>0.4
ethnic_r ethnic_rBlack
ethnic_r ethnic_rOther
ethnic_r ethnic_rSouthAsian
fever fever1
frailty_r frailty_r4-6
frailty_r frailty_r7-9
glucosecat glucosecat<7.8
glucosecat glucosecat8.5+
Hbcat Hbcat<115
Hbcat Hbcat154+
Hcat Hcat<7.30
Hcat Hcat7.30-7.35
Hcat Hcat7.45+
HCO3cat HCO3cat<22
HCO3cat HCO3cat29+
hctcat hctcat0.5+
headache headache1
heartratecat heartratecat<80
heartratecat heartratecat100+
kcat kcat5.3+
lactatecat lactatecat>2.2
lymphcat lymphcat1.5+
malaise malaise1
MCVcat MCVcat<80
MCVcat MCVcat96+
monocytescat monocytescat<0.2
monocytescat monocytescat>0.8
nacat nacat<133
nacat nacat145+
wdiarrhoeaorvomiti wdiarrhoeaorvomiti
nlratiocat nlratiocat<2.21
nlratiocat nlratiocat>4.82
o2sat o2sat<80
o2sat o2sat80-88
o2sat o2sat89-93
pCO2cat pCO2cat<4.67
pCO2cat pCO2cat6.4+
plateletcat plateletcat<150
plateletcat plateletcat400+
rdwcat rdwcat15.5+
respscat respscat20+
severity severityModerate
severity severitySevere
sex sex1
sputum sputum1
systolicbpcat systolicbpcat140+
tempcat tempcat37.8+
urcat urcat7.8+
WBCcat WBCcat<3.9
WBCcat WBCcat10.9+
Logistic coefficient
Time step (days)
6
3
0
-3
-6
0 2 4 6

TRIPOD Checklist: Prediction Model Development and Validation

Section/Topic	Item		Checklist Item	Page
**Title and abstract**				
Title	1	D;V	Identify the study as developing and/or validating a multivariable prediction model, the target population, and the outcome to be predicted.	1
Abstract	2	D;V	Provide a summary of objectives, study design, setting, participants, sample size, predictors, outcome, statistical analysis, results, and conclusions.	3
**Introduction**				
Background and objectives	3a	D;V	Explain the medical context (including whether diagnostic or prognostic) and rationale for developing or validating the multivariable prediction model, including references to existing models.	4
	3b	D;V	Specify the objectives, including whether the study describes the development or validation of the model or both.	5
**Methods**				
Source of data	4a	D;V	Describe the study design or source of data (e.g., randomized trial, cohort, or registry data), separately for the development and validation data sets, if applicable.	5
	4b	D;V	Specify the key study dates, including start of accrual; end of accrual; and, if applicable, end of follow-up.	5-6
Participants	5a	D;V	Specify key elements of the study setting (e.g., primary care, secondary care, general population) including number and location of centres.	5
	5b	D;V	Describe eligibility criteria for participants.	5
	5c	D;V	Give details of treatments received, if relevant.	n/a
Outcome	6a	D;V	Clearly define the outcome that is predicted by the prediction model, including how and when assessed.	5
	6b	D;V	Report any actions to blind assessment of the outcome to be predicted.	n/a
Predictors	7a	D;V	Clearly define all predictors used in developing or validating the multivariable prediction model, including how and when they were measured.	6, 9
	7b	D;V	Report any actions to blind assessment of predictors for the outcome and other predictors.	n/a
Sample size	8	D;V	Explain how the study size was arrived at.	5
Missing data	9	D;V	Describe how missing data were handled (e.g., complete-case analysis, single imputation, multiple imputation) with details of any imputation method.	7
Statistical analysis methods	10a	D	Describe how predictors were handled in the analyses.	6-7
	10b	D	Specify type of model, all model-building procedures (including any predictor selection), and method for internal validation.	6-7
	10c	V	For validation, describe how the predictions were calculated.	7-8
	10d	D;V	Specify all measures used to assess model performance and, if relevant, to compare multiple models.	7
	10e	V	Describe any model updating (e.g., recalibration) arising from the validation, if done.	7-8
Risk groups	11	D;V	Provide details on how risk groups were created, if done.	8
Development vs. validation	12	V	For validation, identify any differences from the development data in setting, eligibility criteria, outcome, and predictors.	n/a
**Results**				
Participants	13a	D;V	Describe the flow of participants through the study, including the number of participants with and without the outcome and, if applicable, a summary of the follow-up time. A diagram may be helpful.	9, 11
	13b	D;V	Describe the characteristics of the participants (basic demographics, clinical features, available predictors), including the number of participants with missing data for predictors and outcome.	9, Table 1
	13c	V	For validation, show a comparison with the development data of the distribution of important variables (demographics, predictors and outcome).	Table 1
Model development	14a	D	Specify the number of participants and outcome events in each analysis.	9, 11, Table 1
	14b	D	If done, report the unadjusted association between each candidate predictor and outcome.	n/a
Model specification	15a	D	Present the full prediction model to allow predictions for individuals (i.e., all regression coefficients, and model intercept or baseline survival at a given time point).	Suppl. Tables 3, 5-7
	15b	D	Explain how to the use the prediction model.	13
Model performance	16	D;V	Report performance measures (with CIs) for the prediction model.	10-12
Model-updating	17	V	If done, report the results from any model updating (i.e., model specification, model performance).	11-12
**Discussion**				
Limitations	18	D;V	Discuss any limitations of the study (such as nonrepresentative sample, few events per predictor, missing data).	14
Interpretation	19a	V	For validation, discuss the results with reference to performance in the development data, and any other validation data.	13-14
	19b	D;V	Give an overall interpretation of the results, considering objectives, limitations, results from similar studies, and other relevant evidence.	15
Implications	20	D;V	Discuss the potential clinical use of the model and implications for future research.	15
**Other information**				
Supplementary information	21	D;V	Provide information about the availability of supplementary resources, such as study protocol, Web calculator, and data sets.	Suppl. Tables
Funding	22	D;V	Give the source of funding and the role of the funders for the present study.	16

For peer review only

TRIPOD Checklist: Prediction Model Development and Validation

*Items relevant only to the development of a prediction model are denoted by D, items relating solely to a validation of a prediction model are denoted by V, and items relating to both are denoted D;V. We recommend using the TRIPOD Checklist in conjunction with the TRIPOD Explanation and Elaboration document.

For peer review only

BMJ Open

Development and external validation of prognostic models for COVID-19 to support risk stratification in secondary care

Journal:	*BMJ Open*
Manuscript ID	bmjopen-2021-049506.R1
Article Type:	Original research
Date Submitted by the Author:	22-Jun-2021
Complete List of Authors:	Adderley, Nicola; University of Birmingham, Institute of Applied Health Research<br>Taverner, Thomas ; University of Birmingham, Institute of Applied Health Research<br>Price, Malcolm; University of Birmingham, Institute of Applied Health Research; University Hospitals Birmingham NHS Foundation Trust, NIHR Birmingham Biomedical Research Centre<br>Sainsbury, Christopher; University of Birmingham, Institute of Applied Health Research; Gartnavel General Hospital, Department of Diabetes<br>Greenwood, David; University of Birmingham, Institute of Applied Health Research<br>Chandan, Joht; University of Birmingham, Institute of Applied Health Research<br>Takwoingi, Yemisi; University of Birmingham, Institute of Applied Health Research; University Hospitals Birmingham NHS Foundation Trust, NIHR Birmingham Biomedical Research Centre<br>Haniffa, Rashan; University of Oxford, Mahidol Oxford Tropical Medicine Research Unit; University College London Hospitals NHS Foundation Trust<br>Hosier, Isaac; University of Birmingham, Institute of Applied Health Research<br>Welch, Carly; University of Birmingham, Institute of Inflammation and Ageing; University Hospitals Birmingham NHS Foundation Trust<br>Parekh, Dhruv; University of Birmingham, Institute of Inflammation and Ageing; University Hospitals Birmingham NHS Foundation Trust<br>Gallier, Suzy; University Hospitals Birmingham NHS Foundation Trust<br>Gokhale, Krishna; University of Birmingham, Institute of Applied Health Research<br>Denniston, Alastair; University of Birmingham, Institute of Inflammation and Ageing; Moorfields Eye Hospital NHS Foundation Trust, National Institute for Health Research Biomedical Research Centre<br>Sapey, Elizabeth; University of Birmingham, Institute of Inflammation and Ageing; University Hospitals Birmingham NHS Foundation Trust<br>Nirantharakumar, Krishnarajah; University of Birmingham, Institute of Applied Health Research; Health Data Research UK
**Primary Subject Heading**:	Infectious diseases
Secondary Subject Heading:	Health services research, Intensive care, Health informatics, Evidence based practice

Keywords:	PUBLIC HEALTH, Public health < INFECTIOUS DISEASES, COVID-19

SCHOLARONE™
Manuscripts

I, the Submitting Author has the right to grant and does grant on behalf of all authors of the Work (as defined in the below author licence), an exclusive licence and/or a non-exclusive licence for contributions from authors who are: i) UK Crown employees; ii) where BMJ has agreed a CC-BY licence shall apply, and/or iii) in accordance with the terms applicable for US Federal Government officers or employees acting as part of their official duties; on a worldwide, perpetual, irrevocable, royalty-free basis to BMJ Publishing Group Ltd ("BMJ") its licensees and where the relevant Journal is co-owned by BMJ to the co-owners of the Journal, to publish the Work in this journal and any other BMJ products and to exploit all rights, as set out in our licence.

The Submitting Author accepts and understands that any supply made under these terms is made by BMJ to the Submitting Author unless you are acting as an employee on behalf of your employer or a postgraduate student of an affiliated institution which is paying any applicable article publishing charge ("APC") for Open Access articles. Where the Submitting Author wishes to make the Work available on an Open Access basis (and intends to pay the relevant APC), the terms of reuse of such Open Access shall be governed by a Creative Commons licence – details of these licences and which Creative Commons licence will apply to this Work are set out in our licence referred to above.

Other than as permitted in any relevant BMJ Author's Self Archiving Policies, I confirm this Work has not been accepted for publication elsewhere, is not being considered for publication elsewhere and does not duplicate material already published. I confirm all authors consent to publication of this Work and authorise the granting of this licence.

For peer review only

Development and external validation of prognostic models for COVID-19 to support risk stratification in secondary care

Nicola J Adderley PhD,[1] Thomas Taverner PhD,[1] Malcolm Price PhD,[1,2] Christopher Sainsbury MD,[1,3] David Greenwood MSc,[1] Joht Singh Chandan PhD,[1] Yemisi Takwoingi PhD,[1,2] Rashan Haniffa PhD,[4,5,6] Isaac Hosier BMedSci,[1] Carly Welch MRCP,[7,8,9] Dhruv Parekh PhD,[7,8] Suzy Gallier BSc,[8] Krishna Gokhale MSc,[1] Alastair K Denniston PhD FRCOphth,[7,10,11] Elizabeth Sapey FRCP,[7,8,11] Krishnarajah Nirantharakumar MD[1,11]

Affiliations:
1. Institute of Applied Health Research, University of Birmingham, Edgbaston, Birmingham, B15 2TT, UK
2. NIHR Birmingham Biomedical Research Centre, University Hospitals Birmingham NHS Foundation Trust and University of Birmingham, UK
3. Department of Diabetes, Gartnavel General Hospital, NHS Greater Glasgow and Clyde, Glasgow, UK
4. National Intensive Care Surveillance-MORU, Colombo, Sri Lanka
5. Mahidol Oxford Tropical Medicine Research Unit, University of Oxford
6. University College London Hospitals, London, United Kingdom
7. Institute of Inflammation and Ageing, University of Birmingham, Birmingham, UK
8. University Hospitals Birmingham NHS Foundation Trust, Birmingham, UK
9. Medical Research Council and Versus Arthritis Centre for Musculoskeletal Ageing Research, University of Birmingham and University of Nottingham, Birmingham and Nottingham, UK
10. University College London/Moorfields Eye Hospital National Institute for Health Research Biomedical Research Centre
11. Health Data Research UK

Corresponding author:
Nicola J Adderley, n.j.adderley@bham.ac.uk
ORCID: 0000-0003-0543-3254

Abstract: 289
Word count: 5269
Tables: 4 (+9 supplementary)
Figures: 1 (+4 supplementary)

For peer review only

For peer review only - http://bmjopen.bmj.com/site/about/guidelines.xhtml

Abstract

Objectives

Existing UK prognostic models for patients admitted to hospital with COVID-19 are limited by reliance on comorbidities, which are under-recorded in secondary care, and lack of imaging data among the candidate predictors. Our aims were to develop and externally validate novel prognostic models for adverse outcomes (death, intensive therapy unit (ITU) admission) in UK secondary care; and externally validate the existing 4C score.

Design

Candidate predictors included demographic variables, symptoms, physiological measures, imaging, laboratory tests. Final models used logistic regression with stepwise selection.

Setting

Model development was performed in data from University Hospitals Birmingham (UHB). External validation was performed in the CovidCollab dataset.

Participants

Patients with COVID-19 admitted to UHB January-August 2020 were included.

Main outcome measures

Death and ITU admission within 28 days of admission.

Results

1040 patients with COVID-19 were included in the derivation cohort; 288 (28%) died and 183 (18%) were admitted to ITU within 28 days of admission. Area under the receiver operating curve (AUROC) for mortality was 0.791 (95%CI 0.761-0.822) in UHB and 0.767 (95%CI 0.754-0.780) in CovidCollab; AUROC for ITU admission was 0.906 (95%CI 0.883-0.929) in UHB and 0.811 (95%CI 0.795-0.828) in CovidCollab. Models showed good calibration. Addition of comorbidities to candidate predictors did not improve model performance. AUROC for the ISARIC 4C score in the UHB dataset was 0.753 (95%CI 0.720-0.785).

Conclusions

The novel prognostic models showed good discrimination and calibration in derivation and external validation datasets, and performed at least as well as the existing 4C score using only routinely collected patient information. The models can be integrated into electronic medical records systems to calculate each individual patient's probability of death or ITU admission at the time of hospital admission. Implementation of the models and clinical utility should be evaluated.

For peer review only - http://bmjopen.bmj.com/site/about/guidelines.xhtml

Article Summary

Strengths and limitations of this study

- The UHB development dataset represents one of the largest and most ethnically diverse patient cohorts within the UK.
- As part of the UHB COVID-19 response, all admitted patients underwent a wide range of investigations to support international research efforts examining prognostic markers allowing assessment of a wide range of possible predictors (demographic variables, symptoms, physiological measures, imaging, laboratory test results) with low levels of missing data.
- A limitation of the study was that the overall sample size was relatively small compared to that of the ISARIC study and was limited to one UK geographical location.
- In the external validation cohort we were unable to examine all of the predictors included in the original full UHB model due to only a reduced set of candidate predictors being available in CovidCollab.
- It was not possible to carry out stratified analysis by ethnicity as the UHB dataset contained too few patients in many of the strata, and no ethnicity data was available in the CovidCollab dataset.

For peer review only - http://bmjopen.bmj.com/site/about/guidelines.xhtml

Background

The coronavirus disease 2019 (COVID-19) pandemic has placed exceptional strain on health care systems globally. Health systems, and especially critical care services, can be overwhelmed given the number of patients, and the duration and severity of their illness. A proportion of patients with COVID-19 can deteriorate rapidly. Clinicians need to differentiate between those with COVID-19 who are at high risk of the most severe symptoms (requiring intensive care treatment/ventilation) or death, and those who can be considered low risk and potentially managed in the community. Early identification of patients at highest risk of severe outcomes may provide opportunity to prioritise, intervene and improve outcomes.

Objective prognostic tools for patients with COVID-19, based on patients' initial characteristics, symptoms, biomarkers and imaging at the time of hospital admission, which can be used at or just after admission, and which can accurately discriminate between patients who will progress to more severe symptoms or death and those who will not, can be used by clinicians to triage and manage patients. This could potentially reduce time to appropriate interventions and improve patient outcomes.

A rapid systematic review has identified a number of prediction models developed for COVID-19, including prognostic models.[1] However, while these existing studies provided useful information on candidate predictors for further exploration, the review found substantial limitations: many models were developed exclusively in a Chinese population; many were at high risk of bias, particularly in terms of inclusion of non-representative control participants, inappropriate exclusion criteria, and small sample sizes leading to high risk of overfitting; and external validation was limited.[1] Other studies have evaluated existing early warning scores such as the National Early Warning Score (NEWS2), but with conflicting findings regarding their utility in predicting COVID-19 outcomes.[2],[3]

More recent models have since been developed,[4,5] some of which overcome a number of these limitations, including the International Severe Acute Respiratory and emerging Infection Consortium (ISARIC) model and corresponding (simplified) 4C score, which was developed in a UK secondary care population representing 260 hospitals in England, Scotland and Wales (the ISARIC dataset).[5] Whilst the 4C score showed reasonable discrimination for mortality, there are some limitations, including a reliance on clinicians counting specific comorbidities, which may not be recorded at admission and which are known to be under-recorded in secondary care,[6] and an absence of imaging data among the candidate predictors.

Aims and rationale

To date there have been few prognostic models for patients admitted to hospital with COVID-19 developed in a UK dataset. Furthermore, evaluation of the extent to which the inclusion of comorbidities, imaging and additional biomarkers improves model performance is required. It also remains to be determined whether updating the clinical parameters with evolving biomarkers improves prediction of the clinical course of patients as the disease evolves.

For peer review only - http://bmjopen.bmj.com/site/about/guidelines.xhtml

The overarching aim of this study was to develop prognostic models for patients admitted to hospital with COVID-19 using routinely collected data at the point of admission, which can be used in a secondary care setting to support clinical decision-making. Specific objectives were: (i) to develop novel prognostic models for calculating predicted probability of adverse outcomes (death, intensive therapy unit (ITU) admission) at an individual patient level in a UK secondary care setting; (ii) to externally validate these models in an international dataset (including data from UK hospitals); (iii) to externally validate the existing UK ISARIC 4C score;[5] and (iv) to compare performance of the newly developed models with the UK ISARIC 4C score. In addition, we developed daily models using time series data from the first eight days from admission to explore changes in predictors over time.

Methods

Data source

Data from University Hospitals Birmingham NHS Foundation Trust (UHB) was sourced via the PIONEER Health Data Research Hub for acute care, and used for model development and for external validation of the ISARIC 4C score. Data from patients with COVID-19 admitted to Queen Elizabeth Hospital, Birmingham (part of UHB), between 1st January 2020 and 16th August 2020 was included. Data included symptoms recorded at admission, comorbidities (from International Classification of Diseases 10th revision (ICD-10) discharge codes), vital signs (e.g. blood pressure, oxygen saturation), laboratory results (biochemistry, haematology, microbiology, pathology), imaging, and outcomes (ITU admission and death).

External validation of the newly developed models was performed in the CovidCollab dataset. CovidCollab is an international project utilising routinely collected health care data to develop a better understanding of how best to treat and care for adults with COVID-19.[7,8] The dataset includes symptoms, comorbidities, vital signs, laboratory results, imaging findings, and outcomes.

Study population

Patients of all ages diagnosed with COVID-19 and hospitalised were included. Diagnosis was defined as a positive test result for SARS-CoV-2 from one or more reverse transcription polymerase chase reaction (RT-PCR) or transcription mediated amplification tests. In the CovidCollab dataset, COVID-19 diagnosis was by either PCR or antibody test. Anonymised data for all patients with COVID-19 admitted to UHB during the study period was included. For CovidCollab, data collection was dependent on the specific processes within individual participating hospitals and the capacity of the data collector.[7]

For peer review only - http://bmjopen.bmj.com/site/about/guidelines.xhtml

Study Design

Retrospective cohort analyses; index date (start of follow-up) was the hospital admission date. Study period was 1st January 2020 to 12th September 2020 (last admission date was 16th August to ensure a minimum of 28 days of follow-up).

Outcomes

Primary outcome was death within 28 days of admission (in-hospital or post-discharge). Secondary outcome was ITU admission within 28 days of admission.

Study follow-up

Participants were followed from index (admission) date until the earliest of outcome date or study end (latest available data, 12th September 2020). Participants were censored 28 days after index date. Participants admitted after 16th August 2020 (less than 28 days prior to study end date) were excluded.

Candidate predictor variables

Candidate predictors were selected a priori following a review of existing literature, discussion with clinical experts (specialists in acute care, critical care and geriatric medicine), and based on availability of variables routinely collected in secondary care/UHB. These included demographic variables, symptoms, comorbidities, physiological measures, imaging findings and laboratory test results. Comorbidities are not reliably and completely collected at admission, with the most complete hospital record of comorbidities usually being the discharge ICD-10 codes; therefore, the development and performance of models with and without comorbidity predictors were compared in order to explore the potential for developing models which would require no additional data collection (other than routinely collected data) at the point of admission.

Model development

Models were trained using UHB data (patients admitted up to and including 16th August 2020). We used a multi-stage model building process that assessed the impact of a range of feature representation and modelling choices to select important candidate predictors. All analyses were performed in R.

Three sets of models were fitted which incorporated continuous variables in three different ways, to explore the impact of treating these variables as continuous or categorical, and also to explore the impact of different methods of handling missing data:

(a) as continuous numeric values, with missing values imputed (“continuous”);
(b) as categorical values derived from the imputed continuous values (“categorical-imputed”);
(c) and, in secondary analysis, as categorical values, using clinically meaningful categories and reference ranges, with missing indicators as a separate category (“categorical”).

For peer review only - http://bmjopen.bmj.com/site/about/guidelines.xhtml

For the three ways of handling numeric features and missing variables above, we fitted outcomes of death within 28 days and ITU admission (within 28 days) to candidate predictors using a range of models, which allowed both linear relationships and complex interactions between variables:

(a) logistic regression with i) all baseline parameters (demographic variables, symptoms, vital signs/physiological measures, and laboratory test results), ii) demographic variables only, and iii) all baseline parameters with the addition of recorded comorbidities (recorded up to the point of discharge);
(b) logistic regression with stepwise AIC minimisation, both forward and backward;[9]
(c) LASSO (l1 penalized) logistic regression using all baseline parameters;
(d) gradient boosted model (GBM) using all baseline parameters with default hyperparameter values of 150 trees, maximum interaction depth 3, minimum of 10 observations in nodes, and shrinkage 0.1.[10]

Further information on handling of continuous variables is presented in Supplementary Appendix 1.

For each of these four variable selection models, in order to reduce overfitting and selection bias, we internally validated using 5-fold cross-validation (80/20 train/test split) to derive the candidate variable list. To avoid sensitivity to imputation, this cross-validation was repeated for each of the 5 multiple imputations.

Due to the relatively small number of outcome events (<300) we did not attempt to systematically look for interactions between multiple variables.

Model performance

Model performance (discrimination) was assessed by calculating the area under the receiver operating characteristics curve (AUROC, or C-statistic).[11] Calibration was assessed by plotting observed probability of the outcome against predicted probability, and by calculating the calibration slope and intercept. We also calculated sensitivity, specificity, positive predicted value (PPV) and negative predictive value (NPV) for the final models. For each feature set and each model, the final results for cross-validated (optimism-adjusted) AUROC and all other metrics (including calibration plots) were combined from all the multiple imputations of the dataset using Rubin's rules for the mean and confidence interval (derived from the standard deviation).[12]

Missing data

Information on candidate predictors was collected at the point of admission; however, where information on physiological or laboratory measures was not available on the day of admission, measures recorded up to 72 hours after admission were used. Candidate predictors for which >40% of patients had missing data were excluded from the analysis. Further missing continuous variables (vital signs, laboratory tests) and symptoms were imputed using multiple imputation using chained equations (using the R “mice” multiple imputation package). We performed 5 imputations and a maximum of 50 iterations.[13] Continuous variables were imputed with predictive mean matching, and categorical variables with logistic regression (logreg) or polytomous regression (polyreg). Input variables

For peer review only - http://bmjopen.bmj.com/site/about/guidelines.xhtml

for the multiple imputation included all available candidate predictor variables in the dataset; outcomes were not included in the imputation variables.

We also explored use of a missing category for missing test results. Absence of a record of a comorbidity was taken to indicate absence of the condition.

External validation

To investigate the transferability of models, we performed external validation of logistic regression models derived from the UHB dataset in the CovidCollab dataset, for predicting outcomes of 28-day mortality and ITU admission.

Not all candidate predictors were common to both datasets; therefore, new logistic regression models for death within 28 days and for ITU admission were re-fitted on the UHB data using only those variables also present in the CovidCollab data. We then performed an external validation of these UHB models in the CovidCollab dataset, and ascertained the AUROC in both the UHB and CovidCollab datasets. Based on model performance observed in the initial model derivation and in the interest of clinical utility, we used only categorical rather than continuous numeric variables, with imputed missing values (imputed prior to categorisation). To verify that predictors behaved similarly, we compared logistic coefficients from UHB to the same models fitted on the CovidCollab dataset. To account for sensitivity to missing values, we performed training and testing five times on 5-fold multiply imputed datasets for both UHB and CovidCollab.

External validation of ISARIC 4C score

A logistic regression using the 4C score was performed in the UHB dataset (following the same modelling methods used in the original ISARIC study). Model performance was assessed by calculating the AUROC and plotting calibration curves.

Sensitivity analyses

Most patient records had some missing variables; we therefore performed a complete case analysis, where we re-fitted the best forward stepwise selection model derived using the full set of UHB variables to complete case data, then data with ≤1, 2, 5, and 10 missing values, imputing missing values in the same way as above, and examined AUROCs and logistic coefficients for stability.

In addition, we performed sensitivity analyses i) within male and female strata by assessing performance of the final models (AUROC) in male and female patients separately; and ii) within age strata by assessing model performance in patients aged ≤60 and >60 years separately.

Time series analysis

The UHB regression models utilised baseline measurement data collected on admission; where not available at admission, we accepted values up to 72 hours after admission. To investigate fine-grained temporal effects of data acquisition, we produced a series of separate logistic regression models using data collected at different time windows from

For peer review only - http://bmjopen.bmj.com/site/about/guidelines.xhtml

within 24 hours of admission up to within 7 days of admission, in 1-day increments, for the mortality outcome. Each dataset included only those patients eligible at the end of the window (not dead or discharged). This created eight different sets of predictors, including baseline variables of age, gender, symptoms, and the time-sensitive variables of the latest physiological and laboratory measurements available.

For missing data, data were carried forward from the first observation (last observation carried forward, LOCF) and 5-fold multiple imputation was performed for missing data after LOCF was done, within each separate time-window dataset. Each model was trained and tested in 5-fold cross validation, within each imputation, and AUROCs averaged using Rubin's rule. We compared the AUROCs for each of the eight models for predicting 28-day mortality from the time of admission and compared the logistic coefficients for the models. For additional insight into possible effects of changing measurements, we produced an additional logistic model for 28-day mortality to time-sensitive data collected within 4 days of admission, augmented with predictors indicating an increase or decrease in the category of each time-sensitive predictor relative to the reference category from 0 to 4 days – for example, whether temperature had crossed from below to above 37.8°C in that period.

Patient and public involvement

We engaged with members of the PIONEER patient and public involvement group during development of the study protocol. We will further engage with this group, as well as other local and national patient and public involvement groups, in order to discuss dissemination of the findings and the best way to communicate these to patients and the public. We also consulted with several secondary care clinicians before and during the study to ensure that the tools developed meet the needs of clinicians. We have engaged with local NHS trusts to ensure that the algorithms developed are implemented/tested in a hospital setting.

Results

Derivation cohort characteristics

A total of 1040 participants with COVID-19 admitted to UHB were included in the derivation cohort. 288 (28%) died within 28 days of admission and 183 (18%) were admitted to ITU. Baseline characteristics are presented in Table 1 (stratified by mortality outcome) and Supplementary Table 1 (stratified by ITU admission). The mean (SD) age of participants was 68.2 (17.7) years; 57% (589) were male; and almost 90% had at least one comorbidity.

Candidate predictors

After exclusion of 7 candidate predictors with >40% missing data (D-dimer, ferritin, high sensitivity troponin, fibrinogen, lactate dehydrogenase, vitamin D, haemoglobin A1c), 63 predictors were considered for inclusion in the models:

Demographic characteristics: age, gender, ethnicity;

Symptoms (binary, presence or absence of symptom at admission): breathlessness, chest pain, cough, fever, headache, malaise, new onset diarrhoea or vomiting, sputum, delirium;

For peer review only - http://bmjopen.bmj.com/site/about/guidelines.xhtml

Physiological measures and vital signs: body mass index (BMI; kg/m^2), systolic blood pressure (mm Hg), diastolic blood pressure (mm Hg), temperature (degrees Celsius), heart rate (beats per minute), respiratory rate (respirations per minute), oxygen saturation (%), partial pressure of CO_2 (kPa), portable oxygen concentrator fraction of inspired oxygen (FiO_2; %);

Imaging: chest X-ray finding (categorised as clear/unchanged, local consolidation, ground glass opacity/bilateral infiltrates, other/no firm diagnosis, none performed/missing);

Scores: Frailty score (Rockwood Clinical Frailty Scale);[14] Glasgow Coma Scale score;[15,16]

Laboratory test results: estimated glomerular filtration rate (eGFR; ml/min), pH (%), base excess (mmol/l), anion gap (mmol/l), white blood cell (WBC) count (10^9/l), platelets (10^9/l), lymphocytes (10^9/l), neutrophil:lymphocyte ratio, mean corpuscular volume (fl), red cell distribution width (%), monocytes (10^9/l), eosinophils (10^9/l), haemoglobin (g/l), glucose (mmol/l), bicarbonate (mmol/l), C-reactive protein (mg/l), albumin (g/l), bilirubin (µmol/l), alanine aminotransferase (U/l), alkaline phosphatase (U/l), urea (mmol/l), potassium (mmol/l), sodium (mmol/l), corrected calcium (mmol/l), lactate (U/l) and haematocrit (l/l);

Comorbidities (binary), presence or absence of record in discharge ICD-10 codes): dementia, cancer, asthma, chronic obstructive pulmonary disease, sleep apnoea, cardiovascular disease, hypertension, diabetes without complications, diabetes with complications, peptic ulcer, liver disease, rheumatic/inflammatory disease, thyroid disorder.

28-day mortality outcome: UHB model and predictive performance

Area under the ROC curve values for each of the logistic, LASSO and GBM models, treating continuous variables in one of three ways (as continuous variables with imputed missing values; as clinically meaningful categorical variables with imputed missing values; and as categorical variables with missing categories), are presented in Supplementary Table 2.

The final model selected was a logistic regression using stepwise selection of variables with categorisation of continuous variables (with imputed missing values). The final 18 categorical predictors included in the model were: age, breathlessness, sputum, systolic blood pressure, temperature, respiratory rate, oxygen saturation, FiO_2, alkaline phosphatase, C-reactive protein, corrected calcium, eosinophils, glucose, pH, urea, WBC count, platelets, and frailty score.

AUROC for the UHB cross-validated model was 0.779 (95 % CI 0.744-0.813) (Table 2). At a 20% predicted probability of mortality, sensitivity was 83% (95% CI 81-85), specificity was 58% (95% CI 55-61), positive predictive value was 43% (95% CI 41-46), and negative predictive was 90% (95% CI 88-91) (Table 3a). Calibration was very good at low to medium predicted probabilities, but was poorer at very high predicted probabilities; a calibration plot is shown in Figure 1A, calibration slope was 0.79 (95% CI 0.64-0.94) (Table 2). Model coefficients (and model equation) are presented in Supplementary Table 3.

Addition of comorbidities to the candidate predictors included in the model did not improve performance of the model (Supplementary Table 2). Since comorbidities are known to be

For peer review only - http://bmjopen.bmj.com/site/about/guidelines.xhtml

under-reported during acute presentations,[6] and they offered no improvement on model performance, models without comorbidities were preferred.

ITU admission: UHB model and predictive performance

Area under the ROC curve values for each of the models performed are presented in Supplementary Table 4.

The final model selected was a logistic regression using stepwise selection of variables with categorisation of continuous variables (with imputed missing values). The final 16 categorical predictors included in the model were: age, gender, fever, new onset diarrhoea or vomiting, heart rate, respiratory rate, FiO_2, temperature, albumin, C-reactive protein, eGFR, pH, monocytes, WBC, frailty score, and Glasgow Coma Scale score.

AUROC was 0.893 (95% CI 0.864-0.922) (Table 2). At a 20% predicted probability of ITU admission, sensitivity was 79% (95% CI 74-84), specificity was 83% (95% CI 81-84), positive predictive value was 49% (95% CI 46-52), and negative predictive was 95% (95% CI 94-96) (Table 3b). Calibration was good; a calibration plot is shown in Figure 1B, calibration slope was 0.91 (95% CI 0.80-1.01) (Table 2). Model coefficients are presented in Supplementary Table 5.

Addition of comorbidities to the predictors included in the model did not improve performance.

Reduced UHB model and external validation in the CovidCollab dataset

A total of 6099 patients admitted with COVID-19 were included in the CovidCollab external validation dataset; 1668 (27%) died and 722 (12%) were admitted to ITU (Table 1 and Supplementary Table 1). Not all variables included in the UHB model derived above were available in the CovidCollab dataset. Therefore, revised and reduced models were developed in UHB data using the subset of candidate predictors common to both the UHB and CovidCollab datasets (reduced UHB dataset, UHB-R), using logistic regression with stepwise selection, and these were then externally validated in the CovidCollab dataset.

The reduced set of 27 candidate predictors included: demographic characteristics: age, gender; symptoms: cough, fever, delirium; physiological measures and vital signs: BMI, systolic blood pressure, diastolic blood pressure, heart rate, temperature, respiratory rate, oxygen saturation, FiO_2, chest X-ray; frailty score; Glasgow Coma Scale score; laboratory test results: eGFR, pH, base excess, lymphocytes, neutrophil:lymphocyte ratio, haemoglobin, bicarbonate, C-reactive protein, alanine aminotransferase, urea, and lactate.

28-day mortality

For the 28-day mortality outcome, following stepwise selection, the final 10 categorical predictors (common to both datasets) included in the reduced logistic regression model were: age, oxygen saturation, FiO_2, respiratory rate, temperature, systolic blood pressure, C-reactive protein, pH, urea, and frailty score.

For peer review only - http://bmjopen.bmj.com/site/about/guidelines.xhtml

The selected predictors were a subset of those in the original UHB model derivation, but gave similar model performance. AUROC in the UHB-R dataset was 0.791 (95% CI 0.761-0.822), and in the CovidCollab external validation dataset was 0.767 (95% CI 0.754-0.780) (Table 2). At a 20% predicted probability of mortality, in the UHB-R dataset sensitivity was 86% (95% CI 85-88), specificity was 54% (95% CI 51-57), PPV was 42% (95% CI 40-43) and NPV was 91% (95% CI 90-92); in the CovidCollab dataset sensitivity was 88% (95% CI 87-89), specificity was 46% (95% CI 45-47), PPV was 38% (95% CI 0.37-0.38), and NPV was 91% (95% CI 91-92) (Table 4a).

Calibration was good for both derivation and external validation datasets; calibration plots are shown in Figures 1C-D, calibration slopes were 0.89 (95% CI 0.81-0.97) and 0.85 (95% CI 0.75-0.94) for the UHB-R and CovidCollab datasets, respectively (Table 2). Model coefficients are presented in Supplementary Table 6.

ITU admission

For the ITU admission outcome, the final 11 categorical predictors (common to both datasets) included in the reduced model were: age, gender, fever, respiratory rate, FiO_2, C-reactive protein, eGFR, pH, neutrophil:lymphocyte ratio, frailty score, and Glasgow Coma Scale score.

AUROC in the UHB-R dataset was 0.906 (95% CI 0.883-0.929), and in the CovidCollab dataset was 0.811 (95% CI 0.795-0.828) (Table 2). At a 20% predicted probability of ITU admission, in the UHB-R dataset sensitivity was 83% (95% CI 81-85), specificity was 83% (95% CI 82-84), PPV was 51% (95% CI 49-52) and NPV was 96% (95% CI 95-96); in the CovidCollab dataset sensitivity was 64% (95% CI 62-67), specificity was 80% (95% CI 79-82), PPV was 30% (95% CI 29-32), and NPV was 94% (95% CI 94-95) (Table 4b).

Calibration was good for both derivation and external validation datasets; calibration plots are shown in Figures 1E-F, calibration slopes were 0.94 (95% CI 0.84-1.04) and 0.95 (95% CI 0.82-1.08) for the UHB-R and CovidCollab datasets, respectively (Table 2). Model coefficients are presented in Supplementary Table 7.

External validation of the ISARIC 4C score in the UHB dataset

The AUROC for the recently published ISARIC 4C score in the UHB dataset was 0.753 (95% CI 0.720-0.785). Calibration slope was 0.99 (95% CI 0.85-1.12) (Table 2 and Supplementary Figure 1).

It was not possible to externally validate the ISARIC 4C score in the CovidCollab dataset, as information on many of the comorbidities required to calculate the ISARIC comorbidity score was not available in the dataset.

Sensitivity analyses

Analyses exploring different ways of handling missing data are reported in Supplementary Appendix 2 and Supplementary Figures 2-3.

For peer review only - http://bmjopen.bmj.com/site/about/guidelines.xhtml

Complete case analysis

Few patients in the dataset had complete data (n = 224/1040, 22%); model performance in this patient subset was slightly poorer for mortality outcome: AUROC 0.696 (95% CI 0.597-0.795) for mortality and 0.892 (95% CI 0.844-0.940) for ITU admission. Including patients with missing variables, with missing values imputed, improved model performance; allowing even a single missing/imputed variable improved AUROC for mortality to 0.760 (95% CI 0.708-0.812) (Supplementary Table 8).

Stratification by gender and age

When patients were stratified by gender, the reduced models predicting mortality and ITU still performed well: AUROCs for mortality were 0.775 (95% CI 0.726-0.823) for males and 0.755 (95% CI 0.706-0.804) for females; and for ITU 0.897 (95% CI 0.856-0.937) for males and 0.873 (95% CI 0.833-0.913) for females. (Supplementary Table 9).

When patients were stratified by age, the models performed slightly better in patients aged >60 years (AUROC 0.778 (95% CI 0.722-0.834) and 0.897 (95% CI 0.864-0.930) for mortality and ITU admission, respectively) compared to those aged ≤60 years (AUROC 0.730 (95% CI 0.638-0.823) and 0.845 (95% CI 0.761-0.930) for mortality and ITU admission, respectively).

Time series analysis

Supplementary Figure 4 shows variation in logistic regression coefficients for the candidate predictors from day of admission and up to 7 days later. The majority of coefficients remained relatively constant over time. However, several (not necessarily statistically significant) trends in the modification of effects over the week of admission on mortality were visible, such as a decrease over the week of the effect of obesity on mortality, elevated effect of eosinophils, and an increase over the week of the effect of elevated haemoglobin, elevated potassium and elevated oxygen saturation. Some of these might be depletion effects related to relatively high patient mortality in the first few days, for example, the apparent protective effect of obesity and high eosinophils.

Discussion

Using routinely collected data for more than a thousand patients admitted with COVID-19 at a large UK hospital trust, we have developed and externally validated prognostic models for mortality and ITU admission. The models showed good discrimination and calibration. The candidate predictors explored included a clinically informed, wider range of demographics, clinical observations, symptoms, comorbidities, biomarkers and radiological investigations than those included in the derivation of existing prognostic scores or models.

If integrated into hospital electronic medical records systems, the model algorithms will provide a predicted probability of mortality or ITU admission within 28 days of hospital admission for each patient based on their individual data at, or close to, the time of admission, which will support clinicians' decision making with regard to appropriate patient care pathways and triage. This information might also assist clinicians in explaining complex

For peer review only - http://bmjopen.bmj.com/site/about/guidelines.xhtml

prognostic assessments and decisions to patients and their relatives, particularly at a time when relatives are unable to see the patient and understand how unwell they are.

Summary of results

The models developed using all 63 available candidate predictors from UHB performed well with an optimism-adjusted AUROC of 0.779 (95 % CI 0.744-0.813) for mortality within 28 days of admission and 0.893 (95% CI 0.864-0.922) for ITU admission.

Not all variables included in the UHB dataset are routinely collected at admission in other hospitals; therefore, reduced models using only variables common to both UHB and the CovidCollab external validation dataset were explored. Discrimination remained similar, with an AUROC of 0.791 (95% CI 0.761-0.822) for mortality and 0.906 (95% CI 0.883-0.929) for ITU admission in the UHB derivation dataset. These reduced models also performed well in the CovidCollab external validation dataset, with AUROCs of 0.767 (95% CI 0.754-0.780) and 0.811 (95% CI 0.795-0.828) for mortality and ITU admission, respectively. The models also performed well in gender-stratified and age-stratified patient subgroups.

Calibration of all models showed good agreement between observed and predicted probabilities, particularly at lower predicted probabilities in the range where the models would be of most clinical utility.

We found that addition of comorbidities to the model predictors did not improve overall model performance. This may be due to a correlation between presence of comorbidities and related physiological measurements and/or biomarkers which are already captured by the model.

Comparison with existing literature

Two systematic reviews summarised the existing secondary care COVID-19 prognostic models or scores published until the 31st May 2020.[1,17] The majority of the reported models, along with several more recent ones,[18] were derived in Chinese cohorts. Many of the models included in the reviews demonstrated high discriminatory performance, however all pre-existing models when assessed using the PROBAST score were at high risk of bias. Furthermore, few models were externally validated in suitable cohorts. By deriving our model from routinely collected data, we were able to reduce the risk of bias in patient selection as well as predictor and outcome measurements. Additionally, in this study we were able to externally validate models in a large global heterogeneous cohort.

More recently, the most notable secondary care prediction model advised for uptake in UK hospitals was derived from the ISARIC-WHO collaborating cohort and has been externally validated.[5,19] Both the full and reduced UHB-derived models for mortality had slightly better discrimination than the ISARIC 4C score in the UHB data (AUROC 0.753, 95% CI 0.720-0.785 for 4C). This compares with an AUROC of 0.767 (95% CI 0.760 to 0.773) for the 4C score reported in the original ISARIC validation cohort.[5] However, better performance may be expected for models evaluated in their development dataset compared to models developed in external datasets. The newly developed UHB model offers an advantage over the ISARIC 4C model in that it utilises only routinely collected patient data recorded at

For peer review only - http://bmjopen.bmj.com/site/about/guidelines.xhtml

admission, and does not require additional assessment and recording of specific comorbidities (which are often not routinely fully recorded at the point of admission).

In our time series analysis we did not find strong evidence for trends in predictor coefficients over the first 8 days of admission, particularly for variables included in the final models, suggesting that time-dependent effects due to effect modification or selection bias in the first week are small. Another recent model derived from patients with COVID-19 in a Hong Kong hospital adopted the use of time-dependent routinely collected predictors; the model in the Hong Kong study demonstrated high discrimination, with an AUROC of 0.91 when predicting severe COVID-19 outcomes.[20] However, this model is yet to be peer-reviewed and externally validated.

Strengths and limitations

The UHB dataset represents one of the largest and most ethnically diverse patient cohorts within the UK. Additionally, as part of the early UHB response to the COVID-19 pandemic, the hospital trust ensured that, upon admission, all patients underwent a wide range of investigations to support international research efforts examining prognostic markers. This allowed us to examine a wide range of possible predictors (63 candidate predictors after exclusions). Lastly, a strength of this study was the good performance, in terms of both discrimination and calibration, of the simplified, reduced model in an externally validated cohort (CovidCollab), indicating its suitability for wider use, including potentially in LMICs.

Despite the strengths, the findings must be considered in light of the study's limitations. Although we were able to use a derivation dataset from UHB with low levels of missing data, the overall sample size was relatively small compared to that of the ISARIC study and was limited to one UK geographical location. However, we were able to externally validate the model in a larger external cohort. A second limitation was that in the external validation cohort we were unable to examine all of the predictors included in the original full UHB model due to only a reduced set of candidate predictors being available in CovidCollab. Nevertheless, the model performed well and the results suggest it may be applicable in a wide range of datasets where only a reduced set of predictor variables is available. It was not possible to carry out stratified analysis by ethnicity as, in the UHB dataset, too few patients were included in most of the strata; ethnicity data was not available in the CovidCollab dataset. Our definition of 28-day COVID-19 mortality aligns with the current technical guidance from Public Health England and the definition used by the UK government in reporting COVID-19 mortality statistics;[21,22] however, we acknowledge that this may not capture all COVID-19-related deaths, and some other studies have utilised a longer period of follow-up.[23]

Conclusion

In this paper we have described the development and external validation of novel prognostic models which predict mortality and ITU admission within 28 days of admission for patients admitted to hospital with COVID-19. The simple, reduced models used only routinely collected data gathered at admission, showed good discrimination and calibration, performed at least as well as the existing ISARIC 4C score, and performed well in a validation cohort. The models can be integrated into existing electronic medical records systems to

For peer review only - http://bmjopen.bmj.com/site/about/guidelines.xhtml

For peer review only

calculate each individual patient’s probability of death or ITU admission at the time of hospital admission. The models should be further validated to determine their applicability in other populations. In addition, implementation of the models and clinical utility should be evaluated.

For peer review only

For peer review only - http://bmjopen.bmj.com/site/about/guidelines.xhtml

Figure legend

Figure 1. Calibration plots (observed probability (y-axis) against predicted probability (x-axis)): A. University Hospitals Birmingham (UHB) derivation dataset for mortality outcome; B. UHB derivation dataset for intensive therapy unit (ITU) admission outcome; C. UHB-R derivation/train dataset reduced model for mortality outcome; D. UHB-R derivation/train dataset reduced model for ITU admission outcome; E. CovidCollab external validation dataset reduced model for mortality outcome; F. CovidCollab external validation dataset reduced model for ITU admission outcome.

Ethical approval

Ethical approval was provided by the East Midlands – Derby REC (reference: 20/EM/0158) for the PIONEER Research Database (data from University Hospitals Birmingham).

For CovidCollab data, local, regional, and national approvals were obtained from all participating sites. In the UK, this study was registered as clinical audit or service evaluation, with approval granted in line with local information governance policies, in line with assessment and guidance by the Health Research Authority. At the lead site (University Hospitals Birmingham NHS Trust) this study was registered as clinical audit (CARMS-15986). In other countries, local principal investigators were responsible for obtaining approvals in line with their local, regional, and national guidelines and recommendations. Only routinely collected data was collected and patient care was not altered by this study. Anonymised data was securely transferred to the Birmingham Centre for Prospective and Observational Studies (BiCOPS), University of Birmingham via REDCap. All sites were required to confirm that approvals were in place prior to being provided with logins; written data sharing agreements were arranged where requested by individual sites.

Contributorship statement

The study was designed by NJA, KN, ES, MP, YT, CS and TT. Data was collected by KG, ES, CS and SG. TT, NJA and DG analysed the data. The first draft was written by NJA and TT. All authors (NJA, TT, MP, CS, DG, JSC, YT, RH, IH, CW, DP, SG, KG, AKD, ES and KN) critically reviewed and revised the manuscript.

Funding

This work was funded by the Medical Research Council UK Research and Innovation (MRC UKRI) (reference COV0306). The funder had no role in developing the research question or the study protocol.

Competing interests

NJA, ES, KN, MP, AD, CS, TT and YT report a grant from UKRI MRC during the conduct of the study. ES reports grants from National Institute for Health Research (NIHR), Wellcome Trust,

For peer review only - http://bmjopen.bmj.com/site/about/guidelines.xhtml

For peer review only

MRC, Health Data Research UK (HDR-UK), British Lung Foundation, and Alpha 1 Foundation outside the submitted work. KN reports grants from MRC and HDR-UK outside the submitted work. DP reports grants from NIHR, MRC, and Chernakovsky Foundation outside the submitted work. All other authors have nothing to declare.

Transparency declaration

The lead author (the manuscript's guarantor) affirms that this manuscript is an honest, accurate, and transparent account of the study being reported; that no important aspects of the study have been omitted; and that any discrepancies from the study as planned (and, if relevant, registered) have been explained.

Exclusive licence

The Corresponding Author has the right to grant on behalf of all authors and does grant on behalf of all authors, a worldwide licence (http://www.bmj.com/sites/default/files/BMJ%20Author%20Licence%20March%202013.doc) to the Publishers and its licensees in perpetuity, in all forms, formats and media (whether known now or created in the future), to i) publish, reproduce, distribute, display and store the Contribution, ii) translate the Contribution into other languages, create adaptations, reprints, include within collections and create summaries, extracts and/or, abstracts of the Contribution and convert or allow conversion into any format including without limitation audio, iii) create any other derivative work(s) based in whole or part on the on the Contribution, iv) to exploit all subsidiary rights to exploit all subsidiary rights that currently exist or as may exist in the future in the Contribution, v) the inclusion of electronic links from the Contribution to third party material where-ever it may be located; and, vi) licence any third party to do any or all of the above.

Data sharing

No additional data available.

Dissemination declaration

Dissemination to participants is not possible as this is a review of existing evidence. Patients and the public will not be involved in dissemination of the findings.

Acknowledgements

MP and YT are supported by the NIHR Birmingham Biomedical Research Centre. The views expressed are those of the author(s) and not necessarily those of the NHS, NIHR or the Department of Health and Social Care.

We would also like to acknowledge the collaborators for the CovidCollab study: Sarah Richardson, Miles Witham, AGE Research Group, NIHR Newcastle Biomedical Research Centre, University of Newcastle and Newcastle-upon-Tyne Hospitals NHS Foundation Trust, UK; Omar A Abdelwahab, Elsayed M Awad, Ahmed Y Azzam, Ahmed Cordie, Ahmed O

For peer review only - http://bmjopen.bmj.com/site/about/guidelines.xhtml

For peer review only

Elmehrath, Mostafa El-Shazly, Almontacer EB Masood, Alazher University Hospital, Egypt; Osama MAS Abdulhadi, Hazem Ahmed, Muhammed Elhadi, Ahmed KM Hadreiez, Ahmed A Momen, Mosab AA Shaban, Alkhadra Hospital, Libya; Giuseppe Cecere, Aldo Rocca, Antonio Cardarelli Hospital, Italy; Hossam Aldein S Abd Elazeem, Mohammed H Abdelhafez, Islam A Ahmed, Shrouk M Elghazaly, Helal F Hetta, Mohamed Eltaher AA Ibrahim, Soha M Mohamed, Aliae AR Mohamed Hussein, Mohamed M Moustafa, Mariam Albatoul Nageh, Mahmoud M Saad, Alshaimaa M Saad, Omar Zein Elabedeen, Assiut University Hospital, Egypt; Victoria Cox, Danielle Hunsley, Rebecca Ryall, Kathleen T Shakespeare, Thyn Thyn, Rachael Webb, Barnsley Hospital NHS Foundation Trust, UK; Deepthy Hari Madhavan, Nik Sanyal, Birmingham Heartlands Hospital, UK; Bryony Brown, Matthew Hale, Bradford Teaching Hospitals NHS Foundation Trust, UK; Marie Goujon, Cambridge University Hospitals NHS Foundation Trust, UK; Benjamin Jelley, Cardiff University, UK; Laxmi Babar, Tina Doll, Agnieszka Felska, Daniel N Guerero, Sandeep Karthikeyan, Anne Karunatilleke, Helena Lee, Emma Livesey, Amelia Roberts, Charlotte Roberts-Rhodes, City Hospital, Birmingham, UK; Teresa Perra, Alberto Porcu, Cliniche San Pietro, A.O.U. Sassari, Italy; Antonio Buondonno, Giuseppe Cecere, Aldo Rocca, Department of Medicine and Health Science "V. Tiberio", University of Molise, Italy; Vesna Hogan, Iain Wilkinson, East Surrey Hospital, UK; Ioannis Baloyiannis, Jiannis Hajiioannou, Konstantinos Perivoliotis, George Tzovaras, General University Hospital of Larissa, Greece; Anna Fleck, Aine McGovern, Glasgow Royal Infirmary, UK; Victoria Gaunt, Gloucestershire Hospitals NHS Foundation Trust, UK; Laura Babb, Emily Bailey, Jay Darley, Ioan M Draghita, Alexander Hickman, Jason Kalloo, Akhil Kanzaria, Katy Madden, Wasim Nawaz, Ambreen Sadiq, Good Hope Hospital, UK; Rifa Cardoso, Margherita Faulkner, William Hurst, Ellen James, Aimee Leadbetter, Jordan Mayer, Tanya Robinson, Emma Stratton, Miriam Thake, Hannah Thould, Hannah Watson, Great Western Hospital, UK; Ravindra Belgamwar, Corrina Bentley, Harplands Hospital, UK; Sarah Freshwater, Health Education West Midlands, UK; Sergio DV Ruiz, Nuria M Sanz, Milagros Carrasco Prats, Pedro VF Fernández, Clara G Francés, Esther M Manuel, Miguel R Marin, Pedro L Morales, Patricia P Pérez, María V Soriano , Ismael Mora-Guzmán, Hospital General Reina Sofía, Spain; Ismael Mora-Guzmán, Hospital Santa Bárbara, Spain; Melanie Dani, Imperial College Healthcare NHS Trust, UK; Fabio Barra, Antonella Ferraiolo, Simone Ferrero, Claudio Gustavino, Chiara Kratochwila, IRCCS Ospedale Policlinico San Martino, Italy; Eric W Etchill, Alodia Gabre-Kidan, Joshua H Gray, Elliott R Haut, Harsha Malapati, Sarah F Rapaport, Kent A Stevens, Dominique Vervoort, Johns Hopkins Hospital, USA; Mohammed A Azab, King Abdullah Medical City Specialist Hospital, Saudi Arabia; Catherine Bryant, Hannah Cheney-Lowe, Catrin Cox, Andrew Crowe, Gordon Dick, Sarah Evans, Patrick CP Hogan, Kar Yee Law, Alexandra Richardson, Fabio Speranza, Kathryn Toppley, Julie Whitney, Eirene Yeung, King's College Hospital, UK; Mary Ni Lochlainn, Claire Steves, King's College London, UK; Alexandros Charalabopoulos, Spyridon Davakis, Amalia Karapanou, Theodore Liakakos, Eustratia Mpaili, Maria Mpoura, Michail A Sampanis, Nikolaos V Sipsas, Laiko University Hospital, Greece; Lucy Beishon, Elinor Burn, Parveen Doddamani, Victoria Haunton, Shahriar Kabir, Hannah Shaw, Chloe Warner, Leicester Royal Infirmary, UK; Chee Soo, Maidstone and Tunbridge Wells NHS Trust, UK; Yasmin K NasrEldin, Minia University Hospital, Egypt; Nourhan AA Ghannam, Minya General Hospital, Egypt; Isobel Sleeman, NHS Grampian, UK; Ravindra Belgamwar, Corrina Bentley, North Staffordshire Combined Healthcare NHS Trust, UK; Ali Ali, Sylvia Amini, James Belcher, Marie Giles, Hayley Jarvis, Nathan Jenko, Suvira Madan, Alexander Noar, Favour Nwolu, Jessica Parkin, Lauren C Passby, Jarita Sivam, Michael Surtees, Joanne Wagland, Ruth West, David

For peer review only - http://bmjopen.bmj.com/site/about/guidelines.xhtml

Williams, Northern General Hospital, UK; Avinash Aujayeb, Lindsey Dew, Catherine Dotchin, James M Dundas, Elinor Edwards, Georgia F Gilbert, Karl Jackson, Sarah H Manning, Dominic Maxfield, Nicholas Moss, Declan C Murphy, Ellen Tullo, Sarah H Welsh, Northumbria NHS Hospital Trust, UK; Tahir Masud, Nottingham University Hospitals NHS Trust, UK; Mustafa Alsahab, Oxford University Hospitals NHS Trust, UK; Antonio Buondonno, Enrico Pinotti, Policlinico San Pietro, Italy; Francesco Alessandri, Gioia Brachini, Giancarlo Ceccarelli, Flavia Ciccarone, Pierfranco M Cicerchia, Bruno Cirillo, Giorgio De Toma, Giulia Duranti, Enrico Fiori, Giovanni B Fonsi, Pierfrancesco Lapolla, Simona Meneghini, Andrea Mingoli, Francesco Pugliese, Paolo Sapienza, Luigi Simonelli, Martina Zambon, Policlinico Umberto I, Sapienza University of Rome, Italy; Caterina Cattel, Laurenny Guzman, Princess Royal Hospital, King's College Hospital Trust, Surrey, UK; Hannah Dowell, Aina Ibukunoluwakitan, Fawsiya Mohamed, Claire Spice, Amanda Stafford, Queen Alexandra Hospital, Portsmouth, UK; Jolene Atia, Catherine Atkin, Hannah Currie, Felicity Evison, Heena Khiroya, Zeinab Majid, Maria Qurashi, Queen Elizabeth Hospital Birmingham, UK; Siobhan Coulter, Claire McDonald, Georgina Muir, Catherine O'Mahony, Caroline Tait, Queen Elizabeth Hospital Gateshead, UK; Rowan Davies, Katie Honney, Laura Winter, Queen Elizabeth Hospital King's Lynn, UK; Olubayode Adewole, Queen's Hospital Romford, UK; Amir Abdelmalak, Mohammed Ahmad, Muhammed H Ansari, Kingsley Appiah, Rajesh Dwivedi, Hope Elrick, Hedra Ghobrial, Rosie Jackson, Sophie Jeffs, Sasha Jeyakumar, Eleanor Lunt, Bushra Muzammil, Sylvia Pytraczyk, Jonathan Sheldrake, Jennifer Smith, Hannah Tobiss, Mark Vettasseri, Ruth H Willott, Hein Zaw, Queens Medical Centre, Nottingham, UK; Katherine Patterson, Queen's University Belfast, UK; Moulinath Bannerjee, Jean Cummings, Barbara Hart, Tom Maughan, Royal Bolton Hospital, UK; Clare Baguneid, Gabrielle Budd, Lizzie Moriarty, Omoteniola Odutola, Hannah Street, Royal Derby Hospital, UK; Alexis Carr, Royal Devon and Exeter Hospital, UK; Jennifer Pigott, Royal Free London NHS Foundation Trust, UK; Sarah Baldwin, Hannah Bashir, Jake Gibbon, Amy Gray, Grace Lewis, Christina Page, Rosanna Varden, Royal Victoria Infirmary, UK; Anthony Grubb, Elizabeth Holmes, Harjinder Kainth, Natalie McNeela, Lara Reilly, Abigail Reynolds, Mark Whitsey, Royal Wolverhampton NHS Trust, UK; Mertcan Akcay, Yeşim Akdeniz, Emrah Akın, Fatih Altintoprak, Zülfü Bayhan, Recayi Capoglu, Hakan Demir, Necattin Firat, Emre Gonullu, Tarık Harmantepe, Baris Mantoglu, Ali Muhtaroglu, Merve Yigit, Yasin A Yildiz, Sakarya Faculty of Medicine, Turkey; Lobna Al-Sodani, Nicole Burden, Evelyn Charsley, Thomas Kneen, Angeline Price, Emma Swinnerton, Salford Royal Hospital, UK; Yen Nee J Bo, Hayley R Boden, Reem Bulla, Alison Eastaugh, Helena Lee, Asma Khan, Mohammed Mubin, Amelia Roberts, Anthony Umeadi, Stephanie Wallis, Megan Williamson, Yu Lelt Win, Sandwell General Hospital, UK; Eltayeb A Ahmed, Abdulmoiz Aljafari, Abdulmalek Aljafari, Abdulkader Mohammad, Sharq Alneel Hospital, Sudan; Manpreet Badh, Amy Birchenough, Nick Coulthard, Alice Devaney, Ratnam Gandhi, Katharine Hood, Samuel North, Martha Pinkney, Ellie Shaw, Elisha Whelan, Solihull Hospital, UK; Adam Seed, Southport and Ormskirk Hospital NHS Trust, UK; Gurinder Dogra, Claire Morris, Rebecca Wright, South Tyneside District Hospital, UK; Stephen Lim, Lia Orlando, Harnish Patel, Prabhleen Puri, Sing Yang Sim, Southampton General Hospital, UK; Carolyn Akladious, Gitanjali Amaratungaz, Taha Amir, Cheran Anandarajah, Rachael Anders, Sally Aziz, Anna Barnard, Monica Bawor, Laura Bremner, Hannah Bridgwater, Hejab Butt, Andra Caracostea, Theodore Chevallier, Victoria Comerford, Jack Cullen, Niamh Cunningham, Daniel Curley, Madeleine Daly, Nikhita Dattani, Benyamin Deldar, Arjun Desai, Nirali Desai, Jugdeep Dhesi, Maria Dias, Hannah C Dooley, Samiullah Dost, Hiren Dusara, Alexander Emery, Cassandra Fairhead, Antia Fernandez, Gracie Fisk, Madeleine Garner,

For peer review only

Hannah Gerretsen, Andrew Ghobrial, Zaynub Ghufoor, Deirdre Green, Charlotte Greene, Karla Griffith, Ayushi Gupta, Patrick Harrison, Aidan Haslam, Torben Heinsohn, Lindsay Hennah, Abigail Hobill, Katherine Hopkinson, Lara Howells, Nicole Hrouda, Irem Ishlek, Rishi Iyer, Nuha Kardaman, Mairead Kelly, Nicola I Kelly, Hesham Khalid, Muhammad S Khan, Haris Khan, Matthew King, Li Kok, Aneliya Kuzeva, Rebecca Lau, Gabriel Lee, Gavriella Levinson, Danielle Lis, Baguiasri Mandane, Jamie Mawhinney, Henry Maynard, Sophie Mclachlan, Michelle Metcalf, John Millwood-Hargrave, Kelvin Miu, Aaliya Mohammed, Hamilton Morrin, Stephanie Mulhern, Daniel Muller, Varun Nadkarni, Hanna Nguyen, Alice O'Docherty, Sinead O'Dwyer, Marc Osterdahl, Ismini Panayotidis, Shefali Patel, Rose Penfold, Rupini Perinpanathan, Dina Radenkovic, Thurkka Rajeswaran, Tahmina Razzak, Emily Ross-Skinner, Hazel Sanghvi, Ross Sayers, Luca Scott, Sri Sivarajan, Katharine Stambollouian, Jack Stewart, Amybel Taylor, Hrisheekesh Vaidya, Vittoria Vergani, Madiha Virk, Vaishali Vyas, Eleanor Watkins, Catherine Wilcock, Mettha Wimalasundera, Stephanie Worrall, Natalie Yeo, Humza Yusuf, St Thomas' Hospital, UK; Adam H Dyer, Cliona Ni Cheallaigh, Liam Townsend, St. James's Hospital, Ireland; Jocelyn Amer, Emily Lyon, Michael Sen, Sunderland Royal Hospital, UK; Mohammed Al-Sadawi, Adam Budzikoski, Ishmam Ibtida, Yusra Qaiser, SUNY Downstate Brooklyn, USA; Mohammad T Azam, Asad J Choudhry, William Marx, SUNY Upstate University Hospital, USA; Ahmad Bouhuwaish, Ahmed SA Taher, Tobruk Medical Center, Libya; Nikolaos Georgiou, Jade Man, Paul Reynolds, Benjaman To, Tunbridge Wells Hospital, UK; Fatma D Collins, Sharon Budd, Ellanna Griffin, Yue Guan, Deevia Hanji, Lily Lowes, Awolkhier Mohammedseid-Nurhussien, Farhana Moomo, Olebu Ogochukwu, Katie Thin, University Hospitals Coventry and Warwickshire NHS Trust, UK; Elinor Burn, University Hospitals of Leicester NHS Trust; Terry Hughes, Thomas A Jackson, Laura Magill, Lauren McCluskey, Hannah Moorey, Kelvin Okoth, Rita Perry, Michala Petitt, Thomas Pinkney, Daisy Wilson, University of Birmingham; Grace ME Pearson, University of Bristol, UK; Christopher N Osuafor, Kelli Torsney, University of Cambridge, UK; David Strain, Jane Masoli, University of Exeter, UK; Jenni Burton, Terence Quinn, University of Glasgow, UK; Lucy Beishon, University of Leicester, UK; Joanne Taylor, University of Manchester, UK; Adam Gordon, University of Nottingham, UK; Gilda De Paola, Gaetano Gallo, Giuseppe Sammarco, Giuseppina Vescio, University 'Magna Graecia' of Catanzaro, Italy; Shivam Pancholi, University of Nicosia, Cyprus; Natalie Cox, University of Southampton, UK; Rajni Lal, Western Sydney Local Health District, Australia; Rand A Hussein, Zafaraniyah General Hospital, Iraq.

For peer review only

For peer review only - http://bmjopen.bmj.com/site/about/guidelines.xhtml

Table 1. Baseline characteristics of participants admitted with COVID-19 in the derivation (University Hospitals Birmingham) and validation (CovidCollab) datasets

a. Demographic characteristics, comorbidities and symptoms

	Development cohort (UHB)			External validation cohort (CovidCollab)		
	Total	Alive	Died	Total	Alive	Died
N	1040	752	288	6099	4431	1668
Age category (years), n (%)						
<30	35 (3.4)	35 (4.7)	0 (0.0)	125 (2.0)	122 (2.8)	3 (0.2)
30-39	42 (4.0)	39 (5.2)	3 (1.0)	270 (4.4)	257 (5.8)	13 (0.8)
40-49	91 (8.8)	87 (11.6)	4 (1.4)	497 (8.1)	459 (10.4)	38 (2.3)
50-59	146 (14.0)	123 (16.4)	23 (8.0)	793 (13.0)	707 (16.0)	86 (5.2)
60-69	181 (17.4)	143 (19.0)	38 (13.2)	944 (15.5)	736 (16.6)	208 (12.5)
70-79	220 (21.2)	147 (19.5)	73 (25.3)	1325 (21.7)	891 (20.1)	434 (26.0)
80-89	214 (20.6)	124 (16.5)	90 (31.2)	1571 (25.8)	936 (21.1)	635 (38.1)
≥90	111 (10.7)	54 (7.2)	57 (19.8)	574 (9.4)	323 (7.3)	251 (15.0)
Gender (male), n (%)	589 (56.6)	423 (56.2)	166 (57.6)	3361 (55.1)	2342 (52.9)	1019 (61.1)
Ethnicity, n (%)				Not available		
White	590 (56.7)	406 (54.0)	184 (63.9)			
South Asian	127 (12.2)	95 (12.6)	32 (11.1)			
Black	68 (6.5)	46 (6.1)	22 (7.6)			
Other	255 (24.5)	205 (27.3)	50 (17.4)			
Comorbidities, n (%)						
Dementia	373 (35.9)	249 (33.1)	124 (43.1)	934 (15.3)	539 (12.2)	395 (23.7)
Cancer	135 (13.0)	90 (12.0)	45 (15.6)	649 (10.6)	409 (9.2)	240 (14.4)
Asthma	165 (15.9)	135 (18.0)	30 (10.4)	1579 (25.9)*	1125 (25.4)*	454 (27.2)*
Chronic obstructive pulmonary disease	283 (27.2)	211 (28.1)	72 (25.0)			
Sleep apnoea	49 (4.7)	36 (4.8)	13 (4.5)			
Cardiovascular disease	567 (54.5)	366 (48.7)	201 (69.8)	3033 (49.7)	1977 (44.6)	1056 (63.3)
Hypertension	661 (63.6)	449 (59.7)	212 (73.6)	Not available		
Diabetes without complications	258 (24.8)	181 (24.1)	77 (26.7)	1794 (29.4)	1229 (27.7)	565 (33.9)
Diabetes with complications	112 (10.8)	76 (10.1)	36 (12.5)	Not available		
Peptic ulcer	29 (2.8)	25 (3.3)	4 (1.4)	Not available		
Liver disease	71 (6.8)	53 (7.0)	18 (6.2)	Not available		
Rheumatic/inflammatory disease	51 (4.9)	36 (4.8)	15 (5.2)	Not available		
Thyroid disorder	107 (10.3)	75 (10.0)	32 (11.1)	Not available		
ISARIC comorbidity score				Not applicable		
0	111 (10.7)	94 (12.5)	17 (5.9)			
1	234 (22.5)	175 (23.3)	59 (20.5)			
≥2	695 (66.8)	483 (64.2)	212 (73.6)			
Symptoms, n (%)						
Breathlessness	559 (59.2)	392 (56.6)	167 (66.3)	Not available		
Chest pain	39 (4.1)	33 (4.8)	6 (2.4)	Not available		
Cough	538 (57.0)	398 (57.5)	140 (55.6)	4259 (69.8)	3110 (70.2)	1149 (68.9)
Fever	465 (49.3)	339 (49.0)	126 (50.0)	3212 (52.7)	2394 (54.0)	818 (49.0)
Headache	42 (4.4)	37 (5.3)	5 (2.0)	Not available		
Malaise	186 (19.7)	147 (21.2)	39 (15.5)	Not available		
New onset diarrhoea or vomiting	56 (5.9)	49 (7.1)	7 (2.8)	Not available		
Sputum	84 (8.9)	53 (7.7)	31 (12.3)	Not available		
Delirium	80 (8.5)	41 (5.9)	39 (15.5)	1160 (20.1)	699 (16.7)	461 (28.8)
Outcomes						
Died within 28 days of admission	288 (27.7)	0 (0.0)	288 (100.0)	1668 (27.3)	0 (0.0)	1668 (100.0)
ITU admission within 28 days	183 (17.6)	132 (17.6)	51 (17.7)	722 (11.8)	477 (10.8)	245 (14.7)

*In CovidCollab, asthma, chronic obstructive pulmonary disease and sleep apnoea were combined as 'respiratory diseases'; data on the individual conditions was not available, therefore the n (%) given is for all respiratory diseases.

For peer review only - http://bmjopen.bmj.com/site/about/guidelines.xhtml

b. Physiological measures and scores

	Development cohort (UHB)			External validation cohort (CovidCollab)		
	Total	Alive	Died	Total	Alive	Died
BMI category, kg/m²						
Underweight (<18.5)	27 (2.6)	16 (2.1)	11 (3.8)	155 (2.5)	112 (2.5)	43 (2.6)
Normal weight (18.5-24.9)	273 (26.2)	179 (23.8)	94 (32.6)	1284 (21.1)	940 (21.2)	344 (20.6)
Overweight (25-29.9)	341 (32.8)	252 (33.5)	89 (30.9)	1247 (20.4)	999 (22.5)	248 (14.9)
Obese (>30)	366 (35.2)	280 (37.2)	86 (29.9)	1180 (19.3)	940 (21.2)	240 (14.4)
Missing	33 (3.2)	25 (3.3)	8 (2.8)	2233 (36.6)	1440 (32.5)	793 (47.5)
Systolic blood pressure (mm Hg), n (%)						
<140	688 (66.2)	503 (66.9)	185 (64.2)	4051 (66.4)	2936 (66.3)	1115 (66.8)
≥140	340 (32.7)	249 (33.1)	91 (31.6)	1926 (31.6)	1414 (31.9)	512 (30.7)
Missing	12 (1.2)	0 (0.0)	12 (4.2)	122 (2.0)	81 (1.8)	41 (2.5)
Diastolic blood pressure (mm Hg), n (%)						
<90	870 (83.7)	636 (84.6)	234 (81.2)	5075 (83.2)	3654 (82.5)	1421 (85.2)
≥90	158 (15.2)	116 (15.4)	42 (14.6)	910 (14.9)	701 (15.8)	209 (12.5)
Missing	12 (1.2)	0 (0.0)	12 (4.2)	114 (1.9)	76 (1.7)	38 (2.3)
Temperature (Celsius), n (%)						
<37.8	851 (81.8)	619 (82.3)	232 (80.6)	4164 (68.3)	3025 (68.3)	1139 (68.3)
≥37.8	187 (18.0)	133 (17.7)	54 (18.8)	1805 (29.6)	1316 (29.7)	489 (29.3)
Missing	2 (0.2)	0 (0.0)	2 (0.7)	130 (2.1)	90 (2.0)	40 (2.4)
Heart rate category (beats per minute), n (%)						
<80	288 (27.7)	211 (28.1)	77 (26.7)	1654 (27.1)	1190 (26.9)	464 (27.8)
80-99	441 (42.4)	326 (43.4)	115 (39.9)	2400 (39.4)	1794 (40.5)	606 (36.3)
≥100	309 (29.7)	215 (28.6)	94 (32.6)	1935 (31.7)	1370 (30.9)	565 (33.9)
Missing	2 (0.2)	0 (0.0)	2 (0.7)	110 (1.8)	77 (1.7)	33 (2.0)
Respirations (per minute), n (%)						
<20	450 (43.3)	363 (48.3)	87 (30.2)	2249 (36.9)	1769 (39.9)	480 (28.8)
≥20	573 (55.1)	389 (51.7)	184 (63.9)	3659 (60.0)	2522 (56.9)	1137 (68.2)
Missing	17 (1.6)	0 (0.0)	17 (5.9)	191 (3.1)	140 (3.2)	51 (3.1)
Oxygen saturation (%), n (%)						
<80	9 (0.9)	4 (0.5)	5 (1.7)	158 (2.6)	71 (1.6)	87 (5.2)
80-88	47 (4.5)	17 (2.3)	30 (10.4)	443 (7.3)	230 (5.2)	213 (12.8)
89-93	173 (16.6)	110 (14.6)	63 (21.9)	1108 (18.2)	775 (17.5)	333 (20.0)
≥94	809 (77.8)	621 (82.6)	188 (65.3)	4281 (70.2)	3284 (74.1)	997 (59.8)
Missing	2 (0.2)	0 (0.0)	2 (0.7)	109 (1.8)	71 (1.6)	38 (2.3)
Partial pressure of CO_2 (kPa), n (%)						
<4.67	184 (17.7)	125 (16.6)	59 (20.5)	947 (15.5)	627 (14.2)	320 (19.2)
4.67-6.3	380 (36.5)	278 (37.0)	102 (35.4)	650 (10.7)	467 (10.5)	183 (11.0)
≥6.4	176 (16.9)	124 (16.5)	52 (18.1)	214 (3.5)	128 (2.9)	86 (5.2)
Missing	300 (28.8)	225 (29.9)	75 (26.0)	4288 (70.3)	3209 (72.4)	1079 (64.7)
Portable oxygen concentrator fraction of inspired oxygen (%), n (%)						
≤0.28	629 (60.5)	458 (60.9)	171 (59.4)	3518 (57.7)	2730 (61.6)	788 (47.2)
0.28-0.49	56 (5.4)	35 (4.7)	21 (7.3)	1132 (18.6)	794 (17.9)	338 (20.3)
≥0.5	80 (7.7)	51 (6.8)	29 (10.1)	1003 (16.4)	541 (12.2)	462 (27.7)
Missing	275 (26.4)	208 (27.7)	67 (23.3)	446 (7.3)	366 (8.3)	80 (4.8)
Chest X-ray						
Clear/unchanged	210 (20.2)	161 (21.4)	49 (17.0)	1604 (26.3)	1225 (27.6)	379 (22.7)
Local consolidation	235 (22.6)	169 (22.5)	66 (22.9)	3226 (52.9)	2313 (52.2)	913 (54.7)
Ground glass opacity/bilateral infiltrates	393 (37.8)	273 (36.3)	120 (41.7)	637 (10.4)	402 (9.1)	235 (14.1)
Other/no firm diagnosis	99 (9.5)	65 (8.6)	34 (11.8)	-	-	-
None performed/missing	103 (9.9)	84 (11.2)	19 (6.6)	632 (10.4)	491 (11.1)	141 (8.5)
Frailty score, n (%)						
1-3	376 (36.2)	321 (42.7)	55 (19.1)	1451 (23.8)	1326 (29.9)	125 (7.5)

For peer review only - http://bmjopen.bmj.com/site/about/guidelines.xhtml

4-6	277 (26.6)	179 (23.8)	98 (34.0)	2079 (34.1)	1539 (34.7)	540 (32.4)
7-9	119 (11.4)	55 (7.3)	64 (22.2)	1911 (31.3)	1146 (25.9)	765 (45.9)
Missing	268 (25.8)	197 (26.2)	71 (24.7)	658 (10.8)	420 (9.5)	238 (14.3)
Glasgow Coma Scale score, n (%)						
<15	274 (26.3)	178 (23.7)	96 (33.3)	1314 (21.5)	744 (16.8)	570 (34.2)
15	222 (21.3)	186 (24.7)	36 (12.5)	4250 (69.7)	3366 (76.0)	884 (53.0)
Missing	544 (52.3)	388 (51.6)	156 (54.2)	535 (8.8)	321 (7.2)	214 (12.8)

For peer review only

c. Laboratory test results

	Development cohort (UHB)			External validation cohort (CovidCollab)		
	Total	Alive	Died	Total	Alive	Died
eGFR (ml/min), n (%)						
<30 (Stage 4 or above)	200 (19.2)	119 (15.8)	81 (28.1)	693 (11.4)	364 (8.2)	329 (19.7)
30-59 (Stage 3)	216 (20.8)	141 (18.8)	75 (26.0)	1362 (22.3)	846 (19.1)	516 (30.9)
60-89 (Stage 2)	317 (30.5)	246 (32.7)	71 (24.7)	1825 (29.9)	1393 (31.4)	432 (25.9)
>90 (Normal or high)	259 (24.9)	215 (28.6)	44 (15.3)	1818 (29.8)	1531 (34.6)	287 (17.2)
Missing	48 (4.6)	31 (4.1)	17 (5.9)	401 (6.6)	297 (6.7)	104 (6.2)
pH, n (%)						
<7.30	64 (6.2)	39 (5.2)	25 (8.7)	416 (6.8)	234 (5.3)	182 (10.9)
7.30-7.34	88 (8.5)	64 (8.5)	24 (8.3)	530 (8.7)	351 (7.9)	179 (10.7)
7.35-7.44	429 (41.2)	313 (41.6)	116 (40.3)	2300 (37.7)	1643 (37.1)	657 (39.4)
≥7.45	152 (14.6)	107 (14.2)	45 (15.6)	960 (15.7)	725 (16.4)	235 (14.1)
Missing	307 (29.5)	229 (30.5)	78 (27.1)	1893 (31.0)	1478 (33.4)	415 (24.9)
Base excess (mmol/l), n (%)						
<-2	202 (19.4)	125 (16.6)	77 (26.7)	981 (16.1)	560 (12.6)	421 (25.2)
-2-2	349 (33.6)	262 (34.8)	87 (30.2)	1996 (32.7)	1491 (33.6)	505 (30.3)
>2	182 (17.5)	136 (18.1)	46 (16.0)	1077 (17.7)	801 (18.1)	276 (16.5)
Missing	307 (29.5)	229 (30.5)	78 (27.1)	2045 (33.5)	1579 (35.6)	466 (27.9)
Anion gap (mmol/l), n (%)				Not available		
6-15	89 (8.6)	62 (8.2)	27 (9.4)			
≥16	579 (55.7)	409 (54.4)	170 (59.0)			
Missing	372 (35.8)	281 (37.4)	91 (31.6)			
White blood cell count (10^9/l), n (%)				Not available		
<3.9	79 (7.6)	69 (9.2)	10 (3.5)			
3.9-10.8	696 (66.9)	518 (68.9)	178 (61.8)			
≥10.9	215 (20.7)	135 (18.0)	80 (27.8)			
Missing	50 (4.8)	30 (4.0)	20 (6.9)			
Platelets (10^9/l), n (%)				Not available		
<150	179 (17.2)	121 (16.1)	58 (20.1)			
150-399	728 (70.0)	538 (71.5)	190 (66.0)			
≥400	80 (7.7)	62 (8.2)	18 (6.2)			
Missing	53 (5.1)	31 (4.1)	22 (7.6)			
Lymphocytes (10^9/l), n (%)						
<1.5	801 (77.0)	572 (76.1)	229 (79.5)	4684 (76.8)	3349 (75.6)	1335 (80.0)
≥1.5	195 (18.8)	154 (20.5)	41 (14.2)	1183 (19.4)	929 (21.0)	254 (15.2)
Missing	44 (4.2)	26 (3.5)	18 (6.2)	232 (3.8)	153 (3.5)	79 (4.7)
Neutrophil:lymphocyte ratio, n (%)						
<2.21	94 (9.0)	82 (10.9)	12 (4.2)	600 (9.8)	509 (11.5)	91 (5.5)
2.21-4.82	282 (27.1)	223 (29.7)	59 (20.5)	1635 (26.8)	1341 (30.3)	294 (17.6)
>4.82	620 (59.6)	421 (56.0)	199 (69.1)	3387 (55.5)	2265 (51.1)	1122 (67.3)
Missing	44 (4.2)	26 (3.5)	18 (6.2)	477 (7.8)	316 (7.1)	161 (9.7)
Mean corpuscular volume (fl), n (%)				Not available		
<80	91 (8.8)	69 (9.2)	22 (7.6)			
80-95	782 (75.2)	578 (76.9)	204 (70.8)			
≥96	123 (11.8)	79 (10.5)	44 (15.3)			
Missing	44 (4.2)	26 (3.5)	18 (6.2)			
Red cell distribution width (%), n (%)				Not available		
<11.5	13 (1.2)	11 (1.5)	2 (0.7)			
11.5-15.4	742 (71.3)	555 (73.8)	187 (64.9)			
≥15.5	240 (23.1)	160 (21.3)	80 (27.8)			
Missing	45 (4.3)	26 (3.5)	19 (6.6)			
Monocytes (10^9/l), n (%)				Not available		
<0.2	71 (6.8)	51 (6.8)	20 (6.9)			

For peer review only

0.2-0.8	731 (70.3)	539 (71.7)	192 (66.7)			
>0.8	194 (18.7)	136 (18.1)	58 (20.1)			
Missing	44 (4.2)	26 (3.5)	18 (6.2)			
Eosinophils (10^9/l), n (%)				Not available		
≤0.4	979 (94.1)	710 (94.4)	269 (93.4)			
>0.4	17 (1.6)	16 (2.1)	1 (0.3)			
Missing	44 (4.2)	26 (3.5)	18 (6.2)			
Haemoglobin (g/l), n (%)						
<115	310 (29.8)	215 (28.6)	95 (33.0)	1454 (23.8)	975 (22.0)	479 (28.7)
115-153	582 (56.0)	434 (57.7)	148 (51.4)	3718 (61.0)	2783 (62.8)	935 (56.1)
≥154	98 (9.4)	73 (9.7)	25 (8.7)	557 (9.1)	413 (9.3)	144 (8.6)
Missing	50 (4.8)	30 (4.0)	20 (6.9)	370 (6.1)	260 (5.9)	110 (6.6)
Glucose (mmol/l), n (%)				Not available		
<7.8	585 (56.2)	441 (58.6)	144 (50.0)			
7.8-8.4	76 (7.3)	47 (6.2)	29 (10.1)			
≥8.5	295 (28.4)	201 (26.7)	94 (32.6)			
Missing	84 (8.1)	63 (8.4)	21 (7.3)			
Bicarbonate (mmol/l), n (%)						
<22	184 (17.7)	118 (15.7)	66 (22.9)	996 (16.3)	608 (13.7)	388 (23.3)
22-28	451 (43.4)	339 (45.1)	112 (38.9)	2704 (44.3)	1981 (44.7)	723 (43.3)
≥29	98 (9.4)	66 (8.8)	32 (11.1)	437 (7.2)	320 (7.2)	117 (7.0)
Missing	307 (29.5)	229 (30.5)	78 (27.1)	1962 (32.2)	1522 (34.3)	440 (26.4)
C-reactive protein (mg/l), n (%)						
<10	84 (8.1)	76 (10.1)	8 (2.8)	690 (11.3)	616 (13.9)	74 (4.4)
10-99	406 (39.0)	321 (42.7)	85 (29.5)	2735 (44.8)	2101 (47.4)	634 (38.0)
≥100	483 (46.4)	314 (41.8)	169 (58.7)	2213 (36.3)	1381 (31.2)	832 (49.9)
Missing	67 (6.4)	41 (5.5)	26 (9.0)	461 (7.6)	333 (7.5)	128 (7.7)
Albumin (g/l), n (%)				Not available		
<25	189 (18.2)	123 (16.4)	66 (22.9)			
25-34	569 (54.7)	408 (54.3)	161 (55.9)			
≥35	214 (20.6)	176 (23.4)	38 (13.2)			
Missing	68 (6.5)	45 (6.0)	23 (8.0)			
Bilirubin (µmol/l), n (%)						
<21	822 (79.0)	604 (80.3)	218 (75.7)	Not available		
≥21	151 (14.5)	104 (13.8)	47 (16.3)			
Missing	67 (6.4)	44 (5.9)	23 (8.0)			
Alanine aminotransferase (U/l), n (%)						
<55	837 (80.5)	601 (79.9)	236 (81.9)	4126 (67.7)	2986 (67.4)	1140 (68.3)
≥55	134 (12.9)	106 (14.1)	28 (9.7)	777 (12.7)	559 (12.6)	218 (13.1)
Missing	69 (6.6)	45 (6.0)	24 (8.3)	1196 (19.6)	886 (20.0)	310 (18.6)
Alkaline phosphatase (U/l), n (%)				Not available		
<130	814 (78.3)	605 (80.5)	209 (72.6)			
≥130	159 (15.3)	103 (13.7)	56 (19.4)			
Missing	67 (6.4)	44 (5.9)	23 (8.0)			
Urea (mmol/l), n (%)						
<7.8	567 (54.5)	466 (62.0)	101 (35.1)	2585 (42.4)	2122 (47.9)	463 (27.8)
≥7.8	429 (41.2)	259 (34.4)	170 (59.0)	2409 (39.5)	1399 (31.6)	1010 (60.6)
Missing	44 (4.2)	27 (3.6)	17 (5.9)	1105 (18.1)	910 (20.5)	195 (11.7)
Potassium (mmol/l), n (%)				Not available		
2.5-5.2	868 (83.5)	644 (85.6)	224 (77.8)			
≥5.3	49 (4.7)	29 (3.9)	20 (6.9)			
Missing	123 (11.8)	79 (10.5)	44 (15.3)			
Sodium (mmol/l), n (%)				Not available		
<133	146 (14.0)	105 (14.0)	41 (14.2)			
133-144	746 (71.7)	567 (75.4)	179 (62.2)			
≥145	104 (10.0)	53 (7.0)	51 (17.7)			
Missing	44 (4.2)	27 (3.6)	17 (5.9)			

For peer review only

Corrected calcium (mmol/l), n (%)				Not available		
<2.2	142 (13.7)	99 (13.2)	43 (14.9)			
2.2-2.5	767 (73.8)	577 (76.7)	190 (66.0)			
≥2.6	38 (3.7)	17 (2.3)	21 (7.3)			
Missing	93 (8.9)	59 (7.8)	34 (11.8)			
Lactate (U/l), n (%)						
≤2.2	490 (47.1)	362 (48.1)	128 (44.4)	3091 (50.7)	2327 (52.5)	764 (45.8)
>2.2	192 (18.5)	121 (16.1)	71 (24.7)	996 (16.3)	558 (12.6)	438 (26.3)
Missing	358 (34.4)	269 (35.8)	89 (30.9)	2012 (33.0)	1546 (34.9)	466 (27.9)
Haematocrit (l/l), n (%)				Not available		
<0.5	963 (92.6)	707 (94.0)	256 (88.9)			
≥0.5	33 (3.2)	19 (2.5)	14 (4.9)			
Missing	44 (4.2)	26 (3.5)	18 (6.2)			

Table 2. Area under the receiver operating characteristic curve (AUROC), calibration slope and calibration intercept for models developed in University Hospitals Birmingham data (full (UHB) and reduced (UHB-R) datasets) and externally validated in CovidCollab data, and for external validation of the ISARIC 4C score

Dataset	Outcome	AUROC (95% CI)	Calibration	
			Slope	Intercept
**Model development***				
UHB	Mortality	0.779 (0.744-0.813)	0.79 (0.64-0.94)	0.06 (-0.02-0.14)
UHB	ITU admission	0.893 (0.864-0.922)	0.91 (0.80-1.01)	0.01 (-0.05-0.07)
UHB-R†	Mortality	0.791 (0.761-0.822)	0.89 (0.81-0.97)	0.03 (-0.01-0.07)
UHB-R†	ITU admission	0.906 (0.883-0.929)	0.94 (0.84-1.04)	0.00 (-0.05-0.06)
**External validation of new model***				
CovidCollab	Mortality	0.767 (0.754-0.780)	0.85 (0.75-0.94)	0.04 (-0.01-0.09)
CovidCollab	ITU admission	0.811 (0.795-0.828)	0.95 (0.82-1.08)	0.00 (-0.07-0.07)
**External validation of ISARIC 4C score in UHB data**				
UHB – 4C score	Mortality	0.753 (0.720-0.785)	0.99 (0.85-1.12)	0.00 (-0.06-0.06)

*Models derived using logistic regression with stepwise selection of candidate predictors and categorisation of continuous variables into clinically meaningful categories (after imputing missing data).

†Not all variables included in the full University Hospital Birmingham (UHB) model were available in the CovidCollab dataset. Therefore, revised (reduced) models were developed in UHB data using a subset of the candidate predictors common to both the UHB and CovidCollab datasets (UHB-R), and these were then externally validated in the CovidCollab dataset.

For peer review only

For peer review only - http://bmjopen.bmj.com/site/about/guidelines.xhtml

Table 3a. Sensitivity, specificity, positive predictive value (PPV) and negative predictive value (NPV) for mortality at 28 days after admission (University Hospitals Birmingham derivation dataset)

Predicted probability	TP	TN	FP	FN	Sensitivity (95% CI)	Specificity (95% CI)	PPV (95% CI)	NPV (95% CI)
10%	270	275	477	18	0.938 (0.924-0.953)	0.365 (0.346-0.385)	0.362 (0.352-0.371)	0.939 (0.924-0.954)
20%	238	437	315	50	0.828 (0.806-0.850)	0.581 (0.547-0.614)	0.431 (0.405-0.456)	0.898 (0.881-0.915)
30%	194	542	210	94	0.672 (0.646-0.699)	0.721 (0.696-0.746)	0.480 (0.458-0.502)	0.852 (0.841-0.862)
40%	156	622	130	132	0.540 (0.515-0.565)	0.827 (0.813-0.842)	0.545 (0.523-0.567)	0.825 (0.817-0.832)
50%	116	678	74	172	0.404 (0.366-0.442)	0.902 (0.888-0.916)	0.613 (0.578-0.648)	0.798 (0.788-0.808)
60%	82	712	40	206	0.284 (0.250-0.318)	0.947 (0.940-0.954)	0.673 (0.641-0.704)	0.775 (0.768-0.783)
70%	50	731	21	238	0.174 (0.147-0.200)	0.972 (0.966-0.978)	0.706 (0.671-0.741)	0.754 (0.749-0.760)
80%	24	741	11	264	0.083 (0.073-0.094)	0.986 (0.979-0.992)	0.691 (0.576-0.807)	0.737 (0.734-0.740)
90%	8	750	2	280	0.026 (0.015-0.038)	0.997 (0.996-0.998)	0.772 (0.663-0.881)	0.728 (0.726-0.730)

TP = true positives, TN = true negatives, FP = false positives, FN = false negatives.

For peer review only

Table 3b. Sensitivity, specificity, positive predictive value (PPV) and negative predictive value (NPV) for intensive therapy unit admission within 28 days after admission (University Hospitals Birmingham derivation dataset)

Predicted probability	TP	TN	FP	FN	Sensitivity (95% CI)	Specificity (95% CI)	PPV (95% CI)	NPV (95% CI)
10%	161	617	240	22	0.878 (0.851-0.905)	0.720 (0.694-0.746)	0.401 (0.373-0.430)	0.965 (0.956-0.974)
20%	144	709	148	39	0.789 (0.743-0.835)	0.827 (0.814-0.841)	0.494 (0.464-0.523)	0.948 (0.937-0.959)
30%	129	771	86	54	0.707 (0.662-0.752)	0.900 (0.891-0.908)	0.601 (0.575-0.626)	0.935 (0.926-0.944)
40%	115	797	60	68	0.631 (0.597-0.664)	0.930 (0.925-0.935)	0.659 (0.634-0.684)	0.922 (0.915-0.929)
50%	97	817	40	86	0.532 (0.502-0.563)	0.954 (0.952-0.956)	0.711 (0.695-0.727)	0.905 (0.900-0.911)
60%	81	831	26	102	0.445 (0.429-0.461)	0.970 (0.963-0.977)	0.760 (0.722-0.798)	0.891 (0.889-0.893)
70%	67	841	16	116	0.367 (0.345-0.390)	0.982 (0.975-0.989)	0.812 (0.758-0.867)	0.879 (0.876-0.882)
80%	55	848	9	128	0.303 (0.276-0.330)	0.989 (0.985-0.994)	0.860 (0.806-0.915)	0.869 (0.865-0.874)
90%	35	854	3	148	0.190 (0.141-0.240)	0.997 (0.993-1)	0.924 (0.848-1)	0.852 (0.844-0.860)

TP = true positives, TN = true negatives, FP = false positives, FN = false negatives.

For peer review only

Table 4a. Sensitivity, specificity, positive predictive value (PPV) and negative predictive value (NPV) for mortality at 28 days after admission for the reduced model (University Hospitals Birmingham derivation dataset and CovidCollab external validation dataset, using predictors common to both datasets)

Predicted probability	TP	TN	FP	FN	Sensitivity (95% CI)	Specificity (95% CI)	PPV (95% CI)	NPV (95% CI)
**UHB-R derivation dataset (reduced model)**								
10%	278	249	503	10	0.964 (0.955-0.972)	0.332 (0.315-0.348)	0.356 (0.351-0.360)	0.960 (0.952-0.968)
20%	248	406	346	40	0.862 (0.847-0.878)	0.539 (0.510-0.569)	0.418 (0.403-0.433)	0.911 (0.902-0.920)
30%	202	536	216	86	0.703 (0.671-0.734)	0.713 (0.698-0.727)	0.484 (0.464-0.504)	0.862 (0.849-0.876)
40%	153	635	117	135	0.532 (0.511-0.553)	0.844 (0.831-0.858)	0.567 (0.547-0.587)	0.825 (0.819-0.831)
50%	104	686	66	184	0.360 (0.335-0.384)	0.913 (0.905-0.920)	0.612 (0.593-0.632)	0.788 (0.782-0.794)
60%	66	723	29	222	0.228 (0.211-0.246)	0.962 (0.953-0.970)	0.696 (0.663-0.729)	0.765 (0.762-0.768)
70%	35	744	8	253	0.122 (0.101-0.144)	0.990 (0.985-0.994)	0.819 (0.753-0.884)	0.746 (0.742-0.751)
80%	14	750	2	274	0.050 (0.036-0.064)	0.997 (0.996-0.998)	0.868 (0.835-0.900)	0.733 (0.730-0.735)
90%	3	752	0	285	0.010 (0-0.022)	0.999 (0.998-1)	0.900 (0.573-1)	0.725 (0.723-0.727)
**CovidCollab external validation dataset**								
10%	1614	1218	3213	54	0.967 (0.962-0.973)	0.275 (0.260-0.290)	0.334 (0.330-0.338)	0.957 (0.951-0.963)
20%	1471	2028	2403	197	0.882 (0.874-0.889)	0.458 (0.445-0.471)	0.380 (0.375-0.384)	0.911 (0.907-0.916)
30%	1289	2721	1710	379	0.773 (0.763-0.782)	0.614 (0.602-0.626)	0.430 (0.425-0.435)	0.878 (0.874-0.881)
40%	1059	3264	1167	609	0.635 (0.605-0.665)	0.737 (0.717-0.756)	0.476 (0.469-0.483)	0.843 (0.835-0.851)
50%	850	3698	733	818	0.510 (0.470-0.549)	0.835 (0.817-0.852)	0.537 (0.523-0.551)	0.819 (0.810-0.828)
60%	599	4025	406	1069	0.359 (0.305-0.414)	0.908 (0.890-0.927)	0.596 (0.578-0.615)	0.790 (0.779-0.801)
70%	380	4222	209	1288	0.228 (0.186-0.270)	0.953 (0.940-0.965)	0.646 (0.626-0.666)	0.766 (0.759-0.774)
80%	174	4355	76	1494	0.104 (0.077-0.132)	0.983 (0.978-0.987)	0.697 (0.671-0.722)	0.745 (0.740-0.750)
90%	37	4414	17	1631	0.022 (0.006-0.039)	0.996 (0.995-0.998)	0.676 (0.572-0.780)	0.730 (0.727-0.733)

TP = true positives, TN = true negatives, FP = false positives, FN = false negatives.

For peer review only - http://bmjopen.bmj.com/site/about/guidelines.xhtml

Table 4b. Sensitivity, specificity, positive predictive value (PPV) and negative predictive value (NPV) for intensive therapy unit admission within 28 days after admission in the reduced model (University Hospitals Birmingham derivation dataset and CovidCollab external validation dataset, using predictors common to both datasets)

Predicted probability	TP	TN	FP	FN	Sensitivity (95% CI)	Specificity (95% CI)	PPV (95% CI)	NPV (95% CI)
**UHB-R derivation dataset (reduced model)**								
10%	165	590	267	18	0.904 (0.877-0.931)	0.689 (0.671-0.707)	0.383 (0.368-0.398)	0.971 (0.963-0.979)
20%	152	709	148	31	0.831 (0.812-0.849)	0.827 (0.816-0.839)	0.507 (0.494-0.519)	0.958 (0.954-0.962)
30%	132	765	92	51	0.723 (0.698-0.749)	0.893 (0.882-0.904)	0.591 (0.567-0.615)	0.938 (0.933-0.943)
40%	112	803	54	71	0.613 (0.573-0.653)	0.937 (0.928-0.946)	0.675 (0.649-0.702)	0.919 (0.912-0.926)
50%	92	829	28	91	0.505 (0.483-0.526)	0.967 (0.956-0.979)	0.768 (0.708-0.829)	0.901 (0.898-0.905)
60%	77	841	16	106	0.419 (0.403-0.435)	0.981 (0.974-0.988)	0.828 (0.779-0.877)	0.888 (0.886-0.890)
70%	67	847	10	116	0.365 (0.344-0.386)	0.988 (0.983-0.994)	0.870 (0.816-0.924)	0.879 (0.876-0.883)
80%	46	851	6	137	0.251 (0.189-0.313)	0.993 (0.989-0.997)	0.889 (0.840-0.938)	0.861 (0.852-0.871)
90%	20	855	2	163	0.108 (0.067-0.149)	0.997 (0.996-0.999)	0.898 (0.838-0.957)	0.840 (0.833-0.846)
**CovidCollab external validation dataset**								
10%	574	3623	1754	148	0.794 (0.764-0.825)	0.674 (0.651-0.697)	0.247 (0.238-0.255)	0.961 (0.956-0.965)
20%	465	4312	1065	257	0.644 (0.621-0.667)	0.802 (0.785-0.819)	0.304 (0.288-0.321)	0.944 (0.941-0.947)
30%	377	4655	722	345	0.523 (0.497-0.549)	0.866 (0.847-0.885)	0.344 (0.320-0.367)	0.931 (0.929-0.933)
40%	315	4881	496	407	0.437 (0.381-0.492)	0.908 (0.887-0.928)	0.389 (0.361-0.417)	0.923 (0.918-0.929)
50%	250	5045	332	472	0.346 (0.258-0.434)	0.938 (0.920-0.956)	0.430 (0.413-0.447)	0.914 (0.905-0.923)
60%	178	5168	209	544	0.247 (0.175-0.318)	0.961 (0.945-0.977)	0.462 (0.420-0.503)	0.905 (0.898-0.912)
70%	113	5256	121	609	0.157 (0.105-0.208)	0.978 (0.967-0.988)	0.486 (0.439-0.534)	0.896 (0.891-0.901)
80%	57	5319	58	665	0.079 (0.044-0.114)	0.989 (0.984-0.995)	0.500 (0.410-0.590)	0.889 (0.886-0.892)
90%	18	5357	20	704	0.025 (0.006-0.044)	0.996 (0.993-1)	0.485 (0.367-0.603)	0.884 (0.882-0.886)

TP = true positives, TN = true negatives, FP = false positives, FN = false negatives.

For peer review only - http://bmjopen.bmj.com/site/about/guidelines.xhtml

References

[1] Wynants L, Van Calster B, Bonten MMJ, et al. Prediction models for diagnosis and prognosis of covid-19 infection: systematic review and critical appraisal. BMJ 2020;369:m1328. doi:10.1136 bmj.m1328
[2] Carr E, Bendayan R, Bean D, et al. Evaluation and improvement of the National Early Warning Score (NEWS2) for COVID-19: a multi-hospital study. BMC Medicine 2021;19. doi:10.1186/s12916-020-01893-3
[3] Covino M, Sandroni C, Santoro M, et al. Predicting intensive care unit admission and death for COVID-19 patients in the emergency department using early warning scores. Resuscitation 2020;156:84-91. doi:10.1016/j.resuscitation.2020.08.124
[4] Liang W, et al. Development and Validation of a Clinical Risk Score to Predict the Occurrence of Critical Illness in Hospitalized Patients with COVID-19. JAMA Intern Med 2020. doi:10.1001/jamainternmed.2020.2033
[5] Knight SR, Ho A, Pius R et al. Risk stratification of patients admitted to hospital with covid-19 using the ISARIC WHO Clinical Characterisation Protocol: development and validation of the 4C Mortality Score. BMJ 2020;370:m3339 doi:10.1136/bmj.m3339
[6] Hua-Gen Li M, Hutchinson A, Tacey M, Duke G. Reliability of comorbidity scores derived from administrative data in the tertiary hospital intensive care setting: a cross-sectional study BMJ Health & Care Informatics 2019;26:e000016. doi:10.1136/bmjhci-2019-000016
[7] Reynolds A, Seed A, Khan A, et al. on behalf of NIHR Clinical Research Network West Midlands, Birmingham Surgical Trials Consortium, and Geriatric Medicine Research Collaborative. CovidCollab. A panspecialty international project to determine point of care predictors from routinely collected data. Protocol v 1.4. 2020. Available at: https://www.bgs.org.uk/sites/default/files/content/attachment/2020-04-03/CovidCollab%20Main%20Study%20Protocol%20v2.pdf [accessed 26th Oct 2020].
[8] Geriatric Medicine Research Collaborative, Covid Collaborative, Carly Welch. Age and Frailty Are Independently Associated with Increased Mortality in COVID-19: Results of an International Multi-Centre Study. Preprint available at: https://ssrn.com/abstract=3709847, doi:10.2139/ssrn.3709847 [accessed 9th December 2020]
[9] Zhang Z. Variable selection with stepwise and best subset approaches. Ann Transl Med 2016;4(7):136. doi:10.21037/atm.2016.03.35
[10] Freund Y, Schapire RE. A decision-theoretic generalization of on-line learning and an application to boosting. Journal of Computer and System Sciences 1997;55(1):119-139.
[11] Mandrekar JN. Receiver operating characteristic curve in diagnostic test assessment. J Thorac Oncol 2010;5:1315-6. doi:10.1097/JTO.0b013e3181ec173d
[12] Rubin DB (ed.). Multiple imputation for nonresponse in surveys. 1987. doi:10.1002/9780470316696
[13] White IR, Royston P, Wood AM. Multiple imputation using chained equations: Issues and guidance for practice. Statistics in Medicine 2011;30:377-399.
[14] Rockwood K, Song X, MacKnight C, Bergman H, Hogan DB, McDowell I, Mitnitski A. A global clinical measure of fitness and frailty in elderly people. CMAJ. 2005;173(5):489-95. doi:10.1503/cmaj.050051
[15] Teasdale G, Jennett B. Assessment and prognosis of coma after head injury. Acta Neurochirurgica 1976;34:45-55. doi:10.1007/BF01405862
[16] Institute of Neurological Sciences NHS Greater Glasgow and Clyde. Glasgow Coma Scale: Do it this way. 2015. Royal College of Physicians and Surgeons of Glasgow: Glasgow. Available at: https://glasgowcomascale.org/downloads/GCS-Assessment-Aid-English.pdf?v=3 [accessed 9th December 2020].
[17] Katzenschlager S, Zimmer AJ, Gottschalk C, et al. Can we predict the severe course of COVID-19 – a systematic review and meta-analysis of indicators of clinical outcome? medRxiv preprint 2020. doi:10.1101/2020.11.09.20228858
[18] Liang W, Liang H, Ou L, et al. Development and Validation of a Clinical Risk Score to Predict the Occurrence of Critical Illness in Hospitalized Patients With COVID-19. JAMA Intern Med 2020;180:1081-1089.
[19] Covino M, De Matteis G, Burzo ML, et al. GEMELLI AGAINST COVID-19 Group. Predicting In-Hospital Mortality in COVID-19 Older Patients with Specifically Developed Scores. J Am Geriatr Soc 2020;69:37-43. doi:10.1111/jgs.16956
[20] Zhou J, Lee S, Wang X, et al. Development of a predictive risk model for severe COVID-19 disease using population-based administrative data. medRxiv preprint 2020. doi:10.1101/2020.10.21.20217380

For peer review only - http://bmjopen.bmj.com/site/about/guidelines.xhtml

[21] Public Health England. Technical summary. Public Health England data series on deaths in people with COVID-19. 2020. Available at: https://assets.publishing.service.gov.uk/government/uploads/system/uploads/attachment_data/file/916035/RA_Technical_Summary_-_PHE_Data_Series_COVID_19_Deaths_20200812.pdf [accessed 18th June 2021]

[22] Public Health England. Coronavirus (COVID-19) in the UK. Deaths in United Kingdom. 2021. Available at: https://coronavirus.data.gov.uk/details/deaths [accessed 11th June 2021]

[23] Aliberti MJR, Covinsky KE, Garcez FB, et al. A fuller picture of COVID-19 prognosis: the added value of vulnerability measures to predict mortality in hospitalised older adults. Age Ageing. 2021 Jan 8;50:32-39. doi:10.1093/ageing/afaa240

For peer review only

For peer review only - http://bmjopen.bmj.com/site/about/guidelines.xhtml

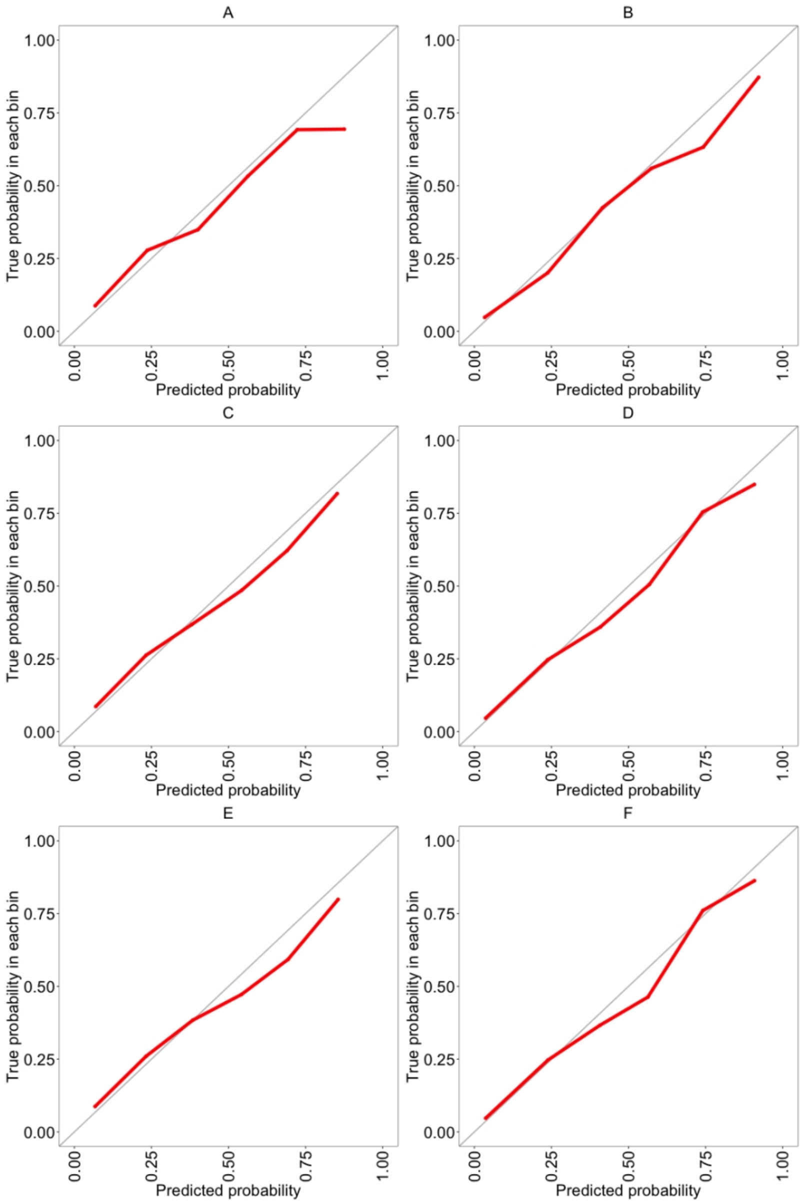

Figure 1. Calibration plots (observed probability (y-axis) against predicted probability (x-axis)): A. University Hospitals Birmingham (UHB) derivation dataset for mortality outcome; B. UHB derivation dataset for intensive therapy unit (ITU) admission outcome; C. UHB-R derivation/train dataset reduced model for mortality outcome; D. UHB-R derivation/train dataset reduced model for ITU admission outcome; E. CovidCollab external validation dataset reduced model for mortality outcome; F. CovidCollab external validation dataset reduced model for ITU admission outcome.

84x127mm (300 x 300 DPI)

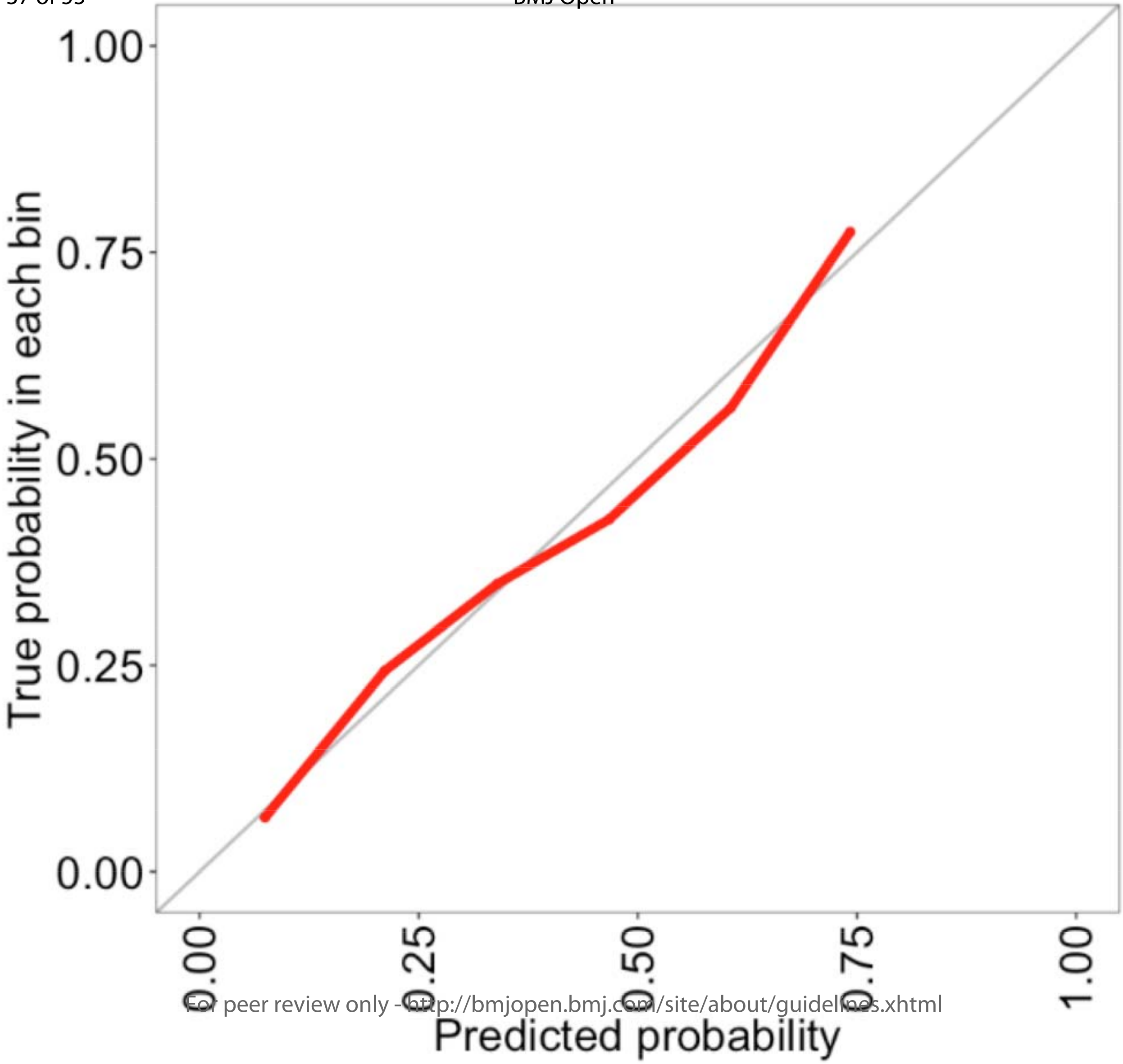

For peer review only - http://bmjopen.bmj.com/site/about/guidelines.xhtml

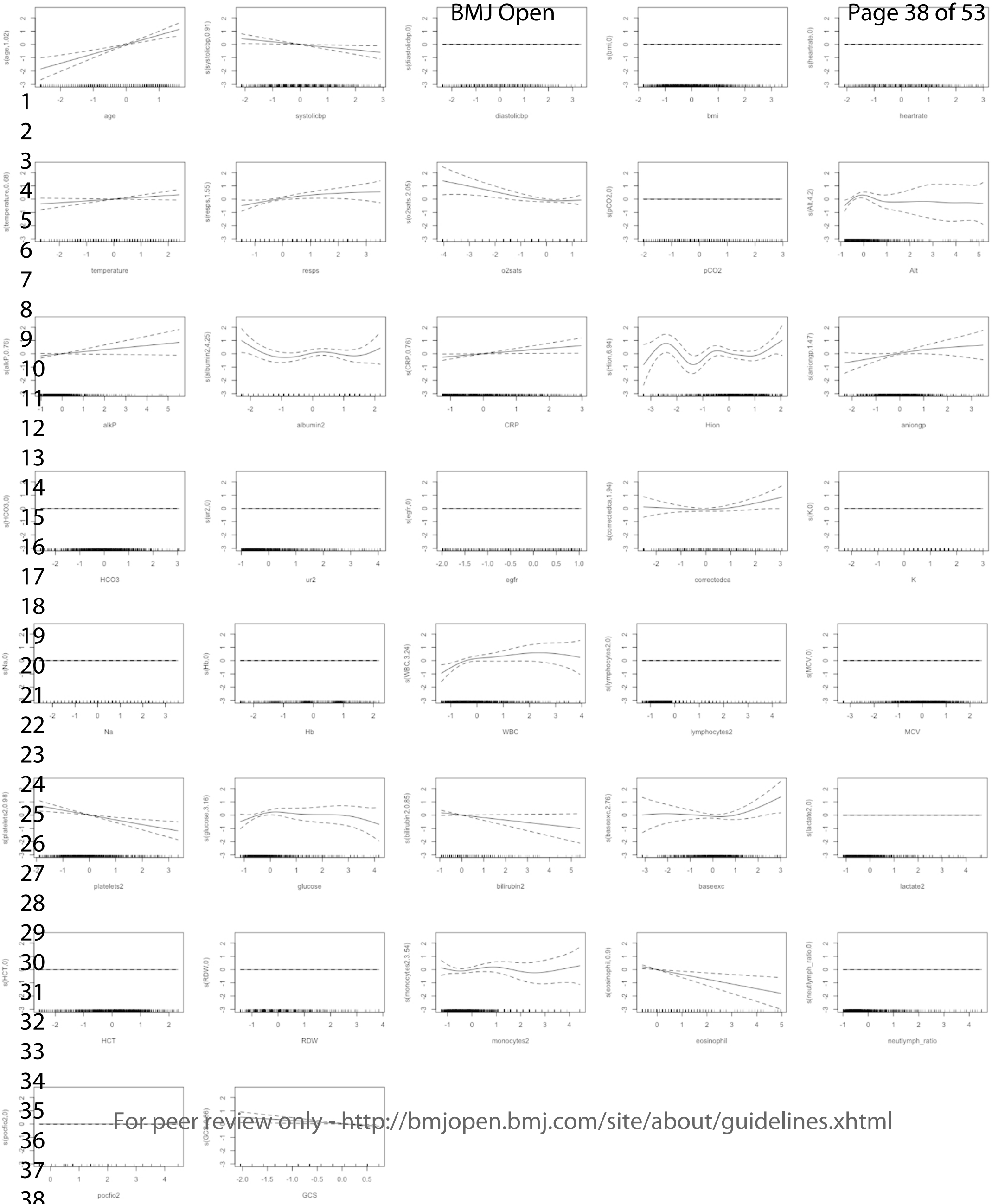
s(age,1.02)
age
s(systolicbp,0.91)
systolicbp
s(diastolicbp,0)
diastolicbp
s(bmi,0)
bmi
s(heartrate,0)
heartrate
s(temperature,0.68)
temperature
s(resps,1.55)
resps
s(o2sats,2.05)
o2sats
s(pCO2,0)
pCO2
s(Alt,4.2)
Alt
s(alkP,0.76)
alkP
s(albumin2,4.25)
albumin2
s(CRP,0.76)
CRP
s(Hion,6.94)
Hion
s(aniongp,1.47)
aniongp
s(HCO3,0)
HCO3
s(ur2,0)
ur2
s(egfr,0)
egfr
s(correctedca,1.94)
correctedca
s(K,0)
K
s(Na,0)
Na
s(Hb,0)
Hb
s(WBC,3.24)
WBC
s(lymphocytes2,0)
lymphocytes2
s(MCV,0)
MCV
s(platelets2,0.98)
platelets2
s(glucose,3.16)
glucose
s(bilirubin2,0.85)
bilirubin2
s(baseexc,2.76)
baseexc
s(lactate2,0)
lactate2
s(HCT,0)
HCT
s(RDW,0)
RDW
s(monocytes2,3.54)
monocytes2
s(eosinophil,0.9)
eosinophil
s(neutlymph_ratio,0)
neutlymph_ratio
s(pocfio2,0)
pocfio2
GCS

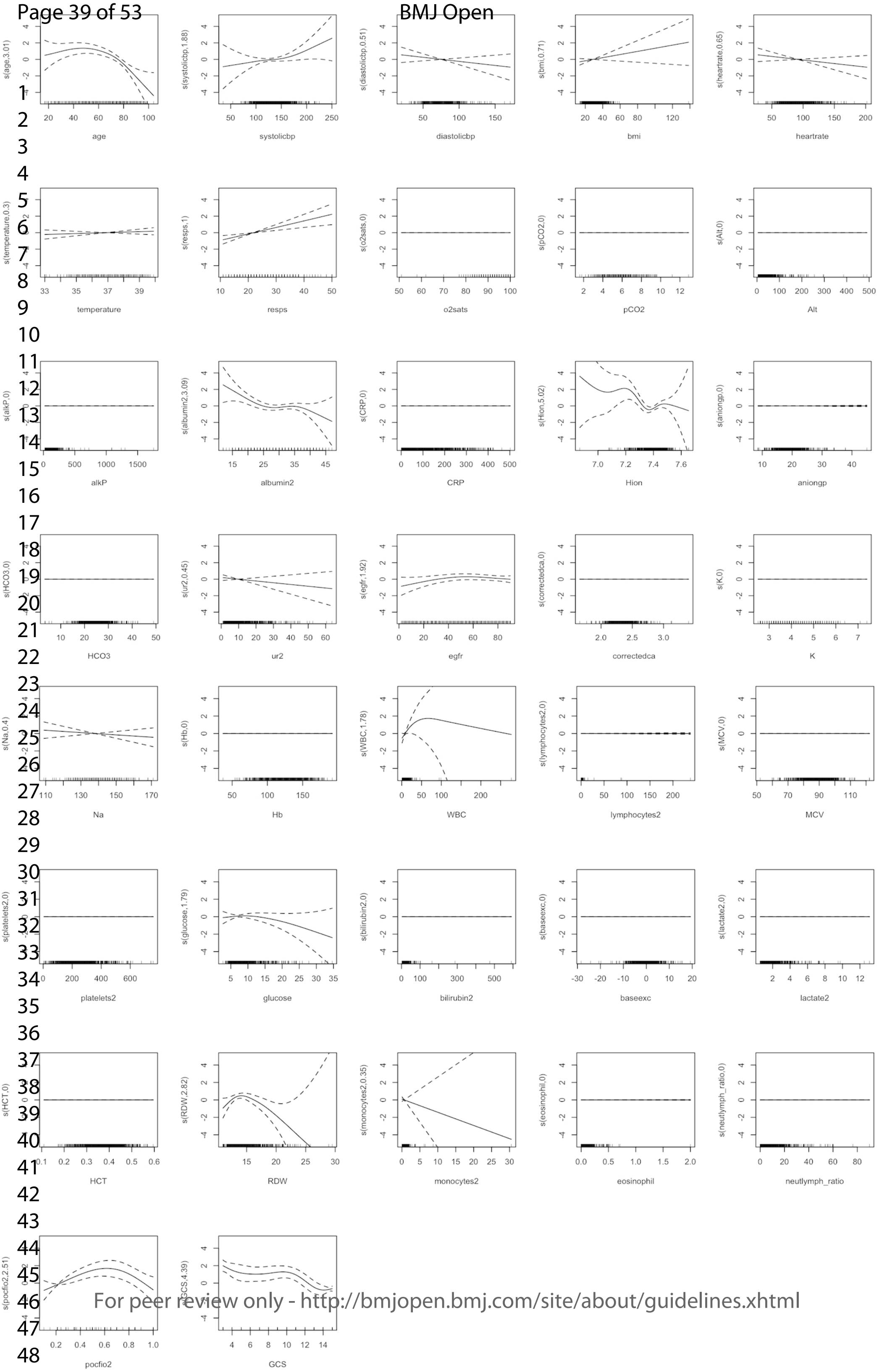
s(age,3.01)
age
s(systolicbp,1.88)
systolicbp
s(diastolicbp,0.51)
diastolicbp
s(bmi,0.71)
bmi
s(heartrate,0.65)
heartrate
s(temperature,0.3)
temperature
s(resps,1)
resps
s(o2sats,0)
o2sats
s(pCO2,0)
pCO2
s(Alt,0)
Alt
s(alkP,0)
alkP
s(albumin2,3.09)
albumin2
s(CRP,0)
CRP
s(Hion,5.02)
Hion
s(aniongp,0)
aniongp
s(HCO3,0)
HCO3
s(ur2,0.45)
ur2
s(egfr,1.92)
egfr
s(correctedca,0)
correctedca
s(K,0)
K
s(Na,0.4)
Na
s(Hb,0)
Hb
s(WBC,1.78)
WBC
s(lymphocytes2,0)
lymphocytes2
s(MCV,0)
MCV
s(platelets2,0)
platelets2
s(glucose,1.79)
glucose
s(bilirubin2,0)
bilirubin2
s(baseexc,0)
baseexc
s(lactate2,0)
lactate2
s(HCT,0)
HCT
s(RDW,2.82)
RDW
s(monocytes2,0.35)
monocytes2
s(eosinophil,0)
eosinophil
s(neutlymph_ratio,0)
neutlymph_ratio
s(pocfio2,2.51)
pocfio2
s(GCS,4.39)
GCS

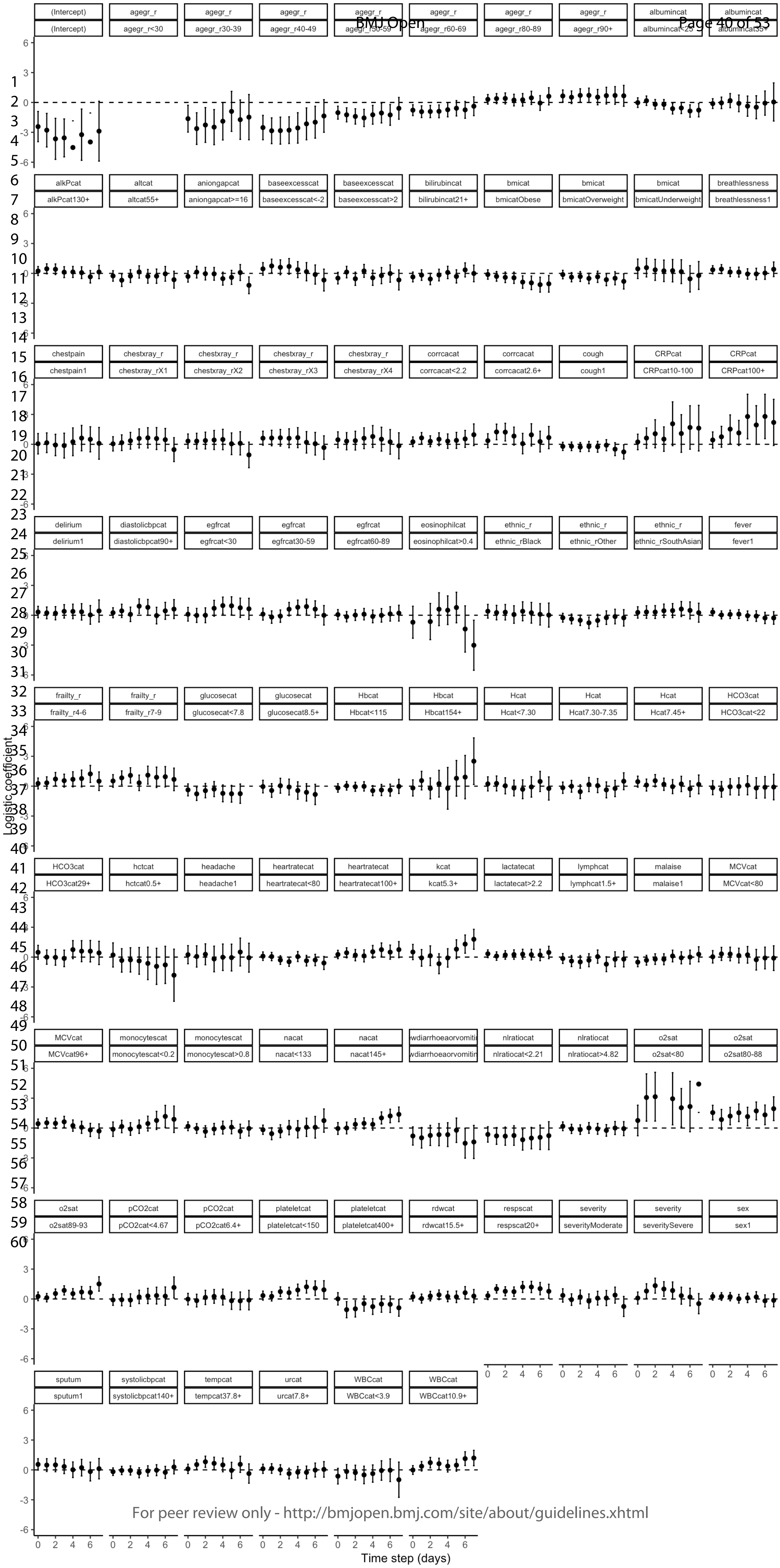
(Intercept) (Intercept)
agegr_r agegr_r<30
agegr_r agegr_r30-39
agegr_r agegr_r40-49
agegr_r agegr_r50-59
agegr_r agegr_r60-69
agegr_r agegr_r80-89
agegr_r agegr_r90+
albumincat albumincat<25
albumincat albumincat35+
alkPcat alkPcat130+
altcat altcat55+
aniongapcat aniongapcat>=16
baseexcesscat baseexcesscat<-2
baseexcesscat baseexcesscat>2
bilirubincat bilirubincat21+
bmicat bmicatObese
bmicat bmicatOverweight
bmicat bmicatUnderweight
breathlessness breathlessness1
chestpain chestpain1
chestxray_r chestxray_rX1
chestxray_r chestxray_rX2
chestxray_r chestxray_rX3
chestxray_r chestxray_rX4
corrcacat corrcacat<2.2
corrcacat corrcacat2.6+
cough cough1
CRPcat CRPcat10-100
CRPcat CRPcat100+
delirium delirium1
diastolicbpcat diastolicbpcat90+
egfrcat egfrcat<30
egfrcat egfrcat30-59
egfrcat egfrcat60-89
eosinophilcat eosinophilcat>0.4
ethnic_r ethnic_rBlack
ethnic_r ethnic_rOther
ethnic_r ethnic_rSouthAsian
fever fever1
frailty_r frailty_r4-6
frailty_r frailty_r7-9
glucosecat glucosecat<7.8
glucosecat glucosecat8.5+
Hbcat Hbcat<115
Hbcat Hbcat154+
Hcat Hcat<7.30
Hcat Hcat7.30-7.35
Hcat Hcat7.45+
HCO3cat HCO3cat<22
HCO3cat HCO3cat29+
hctcat hctcat0.5+
headache headache1
heartratecat heartratecat<80
heartratecat heartratecat100+
kcat kcat5.3+
lactatecat lactatecat>2.2
lymphcat lymphcat1.5+
malaise malaise1
MCVcat MCVcat<80
MCVcat MCVcat96+
monocytescat monocytescat<0.2
monocytescat monocytescat>0.8
nacat nacat<133
nacat nacat145+
nlratiocat nlratiocat<2.21
nlratiocat nlratiocat>4.82
o2sat o2sat<80
o2sat o2sat80-88
o2sat o2sat89-93
pCO2cat pCO2cat<4.67
pCO2cat pCO2cat6.4+
plateletcat plateletcat<150
plateletcat plateletcat400+
rdwcat rdwcat15.5+
respscat respscat20+
severity severityModerate
severity severitySevere
sex sex1
sputum sputum1
systolicbpcat systolicbpcat140+
tempcat tempcat37.8+
urcat urcat7.8+
WBCcat WBCcat<3.9
WBCcat WBCcat10.9+
Logistic coefficient
6
3
0
-3
-6
0 2 4 6
Time step (days)

Supplementary Appendix 1. Methods

Continuous variables with non-linear associations

To represent continuous variables with non-linear relationships to the logit of the outcome, we initially fitted an exploratory predictive model (gam from the mgcv package in R), representing continuous features as thin-plate smoothed splines with the smoothing factor (gamma) set to 1.4 to shrink coefficient degrees of freedom. Based on visual inspection for obvious departure for linearity, if the variable appeared to have a linear relationship with the response term, we represented it as a linear term in variable selection and model testing and training. If the relationship between the response and the variable of interest appeared to be non-linear, we represented the feature as a spline term ("bs" from splines package) with the minimum degrees of freedom (3 d.f.) to avoid overfitting.

Supplementary Appendix 2: Results

28-day mortality outcome: UHB model – analyses exploring different ways of handling of missing data

We used different methods of handling missing data in order to explore the impact on the model performance: in primary analysis, missing values were imputed; in secondary analysis, missing categories were used. For the 28-day mortality outcome, the model derived using logistic stepwise selection using categorised variables with a missing category (rather than imputed data) gave a slightly higher AUROC (0.805, 95% CI 0.776-0.835); however, it was felt the inclusion of missing categories rather than imputed values might impact generalisability of the model outside the UHB/derivation data context. Other statistical modelling techniques (LASSO, GBM) offered no improvement in model performance and the models would be more difficult to implement in clinical practice. Using continuous predictors modelled using splines where non-linear relationship with the outcome was observed (Supplementary Figure 2) rather than categorising offered a small improvement in model performance (AUROC 0.792, 95% CI 0.762-0.822), but in the interests of clinical utility and interpretability the categorical model was favoured.

ITU admission: UHB model – analyses exploring different ways of handling of missing data

Again, for the ITU admission outcome, use of a missing category rather than imputed missing values improved model performance but was not selected as the final model due to potential problems with generalisability (AUROC 0.960, 95% CI 0.944-0.976). Models using continuous rather than categorical predictors (with splines where relevant; Supplementary Figure 3) gave a small improvement in AUROC (0.910, 95% CI 0.886-0.933), but were deemed less practical.

For peer review only

Supplementary Table 1. Baseline demographic characteristics, comorbidities and symptoms of participants admitted with COVID-19 in the derivation (University Hospitals Birmingham) and validation (CovidCollab) datasets stratified by intensive therapy unit admission

	Development cohort (UHB)			External validation cohort (CovidCollab)		
	Total	Not admitted to ITU	Admitted to ITU	Total	Not admitted to ITU	Admitted to ITU
N	1040	857	183	6099	5377	722
Age category (years), n (%)						
<30	35 (3.4)	29 (3.4)	6 (3.3)	125 (2.0)	102 (1.9)	23 (3.2)
30-39	42 (4.0)	29 (3.4)	13 (7.1)	270 (4.4)	228 (4.2)	42 (5.8)
40-49	91 (8.8)	63 (7.4)	28 (15.3)	497 (8.1)	388 (7.2)	109 (15.1)
50-59	146 (14.0)	90 (10.5)	56 (30.6)	793 (13.0)	615 (11.4)	178 (24.7)
60-69	181 (17.4)	136 (15.9)	45 (24.6)	944 (15.5)	751 (14.0)	193 (26.7)
70-79	220 (21.2)	189 (22.1)	31 (16.9)	1325 (21.7)	1185 (22.0)	140 (19.4)
80-89	214 (20.6)	210 (24.5)	4 (2.2)	1571 (25.8)	1535 (28.5)	36 (5.0)
≥90	111 (10.7)	111 (13.0)	0 (0.0)	574 (9.4)	573 (10.7)	1 (0.1)
Gender (male), n (%)	589 (56.6)	456 (53.2)	133 (72.7)	3361 (55.1)	2877 (53.5)	484 (67.0)
Ethnicity, n (%)				Not available		
White	590 (56.7)	519 (60.6)	71 (38.8)			
South Asian	127 (12.2)	99 (11.6)	28 (15.3)			
Black	68 (6.5)	56 (6.5)	12 (6.6)			
Other	255 (24.5)	183 (21.4)	72 (39.3)			
Comorbidities, n (%)						
Dementia	373 (35.9)	297 (34.7)	76 (41.5)	934 (15.3)	925 (17.2)	9 (1.2)
Cancer	135 (13.0)	124 (14.5)	11 (6.0)	649 (10.6)	585 (10.9)	64 (8.9)
Asthma	165 (15.9)	139 (16.2)	26 (14.2)	1579 (25.9)*	1404 (26.1)*	175 (24.2)*
Chronic obstructive pulmonary disease	283 (27.2)	241 (28.1)	42 (23.0)			
Sleep apnoea	49 (4.7)	42 (4.9)	7 (3.8)			
Cardiovascular disease	567 (54.5)	479 (55.9)	88 (48.1)	3033 (49.7)	2719 (50.6)	314 (43.5)
Hypertension	661 (63.6)	539 (62.9)	122 (66.7)	Not available		
Diabetes without complications	258 (24.8)	202 (23.6)	56 (30.6)	1794 (29.4)	1559 (29.0)	235 (32.5)
Diabetes with complications	112 (10.8)	103 (12.0)	9 (4.9)	Not available		
Peptic ulcer	29 (2.8)	26 (3.0)	3 (1.6)	Not available		
Liver disease	71 (6.8)	60 (7.0)	11 (6.0)	Not available		
Rheumatic/inflammatory disease	51 (4.9)	49 (5.7)	2 (1.1)	Not available		
Thyroid disorder	107 (10.3)	100 (11.7)	7 (3.8)	Not available		
ISARIC comorbidity score				Not applicable		
0	111 (10.7)	92 (10.7)	19 (10.4)			
1	234 (22.5)	176 (20.5)	58 (31.7)			
≥2	695 (66.8)	589 (68.7)	106 (57.9)			
Symptoms, n (%)						
Breathlessness	559 (59.2)	424 (54.7)	135 (79.9)	Not available		
Chest pain	39 (4.1)	29 (3.7)	10 (5.9)	Not available		
Cough	538 (57.0)	415 (53.5)	123 (72.8)	4259 (69.8)	3646 (67.8)	613 (84.9)
Fever	465 (49.3)	357 (46.1)	108 (63.9)	3212 (52.7)	2694 (50.1)	518 (71.7)
Headache	42 (4.4)	30 (3.9)	12 (7.1)	Not available		
Malaise	186 (19.7)	144 (18.6)	42 (24.9)	Not available		
New onset diarrhoea or vomiting	56 (5.9)	49 (6.3)	7 (4.1)	Not available		
Sputum	84 (8.9)	60 (7.7)	24 (14.2)	Not available		
Delirium	80 (8.5)	77 (9.9)	3 (1.8)	1160 (20.1)	1085 (21.3)	75 (10.9)
Outcomes						
Died within 28 days of admission	288 (27.7)	237 (27.7)	51 (27.9)	1668 (27.3)	1423 (26.5)	245 (33.9)
ITU admission within 28 days	183 (17.6)	0 (0.0)	183 (100.0)	722 (11.8)	0 (0.0)	722 (100.0)

*In CovidCollab, asthma, chronic obstructive pulmonary disease and sleep apnoea were combined as 'respiratory diseases'; data on the individual conditions was not available, therefore the n (%) given is for all respiratory diseases.

For peer review only

Supplementary Table 2. Area under the receiver operating characteristic curve (AUROC) for each of the models assessed in the derivation dataset (University Hospitals Birmingham) for predicting death within 28 days of hospital admission

Model	Continuous data	Missing continuous data	Candidate predictors included	AUROC (95% CI)
Logistic	Categorical	Imputed	All	0.743 (0.702-0.783)
LASSO	Categorical	Imputed	All	0.755 (0.720-0.791)
GBM	Categorical	Imputed	All	0.758 (0.719-0.797)
Logistic	Categorical	Imputed	Stepwise selection	0.779 (0.744-0.813)
Logistic	Categorical	Imputed	All plus comorbidities	0.730 (0.688-0.773)
Logistic	Categorical	Imputed	Demographic only	0.709 (0.676-0.742)
Logistic	Categorical	Missing category	All	0.773 (0.740-0.806)
Logistic	Categorical	Missing category	Demographic only	0.705 (0.670-0.740)
Logistic	Categorical	Missing category	Stepwise selection	0.805 (0.776-0.835)
LASSO	Categorical	Missing category	All	0.786 (0.750-0.823)
GBM	Categorical	Missing category	All	0.778 (0.745-0.811)
Logistic	Continuous	Imputed	All	0.768 (0.734-0.802)
Logistic	Continuous	Imputed	All with splines*	0.730 (0.652-0.807)
LASSO	Continuous	Imputed	All	0.778 (0.745-0.811)
LASSO	Continuous	Imputed	All with splines*	0.786 (0.755-0.817)
GBM	Continuous	Imputed	All	0.783 (0.750-0.816)
GBM	Continuous	Imputed	All with splines*	0.775 (0.742-0.809)
Logistic	Continuous	Imputed	Stepwise selection	0.794 (0.765-0.824)
Logistic	Continuous	Imputed	Stepwise selection with splines*	0.792 (0.762-0.822)

All = all demographic, symptoms, vital signs/physiological measures, and laboratory test result candidate predictors. *Splines were used to model continuous variables where non-liner associations with the outcome were observed. LASSO = least absolute shrinkage and selection operator; GBM = gradient boosting machine.

For peer review only

Supplementary Table 3. Logistic regression model coefficients for mortality within 28 days of admission in the derivation dataset (University Hospitals Birmingham)

Logistic regression equation:

$$\ln\left(\frac{p}{1-p}\right) = \beta_0 + \sum_{i=1}^{m} \beta_i x_i$$

Where p is probability, β_0 is the intercept (constant term), and β_i are the coefficients for each of the predictors x_i.

To calculate probability (p):

$$p = \frac{1}{1 + e^{-(\beta_0 + \sum_{i=1}^{m} \beta_i x_i)}}$$

Predictor	Coefficient (95% CI)
Intercept	-4.143 (-5.269--3.018)
Age category (years), n (%)	
<30	-13.985 (-746.83-718.86)
30-39	-0.609 (-1.929-0.710)
40-49	-1.353 (-2.557--0.149)
50-59	Ref
60-69	0.201 (-0.438-0.839)
70-79	0.978 (0.364-1.591)
80-89	1.353 (0.691-2.014)
≥90	1.705 (0.960-2.451)
Breathlessness	0.310 (-0.086-0.706)
Sputum	0.383 (-0.187-0.952)
Temperature (Celsius), n (%)	
<37.8	Ref
≥37.8	0.286 (-0.161-0.733)
Systolic blood pressure (mm Hg), n (%)	
<140	Ref
≥140	-0.340 (-0.695-0.016)
Respirations (per minute), n (%)	
<20	Ref
≥20	0.405 (0.035-0.775)
Oxygen saturation (%), n (%)	
<80	1.042 (-0.503-2.588)
80-88	1.425 (0.699-2.151)
89-93	0.332 (-0.085-0.749)
≥94	Ref
Portable oxygen concentrator fraction of inspired oxygen (%), n (%)	
≤0.28	Ref

For peer review only

0.28-0.49	0.624 (-0.069-1.316)
≥0.5	0.564 (-0.021-1.149)
pH, n (%)	
<7.30	0.344 (-0.308-0.997)
7.30-7.34	-0.347 (-0.931-0.238)
7.35-7.44	Ref
≥7.45	0.088 (-0.386-0.561)
White blood cell count (10^9/l), n (%)	
<3.9	-0.872 (-1.649--0.095)
3.9-10.8	Ref
≥10.9	0.158 (-0.252-0.568)
Platelets (10^9/l), n (%)	
<150	0.442 (-0.007-0.892)
150-399	Ref
≥400	-0.393 (-1.051-0.265)
C-reactive protein (mg/l), n (%)	
<10	Ref
10-99	0.613 (-0.312-1.538)
≥100	1.013 (0.109-1.917)
Glucose (mmol/l), n (%)	
<7.8	Ref
7.8-8.4	0.559 (-0.016-1.135)
≥8.5	0.359 (-0.005-0.723)
Alkaline phosphatase (U/l), n (%)	
<130	Ref
≥130	0.327 (-0.095-0.750)
Urea (mmol/l), n (%)	
<7.8	Ref
≥7.8	0.386 (0.035-0.738)
Corrected calcium (mmol/l), n (%)	
<2.2	0.575 (0.092-1.058)
2.2-2.5	Ref
≥2.6	1.057 (0.265-1.849)
Eosinophils (10^9/l), n (%)	
≤0.4	Ref
>0.4	-1.512 (-3.654-0.630)
Frailty score, n (%)	
1-3	Ref
4-6	0.345 (-0.231-0.920)
7-9	0.738 (0.113-1.363)

For peer review only

Supplementary Table 4. Area under the receiver operating characteristic curve (AUROC) for each of the models assessed in the derivation dataset (University Hospitals Birmingham) for predicting intensive therapy unit admission within 28 days of hospital admission

Model	Continuous data	Missing continuous data	Candidate predictors included	AUROC (95% CI)
Logistic	Categorical	Imputed	All	0.854 (0.824-0.885)
LASSO	Categorical	Imputed	All	0.877 (0.848-0.906)
GBM	Categorical	Imputed	All	0.874 (0.844-0.904)
Logistic	Categorical	Imputed	Stepwise selection	0.893 (0.864-0.922)
Logistic	Categorical	Imputed	All plus comorbidities	0.872 (0.835-0.909)
Logistic	Categorical	Imputed	Demographic only	0.766 (0.732-0.799)
Logistic	Categorical	Missing category	All	0.909 (0.858-0.960)
Logistic	Categorical	Missing category	Demographic only	0.764 (0.731-0.798)
Logistic	Categorical	Missing category	Stepwise selection	0.960 (0.944-0.976)
LASSO	Categorical	Missing category	All	0.961 (0.946-0.976)
GBM	Categorical	Missing category	All	0.956 (0.939-0.973)
Logistic	Continuous	Imputed	All	0.869 (0.837-0.901)
Logistic	Continuous	Imputed	All with splines*	0.892 (0.865-0.920)
LASSO	Continuous	Imputed	All	0.888 (0.860-0.915)
LASSO	Continuous	Imputed	All with splines*	0.903 (0.878-0.928)
GBM	Continuous	Imputed	All	0.896 (0.869-0.923)
GBM	Continuous	Imputed	All with splines*	0.899 (0.874-0.924)
Logistic	Continuous	Imputed	Stepwise selection	0.895 (0.869-0.922)
Logistic	Continuous	Imputed	Stepwise selection with splines*	0.910 (0.886-0.933)

All = all demographic, symptoms, vital signs/physiological measures, and laboratory test result candidate predictors. *Splines were used to model continuous variables where non-liner associations with the outcome were observed. LASSO = least absolute shrinkage and selection operator; GBM = gradient boosting machine.

For peer review only

Supplementary Table 5. Logistic regression model coefficients for intensive therapy unit admission within 28 days of hospital admission in the derivation dataset (University Hospitals Birmingham)

Predictor	Coefficient (95% CI)
Intercept	-0.459 (-2.077-1.159)
Age category (years), n (%)	
<30	-0.635 (-1.913-0.644)
30-39	-0.603 (-1.655-0.449)
40-49	-0.291 (-1.050-0.469)
50-59	Ref
60-69	-0.734 (-1.390--0.078)
70-79	-1.296 (-2.005--0.588)
80-89	-3.445 (-4.693--2.197)
≥90	-17.026 (-1075.95-1041.90)
Sex (male)	0.720 (0.214-1.226)
Fever	0.277 (-0.319-0.873)
New onset diarrhoea or vomiting	-0.937 (-2.090-0.217)
Temperature (Celsius), n (%)	
<37.8	Ref
≥37.8	0.619 (0.059-1.178)
Heart rate category (beats per minute), n (%)	
<80	Ref
80-99	-0.634 (-1.270-0.003)
≥100	-0.902 (-1.594--0.209)
Respirations (per minute), n (%)	
<20	Ref
≥20	0.799 (0.151-1.447)
Portable oxygen concentrator fraction of inspired oxygen (%), n (%)	
≤0.28	Ref
0.28-0.49	1.244 (0.357-2.131)
≥0.5	1.505 (0.686-2.324)
pH, n (%)	
<7.30	1.809 (0.861-2.757)
7.30-7.34	-0.104 (-0.958-0.750)
7.35-7.44	Ref
≥7.45	0.530 (-0.063-1.123)
White blood cell count (10^9/l), n (%)	
<3.9	-0.650 (-1.657-0.356)
3.9-10.8	Ref
≥10.9	1.372 (0.717-2.027)
C-reactive protein (mg/l), n (%)	
<10	Ref
10-99	0.227 (-0.913-1.367)
≥100	0.537 (-0.657-1.732)
Albumin (g/l), n (%)	
<25	Ref

For peer review only

25-34	-0.881 (-1.550--0.212)
≥35	-1.037 (-1.936--0.138)
Monocytes (10^9/l), n (%)	
<0.2	Ref
0.2-0.8	0.101 (-0.733-0.936)
>0.8	-0.972 (-2.067-0.122)
eGFR (ml/min), n (%)	
<30 (Stage 4 or above)	-0.731 (-1.506-0.045)
30-59 (Stage 3)	0.323 (-0.435-1.082)
60-89 (Stage 2)	-0.164 (-0.731-0.402)
>90 (Normal or high)	Ref
Frailty score, n (%)	
1-3	Ref
4-6	-0.522 (-1.047-0.002)
7-9	-2.080 (-3.117--1.044)
Glasgow Coma Scale score, n (%)	
<15	Ref
15	-1.348 (-1.896--0.801)

For peer review only

Supplementary Table 6. Logistic regression model coefficients for the external validation/reduced model (UHB-R) for mortality within 28 days of hospital admission (developed in University Hospitals Birmingham data and validated in CovidCollab)

Predictor	Coefficient (95% CI)
Intercept	-3.730 (-4.848--2.611)
Age category (years), n (%)	
<30	-14.108 (-766.78-738.56)
30-39	-0.617 (-1.922-0.689)
40-49	-1.333 (-2.487--0.178)
50-59	Ref
60-69	0.203 (-0.421-0.828)
70-79	0.925 (0.324-1.526)
80-89	1.204 (0.569-1.838)
≥90	1.590 (0.866-2.313)
Temperature (Celsius), n (%)	
<37.8	Ref
≥37.8	0.336 (-0.101-0.773)
Systolic blood pressure (mm Hg), n (%)	
<140	Ref
≥140	-0.353 (-0.691--0.015)
Respirations (per minute), n (%)	
<20	Ref
≥20	0.462 (0.120-0.804)
Oxygen saturation (%), n (%)	
<80	1.028 (-0.525-2.580)
80-88	1.491 (0.788-2.194)
89-93	0.480 (0.084-0.876)
≥94	Ref
Portable oxygen concentrator fraction of inspired oxygen (%), n (%)	
≤0.28	Ref
0.28-0.49	0.667 (0.027-1.307)
≥0.5	0.588 (0.028-1.148)
pH, n (%)	
<7.30	0.412 (-0.217-1.042)
7.30-7.34	-0.262 (-0.809-0.284)
7.35-7.44	Ref
≥7.45	0.186 (-0.269-0.640)
C-reactive protein (mg/l), n (%)	
<10	Ref
10-99	0.721 (-0.172-1.615)
≥100	1.167 (0.301-2.033)
Urea (mmol/l), n (%)	
<7.8	Ref
≥7.8	0.494 (0.159-0.828)
Frailty score, n (%)	

For peer review only

1-3	Ref
4-6	0.333 (-0.228-0.895)
7-9	0.685 (0.072-1.297)

For peer review only

Supplementary Table 7. Logistic regression model coefficients for the external validation/reduced model (UHB-R) model for intensive therapy unit admission within 28 days of hospital admission (developed in University Hospitals Birmingham data and validated in CovidCollab)

Predictor	Coefficient (95% CI)
Intercept	-2.482 (-4.012--0.951)
Age category (years), n (%)	
<30	-0.688 (-1.859-0.483)
30-39	-0.534 (-1.531-0.464)
40-49	-0.253 (-0.975-0.470)
50-59	Ref
60-69	-0.648 (-1.274--0.021)
70-79	-1.080 (-1.745--0.416)
80-89	-3.035 (-4.233--1.837)
≥90	-16.756 (-1103.28-1069.77)
Sex (male)	0.608 (0.112-1.105)
Fever	0.279 (-0.267-0.824)
Respirations (per minute), n (%)	
<20	Ref
≥20	0.648 (0.052-1.245)
Portable oxygen concentrator fraction of inspired oxygen (%), n (%)	
≤0.28	Ref
0.28-0.49	1.205 (0.392-2.018)
≥0.5	1.703 (1.011-2.396)
pH, n (%)	
<7.30	1.838 (0.917-2.759)
7.30-7.34	0.125 (-0.655-0.905)
7.35-7.44	Ref
≥7.45	0.621 (0.047-1.196)
C-reactive protein (mg/l), n (%)	
<10	Ref
10-99	-0.046 (-1.116-1.023)
≥100	0.456 (-0.632-1.544)
Neutrophil:lymphocyte ratio, n (%)	
<2.21	Ref
2.21-4.82	0.959 (-0.159-2.077)
>4.82	1.305 (0.212-2.399)
eGFR (ml/min), n (%)	
<30 (Stage 4 or above)	-0.598 (-1.331-0.136)
30-59 (Stage 3)	0.300 (-0.385-0.985)
60-89 (Stage 2)	-0.253 (-0.780-0.275)
>90 (Normal or high)	Ref
Frailty score, n (%)	
1-3	Ref
4-6	-0.475 (-0.979-0.028)
7-9	-1.991 (-3.025--0.957)

For peer review only

Glasgow Coma Scale score, n (%)	
<15	Ref
15	-1.310 (-1.821--0.798)

For peer review only

Supplementary Table 8. Sensitivity analysis – complete case analysis and exploration of different numbers of missing candidate predictors: Area under the receiver operating characteristic curve (AUROC) in the University Hospitals Birmingham dataset for predicting mortality and intensive therapy unit admission within 28 days of hospital admission

Number of missing predictor variables per patient	n	Outcome	
		Death	ITU
0	224	0.696 (0.597-0.795)	0.892 (0.844-0.940)
0-1	471	0.760 (0.708-0.812)	0.880 (0.846-0.915)
0-2	564	0.763 (0.715-0.812)	0.877 (0.842-0.911)
0-5	643	0.756 (0.712-0.799)	0.886 (0.854-0.918)
0-10	911	0.772 (0.739-0.806)	0.885 (0.857-0.914)

Supplementary Table 9. Sensitivity analysis – stratification by gender and age category: Area under the receiver operating characteristic curve (AUROC) in the University Hospitals Birmingham dataset for predicting mortality and intensive therapy unit admission within 28 days of hospital admission, using the reduced UHB-R model

Model		Outcome	
Derivation/Train	Test	Death	ITU
**Gender-stratified**			
UHB-R (fitted on all patients)	Male	0.775 (0.726-0.823)	0.897 (0.856-0.937)
UHB-R (fitted on all patients)	Female	0.755 (0.706-0.804)	0.873 (0.833-0.913)
**Age-stratified**			
UHB-R (fitted on all patients)	≤60 years	0.730 (0.638-0.823)	0.845 (0.761-0.930)
UHB-R (fitted on all patients)	>60 years	0.778 (0.722-0.834)	0.897 (0.864-0.930)

Supplementary figure legends:

Supplementary Figure 1. Calibration plot (observed probability (y-axis) against predicted probability (x-axis)) for ISARIC 4C score in UHB dataset (mortality outcome).

Supplementary Figure 2. Exploration of non-linear relationships between continuous predictors and 28-day mortality using general additive models (GAM); splines (continuous smoothed predictors) were included in the models where a non-linear relationship was observed.

Supplementary Figure 3. Exploration of non-linear relationships between continuous predictors and intensive therapy unit admission using general additive models (GAM); splines (continuous smoothed predictors) were included in the models where a non-linear relationship was observed.

Supplementary Figure 4. Forest plot of logistic regression coefficients for days 0-7 from admission for mortality outcome.

For peer review only

TRIPOD Checklist: Prediction Model Development and Validation

Section/Topic	Item		Checklist Item	Page
**Title and abstract**				
Title	1	D;V	Identify the study as developing and/or validating a multivariable prediction model, the target population, and the outcome to be predicted.	1
Abstract	2	D;V	Provide a summary of objectives, study design, setting, participants, sample size, predictors, outcome, statistical analysis, results, and conclusions.	3
**Introduction**				
Background and objectives	3a	D;V	Explain the medical context (including whether diagnostic or prognostic) and rationale for developing or validating the multivariable prediction model, including references to existing models.	4
	3b	D;V	Specify the objectives, including whether the study describes the development or validation of the model or both.	5
**Methods**				
Source of data	4a	D;V	Describe the study design or source of data (e.g., randomized trial, cohort, or registry data), separately for the development and validation data sets, if applicable.	5
	4b	D;V	Specify the key study dates, including start of accrual; end of accrual; and, if applicable, end of follow-up.	5-6
Participants	5a	D;V	Specify key elements of the study setting (e.g., primary care, secondary care, general population) including number and location of centres.	5
	5b	D;V	Describe eligibility criteria for participants.	5
	5c	D;V	Give details of treatments received, if relevant.	n/a
Outcome	6a	D;V	Clearly define the outcome that is predicted by the prediction model, including how and when assessed.	5
	6b	D;V	Report any actions to blind assessment of the outcome to be predicted.	n/a
Predictors	7a	D;V	Clearly define all predictors used in developing or validating the multivariable prediction model, including how and when they were measured.	6, 9
	7b	D;V	Report any actions to blind assessment of predictors for the outcome and other predictors.	n/a
Sample size	8	D;V	Explain how the study size was arrived at.	5
Missing data	9	D;V	Describe how missing data were handled (e.g., complete-case analysis, single imputation, multiple imputation) with details of any imputation method.	7
Statistical analysis methods	10a	D	Describe how predictors were handled in the analyses.	6-7
	10b	D	Specify type of model, all model-building procedures (including any predictor selection), and method for internal validation.	6-7
	10c	V	For validation, describe how the predictions were calculated.	7-8
	10d	D;V	Specify all measures used to assess model performance and, if relevant, to compare multiple models.	7
	10e	V	Describe any model updating (e.g., recalibration) arising from the validation, if done.	7-8
Risk groups	11	D;V	Provide details on how risk groups were created, if done.	8
Development vs. validation	12	V	For validation, identify any differences from the development data in setting, eligibility criteria, outcome, and predictors.	n/a
**Results**				
Participants	13a	D;V	Describe the flow of participants through the study, including the number of participants with and without the outcome and, if applicable, a summary of the follow-up time. A diagram may be helpful.	9, 11
	13b	D;V	Describe the characteristics of the participants (basic demographics, clinical features, available predictors), including the number of participants with missing data for predictors and outcome.	9, Table 1
	13c	V	For validation, show a comparison with the development data of the distribution of important variables (demographics, predictors and outcome).	Table 1
Model development	14a	D	Specify the number of participants and outcome events in each analysis.	9, 11, Table 1
	14b	D	If done, report the unadjusted association between each candidate predictor and outcome.	n/a
Model specification	15a	D	Present the full prediction model to allow predictions for individuals (i.e., all regression coefficients, and model intercept or baseline survival at a given time point).	Suppl. Tables 3, 5-7
	15b	D	Explain how to the use the prediction model.	13
Model performance	16	D;V	Report performance measures (with CIs) for the prediction model.	10-12
Model-updating	17	V	If done, report the results from any model updating (i.e., model specification, model performance).	11-12
**Discussion**				
Limitations	18	D;V	Discuss any limitations of the study (such as nonrepresentative sample, few events per predictor, missing data).	14
Interpretation	19a	V	For validation, discuss the results with reference to performance in the development data, and any other validation data.	13-14
	19b	D;V	Give an overall interpretation of the results, considering objectives, limitations, results from similar studies, and other relevant evidence.	15
Implications	20	D;V	Discuss the potential clinical use of the model and implications for future research.	15
**Other information**				
Supplementary information	21	D;V	Provide information about the availability of supplementary resources, such as study protocol, Web calculator, and data sets.	Suppl. Tables
Funding	22	D;V	Give the source of funding and the role of the funders for the present study.	16

For peer review only

TRIPOD Checklist: Prediction Model Development and Validation

*Items relevant only to the development of a prediction model are denoted by D, items relating solely to a validation of a prediction model are denoted by V, and items relating to both are denoted D;V. We recommend using the TRIPOD Checklist in conjunction with the TRIPOD Explanation and Elaboration document.

For peer review only
